# Exploring anticancer activity of structurally modified benzylphenoxyacetamide (BPA); I: Synthesis strategies and computational analyses of substituted BPA variants with high anti-glioblastoma potential

**DOI:** 10.1038/s41598-019-53207-0

**Published:** 2019-11-19

**Authors:** Joanna Stalinska, Lisa Houser, Monika Rak, Susan B. Colley, Krzysztof Reiss, Branko S. Jursic

**Affiliations:** 1 Department of Chemistry, University of New Orleans, New Orleans, LA 70148 United States; 2 Stepharm llc., PO Box 24220, New Orleans, LA 70184 United States; 3 Neurological Cancer Research, Stanley S. Scott Cancer Center, Department of Medicine, LSU Health Sciences Center, New Orleans, LA 70112 USA; 4 Department of Cell Biology, Faculty of Biochemistry, Biophysics and Biotechnology, Jagiellonian University, Cracow, Poland

**Keywords:** Drug discovery and development, CNS cancer

## Abstract

Structural variations of the benzylphenoxyacetamide (BPA) molecular skeleton were explored as a viable starting point for designing new anti-glioblastoma drug candidates. Hand-to-hand computational evaluation, chemical modifications, and cell viability testing were performed to explore the importance of some of the structural properties in order to generate, retain, and improve desired anti-glioblastoma characteristics. It was demonstrated that several structural features are required to retain the anti-glioblastoma activity, including a carbonyl group of the benzophenone moiety, as well as 4′-chloro and 2,2-dimethy substituents. In addition, the structure of the amide moiety can be modified in such a way that desirable anti-glioblastoma and physical properties can be improved. Via these structural modifications, more than 50 compounds were prepared and tested for anti-glioblastoma activity. Four compounds were identified (HR28, HR32, HR37, and HR46) that in addition to HR40 (PP1) from our previous study, have been determined to have desirable physical and biological properties. These include high glioblastoma cytotoxicity at low μM concentrations, improved water solubility, and the ability to penetrate the blood brain barrier (BBB), which indicate a potential for becoming a new class of anti-glioblastoma drugs.

## Introduction

Glioblastoma is the most aggressive and prevalent malignancy of the central nervous system (CNS), with a median patient survival rate of about 18 months^1–4^. Surprisingly, the most effective way to increase survival of glioblastoma patients is still extensive surgical resection. However, in many instances, this approach is not feasible due to the tumor’s location and its infiltration of highly specialized brain areas^5^. The current standard of care therapies include maximal surgical resection, followed by radiotherapy plus concomitant and maintenance treatment with temozolomide (TMZ), which is one of the few anticancer drugs capable of crossing the blood brain barrier (BBB)^6^. Unfortunately, TMZ-treated tumors develop TMZ-resistance, and recurrent glioblastomas are practically incurable^7–9^. Moreover, numerous clinical trials targeting a variety of glioblastoma-specific pathways, as well as, those testing immune checkpoint inhibitors, have been implemented, but have failed to produce a positive outcome in glioblastoma patients^2,10,11^.

Therapeutic strategies that include TMZ in combination with other drugs have been also explored. For instance, glioblastomas are characterized by exaggerated lipogenesis, enhanced LDL and cholesterol uptake, and extensive phagocytosis and exosome formation^12–15^. All of these processes require high cholesterol metabolism and uptake for a continuous biogenesis of cellular membranes. Therefore, it does not come as a surprise that a combination of cholesterol lowering drugs with TMZ might be a good approach for glioblastoma treatment^16^. One lipid-lowering drug that has attracted attention as a possible candidate for an anticancer regimen is fenofibrate (**FF**)^17–22^. **FF** is a common lipid-lowering pro-drug that following its processing by blood and tissue esterases is converted to fenofibric acid **(FAA)**, a potent agonist of peroxisome proliferator activated receptor alpha (PPARα) that upregulates fatty acid utilization and attenuates glycolysis^23–26^. Although activation of PPARα may explain some of the observed anticancer effects, glioblastoma cells treated with PPARα siRNA retain sensitivity to **FF**, indicating a PPARα-independent mechanism of **FF** anticancer action. Indeed, our previously published data demonstrate that unprocessed **FF** (ester) accumulates in mitochondrial membrane fraction triggering a severe and immediate inhibition of mitochondrial respiration, severe decline in intracellular ATP, and is followed by a massive tumor cell death^27^. In addition to FF, interesting anticancer effects of other lipid lowering drugs, fibrates and statins, have also been reported^28–34^. A ten-year all-cause mortality study involving 7,722 patients treated with different fibrates revealed that the use of these metabolic compounds was associated with a significantly lower total mortality and reduced probability of death from cancer^35^. In cell culture and in animal studies, various members of the fibrate family demonstrated a broad range of anticancer activities^17,20,31,32,34,36–40^. These multiple reports encouraged clinical trials in which chronic administration of low doses of FF was tested along with chemotherapeutic agents, minimizing their toxicity and acute side effects in patients with recurrent brain tumors and leukemia^41,42^.

Other groups also demonstrated that FF could have PPAR-independent cellular effects including: PPAR-independent activation of GDF15^23^; effects of FF on cell membrane fluidity^43^; and the FF-induced inhibition of mitochondrial respiration in isolated cardiac and liver mitochondria^44,45^. Therefore, a growing line of evidence supports the interaction of unprocessed FF (ester) with biological membranes, which could be a reason for the observed strong anticancer activity of this lipid-lowering drug. However, **FF** does not cross the BBB, and is quickly processed by the blood and tissue esterases to form fenofibric acid (FFA). This acid functions as a potent peroxisome proliferator activated receptor α (PPARα) agonist, however, it is no longer effective in triggering tumor cell death^27,46^.

**Figure 1:**
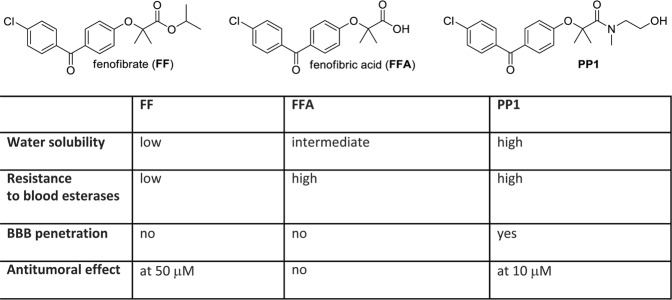
Comparison between **FF**, **FFA**, and **PP1** (modified **FF**^47^) structural and functional (anti-cancer) properties. The information regarding the compounds water solubility, stability in human blood, penetration of the blood brain barrier (BBB), and *in vitro* cytotoxicity were previously reported^27,46,47^.

We have made several chemical modifications to the **FF** structure in order to improve the prospective anticancer drug stability, water solubility, tissue penetration, and ultimately, the anti-glioblastoma efficacy. One of the resulting compounds, **PP1** (Fig. 1), triggered extensive glioblastoma cell death *in vitro* at concentrations almost 5-fold lower than **FF**^47^. Similar to FF, **PP1** inhibited mitochondrial respiration, and demonstrated improved water solubility, BBB penetration, and resistance to blood esterases^47^. However, **PP1** accumulation in the mouse brain tumor tissue (following oral administration) varies between 5 and 6 μM and may not be sufficient to exert a highly effective anti-glioblastoma activity *in vivo*^47^. Therefore, we further explored the anti-tumoral contribution of the specific chemical moieties in the **FF** and **PP1** structures, to improve the compound anticancer efficacy and its ability for more effective accumulation within the brain tumor tissue. As a result, 50 additional compounds were generated and analyzed in this study.

## Results and Discusson

### Overall chemical design of therapeutic compounds

**Figure 2:**
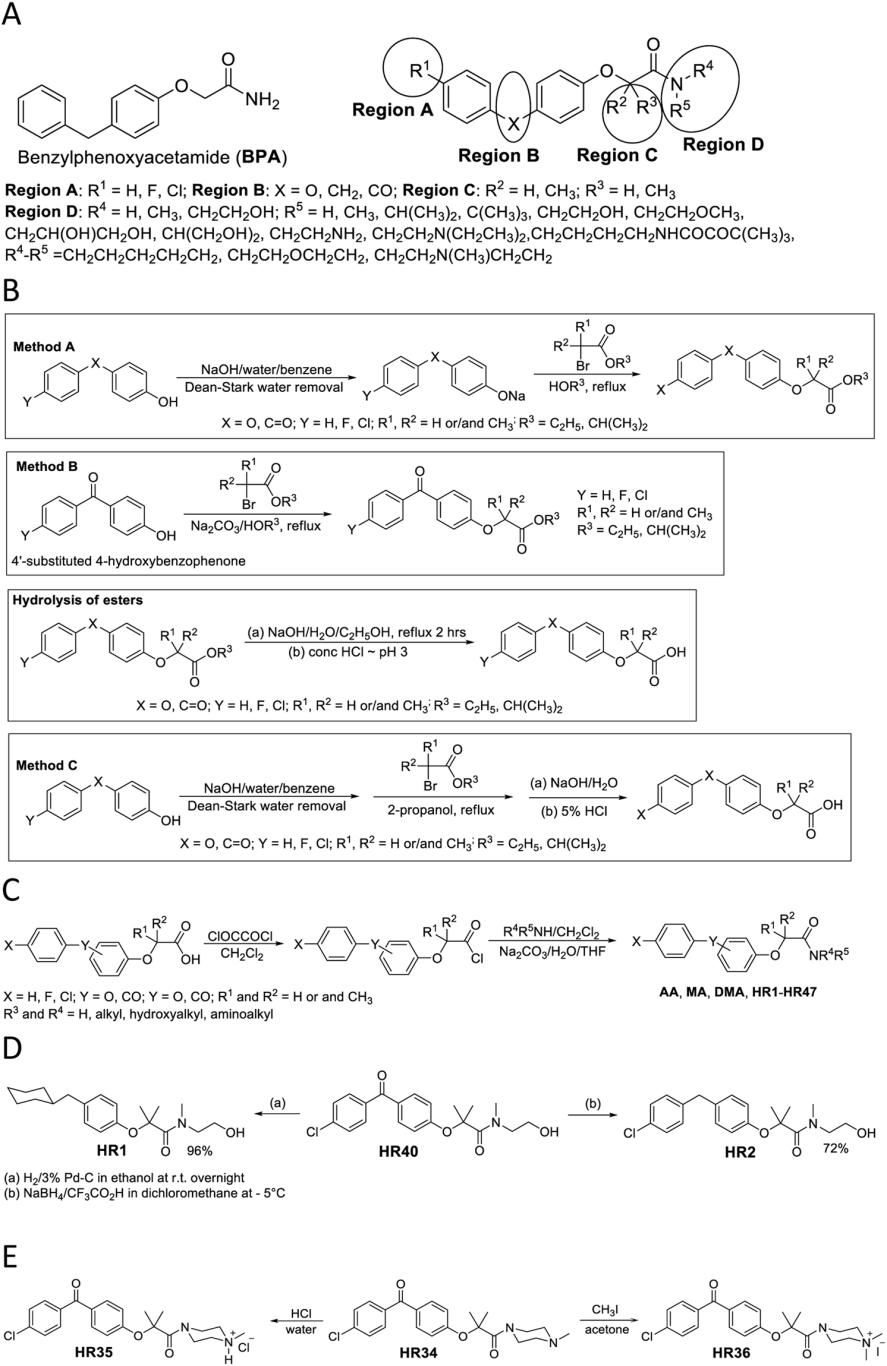
Panel (A) Regions of the **BPA** skeleton selected for modification (circles). These regions were subsequently modified as per legend in search of the optimal anti-glioblastoma drug. Panel (B) Schematic illustration of the procedure to develop esters of substituted phenoxyacetic esters and acids. Panel (C) Schematic illustration of the procedure for substituted phenoxyacetamides. Panel (D) Schematic illustration of the selective reduction of **HR40 (PP1)** to obtain **HR1** and **HR2**. Panel (E) Schematic illustration of the procedure to develop ammonium salts of **HR34**.

The basic molecular skeleton of fenofibrate (**FF)** contains a benzylphenoxyacetate structural arrangement (Fig. 1). Although **FF** shows promising anti-glioblastoma activity at 50 µM, this compound is an isopropyl ester that is promptly hydrolyzed into fenofibric acid (**FFA)** by blood and tissue esterases^46^. An additional disadvantage of **FF** is that it has low water solubility, and a relatively high concentration is required for its anti-tumoral activity (50 μM)^27^. Therefore, we have selected the benzylphenoxyacetamide (**BPA**) molecular skeleton as a basis for designing new more potent anti-glioblastoma compounds due to its structural similarity to **FF**, as well as its increased water solubility and higher resistance to hydrolysis (Figs. 1 and 2A).

Four specific regions of the **BPA** were subsequently designated for chemical modification in order to determine how the nature of the substitutions may alter its anti-glioblastoma activity (Fig. 2A). Structural variations to the chosen regions include exchange of halogens (region A); addition or removal of oxygen, methylene, carbonyl groups (region B); addition or removal of hydrogens, one methyl, or two methyl groups (region C); and replacement, removal or addition of one alkyl, two alkyl, one hydroxyalkyl, two hydroxyalkyl, and alkyl with primary, secondary, or tertiary group (region D).

All preparations evaluated in this study were initiated with either substituted phenoxyphenols or benzoylphenols (Fig. 2B; Methods A–C). The phenols were first converted into their corresponding sodium salts. Due to the differences in acidity of these two groups of compounds (pKa of approximately 10 for phenoxyphenols and 8 for benzoylphenols), two different bases were used. The first base, sodium hydroxide in water/benzene was a sufficiently strong base (pKa ~15.7) and was used for preparation of sodium salts of both groups of phenols (Fig. 2B; Method A). A Deen-Stark distilling receiver was used to azeotropically remove water. Therefore, a dry white powdery sodium phenoxide resulted and was used in a second step which adds bromoalkanoic esters to perform nucleophilic substitution. Isolated yields were almost quantitative. However, for the more acidic benzoylphenols, one-pot synthesis in alcohol with anhydrous sodium carbonate was also introduced (Fig. 2B; Method B). This approach requires longer time but is simpler, safer, more economical and it is the method of choice for the preparation of benzoylphenoxyethanoic acid.

The next step is the preparation of phenoxyethanoic acids from the esters. This was accomplished in one-pot synthesis without isolation of the intermediate ester (Fig. 2B; Method C). In this method, the phenol substrate was first converted into its sodium salt with sodium hydroxide via Dean-Stark distillation with molecular sieves^48^, followed by a reaction with the 2-bromaethanoic ester in isopropanol, and finally a hydrolysis step with aqueous sodium hydroxide in isopropanol. These products are easily purified by an acid-base extraction. This is the preferred method for direct preparation of phenoxyethanoic acids, which are the intermediates for preparation of targeted amides. Isolated yields are between 85 to 95%.

The starting point for the preparation of each **BPA** modification in this study is the benzylphenoxyacetic acid derivative. For amides, although there is a plethora of synthetic methods for transformation of acids into amides, many of these methods cannot be successfully applied to the preparation of all the amides included in this study. For instance, employing a well-established amide preparation that utilizes DCC and EDC based activation agents^49^ for direct carboxylic acid conversion to amide could not be used to create the amide of 2,2-dimethylbenzylphenoxyacetic acid. This is due to a combination of steric hindrance and lower reactivity of the activated carboxylic acid. However, when the corresponding carboxylic acid chloride is used, almost quantitative conversion of carboxylic acid into targeted amides is achieved (Fig. 2C).

For formation of reduced forms of the targeted amides, preparation was performed *via* selective reduction (Fig. 2D). For one of the reduction methods, catalytic hydrogenation, such as that performed with 3% palladium (Pd) on carbon, removes halogens, reduces the carbonyl group to a methylene group, and reduces the aromatic ring that contains chlorine. This reaction is carried out at room temperature and in a hydrogen environment at atmospheric pressure. The isolated yield is almost quantitative. One product (HR1) was prepared in this way by reducing **HR40 (PP1)** (Fig. 2D). Selective reduction of the carbonyl group created **HR2** from **HR40** and was accomplished by using a combination of sodium borohydride and trifloroacidic acid at 5 °C (Fig. 2D). In this case, the product had to be separated from the starting material by chromatography, so the isolated yield was only 72%.

To further increase the water solubility of basic **BPA**, ammonium salts were prepared. Hydrochloric salts were prepared by simple mixing of the corresponding **BPA** with concentrated hydrochloric acid followed by water evaporation. Then, methylation of the basic **BPA** was performed with acetone as the solvent, and methyl iodide as the methylating reagent. The product, **HR36**, was crystalized directly from the reaction mixture (Fig. 2E).

### Biological, chemical and computational testing of therapeutic compounds

As mentioned above, it was demonstrated that **FF** possesses anti-glioblastoma activity. However, there are several **FF** properties that make it impractical for anticancer treatment^27,46^. In our previous study, we reported that some amide derivatives of **FF**, including **PP1**, were more potent in eliminating glioblastoma cells than **FF**^47^. These amides belong to the large family of **BPA**^50^.

**Figure 3:**
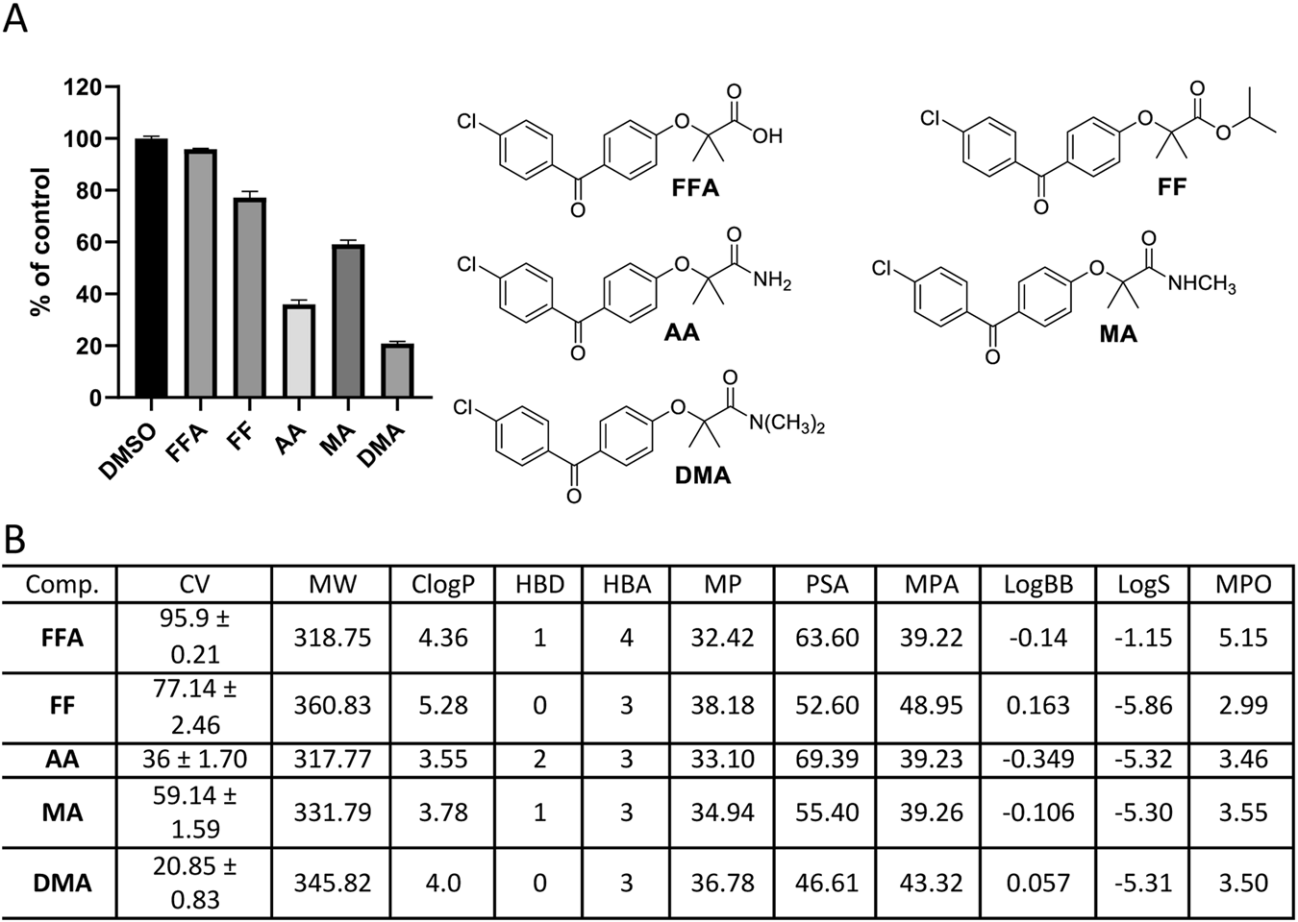
Anti-glioblastoma activity and computed physical properties of fenofibrate (**FF**) and its simple amides. Panel (A) Cell viability (MTT assay) following exposure to the indicated derivatives of **FF** (25 μM, for 72 hrs). (Panel B) Computed physical properties of fenofibrate and its simple amides. CV = Cell viability (% of control) mean ± SD at 25 μM; ClogP = calculated partitioning; HBD = hydrogen bond donor at pH = 7; HBA = hydrogen bond acceptor at pH = 7; logBB = calculated blood-brain partition; MP = Molecular polarizability(Å^3^); PSA = Polar surface area (Å^2^); MPA = Minimal projection area (Å^2^); LogS = Aqueous solubility (mg/ml); MPO = Central nervous system multiparameter optimization (CNS MPO).

**Figure 4:**
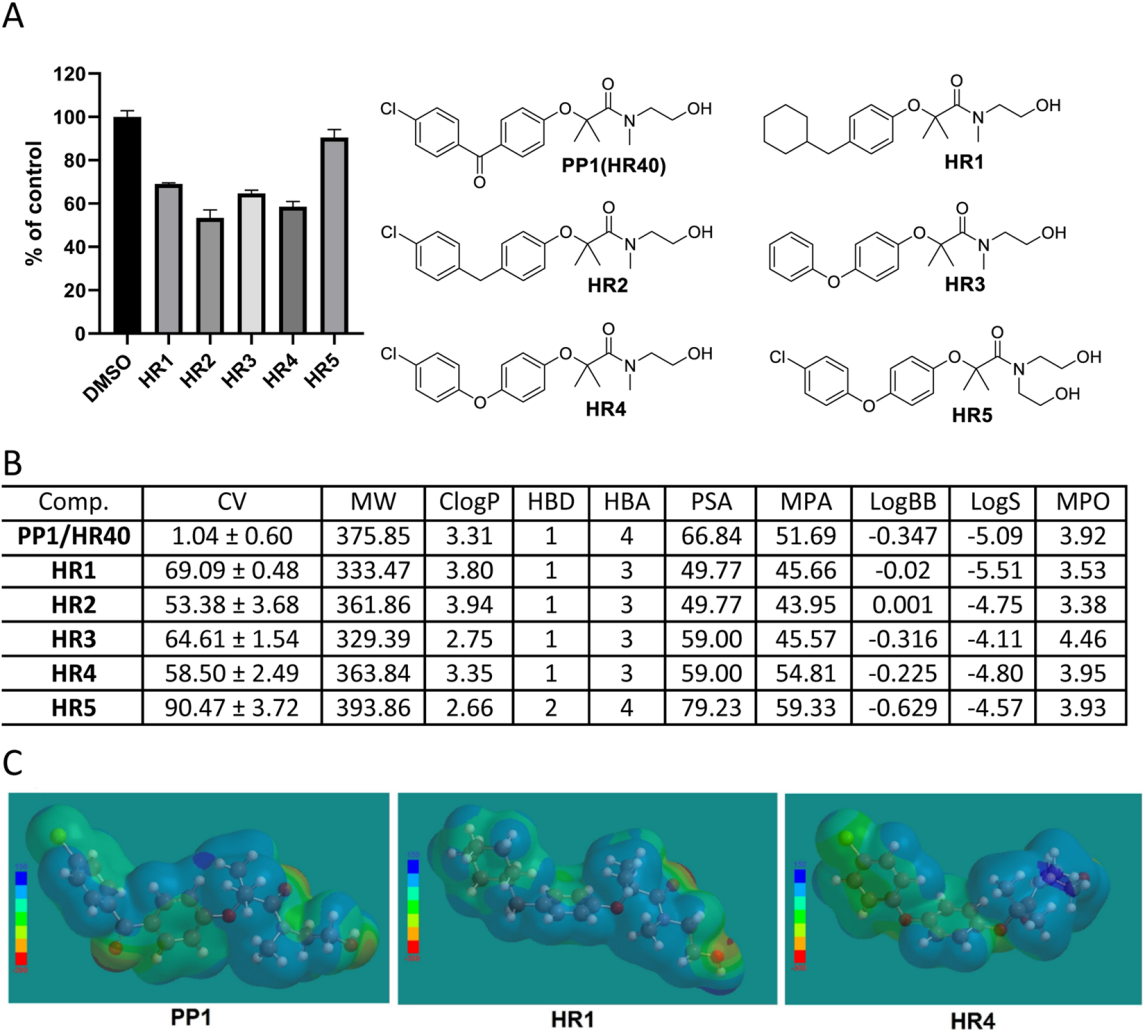
The BPA derivatives with methylene and oxygen in region B. Panel (A) Cell viability (MTT assay) following exposure to the indicated derivatives of FF (25 μM, for 72 hrs). Panel (B) CV = Cell viability (% of control) mean ± SD at 25 μM; ClogP = calculated partitioning; HBD = hydrogen bond donor at pH = 7; HBA = hydrogen bond acceptor at pH = 7; ClogBB = calculated blood-brain partition; PSA = Polar surface area (Å^2^); MPA = Minimal projection area (Å^2^); LogS = Aqueous solubility (mg/ml); MPO = Central nervous system multiparameter optimization (CNS MPO). Panel (C) Electrostatic potential map for PP1, HR1, and HR4 generated by semi-empirical method PM3 as implemented in Spartan ’18 version 1.1.0.

**Figure 5:**
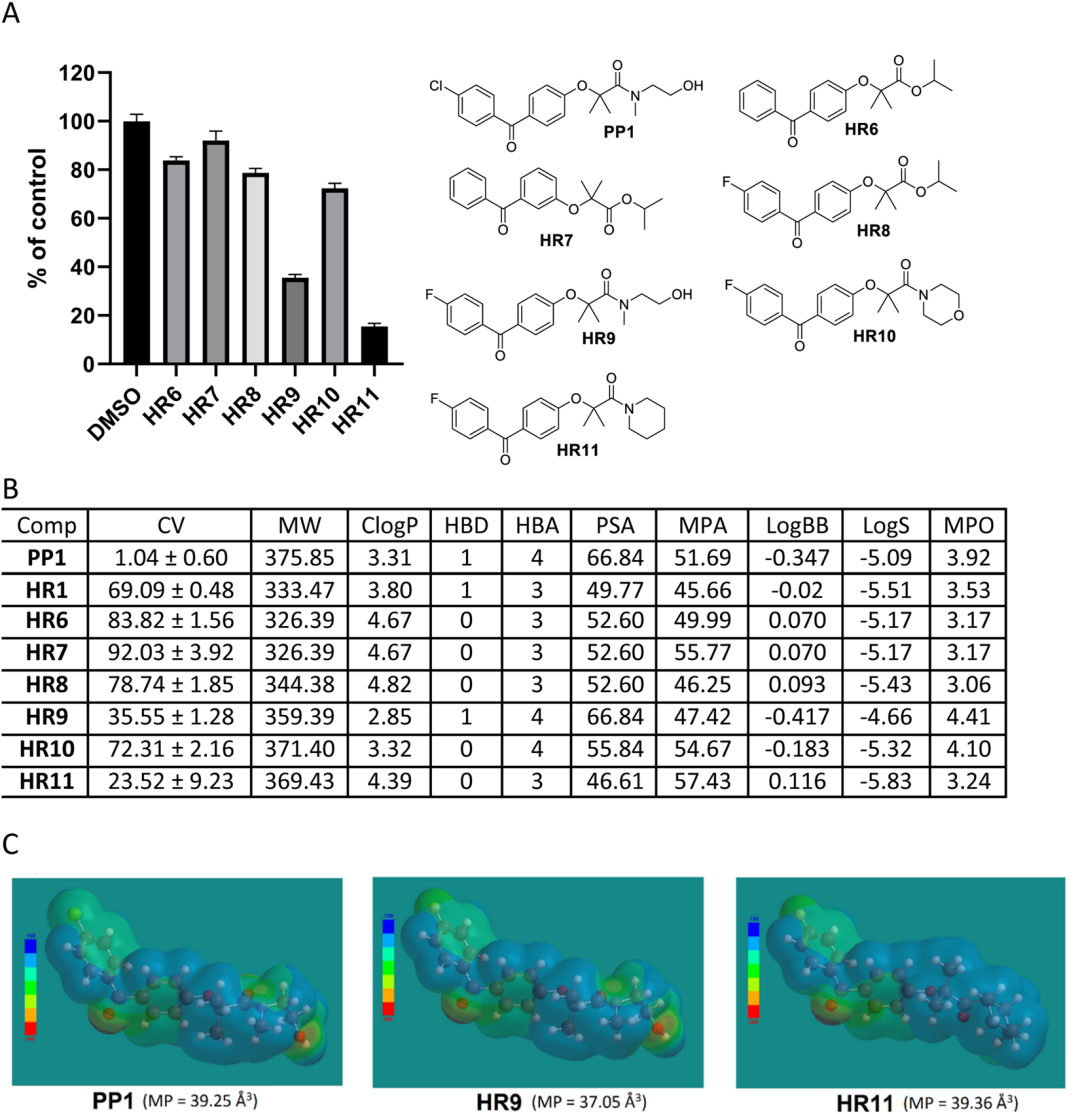
Fluoro- vs Chloro-benzylphenoxyacetamide. Panel (A) Cell viability (MTT assay) following exposure to modified variants of **PP1** in which chlorine atom was replaced (25 μM, for 72 hrs). Panel (B) CV = Cell viability (% of control) mean ± SD at 25 μM; ClogP = calculated partitioning; HBD = hydrogen bond donor at pH = 7; HBA = hydrogen bond acceptor at pH = 7; ClogBB = calculated blood-brain partition; PSA = Polar surface area (Å^2^); MPA = Minimal projection area (Å^2^); LogS = Aqueous solubility (mg/ml); MPO = Central nervous system multiparameter optimization (CNS MPO). Panel (C) Comparison of electrostatic potential map of our fluoro compounds with electrostatic potential map of **PP1**.

**Figure 6:**
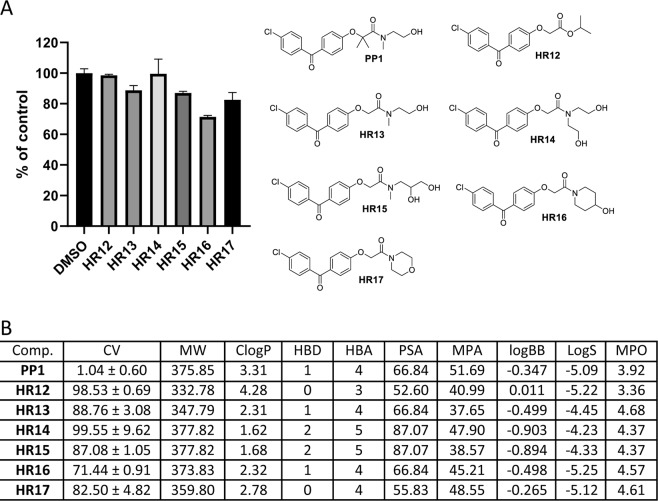
Drug candidates with unsubstituted alpha position of BPA. Panel (A) Cell viability (MTT assay) following exposure to modified variants of **PP1** with unsubstituted alpha position of BPA (25 μM, for 72 hrs). Panel (B) CV = Cell viability (% of control) mean ± SD at 25 μM; ClogP = calculated partitioning; HBD = hydrogen bond donor at pH = 7; HBA = hydrogen bond acceptor at pH = 7; ClogBB = calculated blood-brain partition; PSA = Polar surface area (Å^2^); MPA = Minimal projection area (Å^2^); LogS = Aqueous solubility (mg/ml); MPO = Central nervous system multiparameter optimization (CNS MPO).

**Figure 7:**
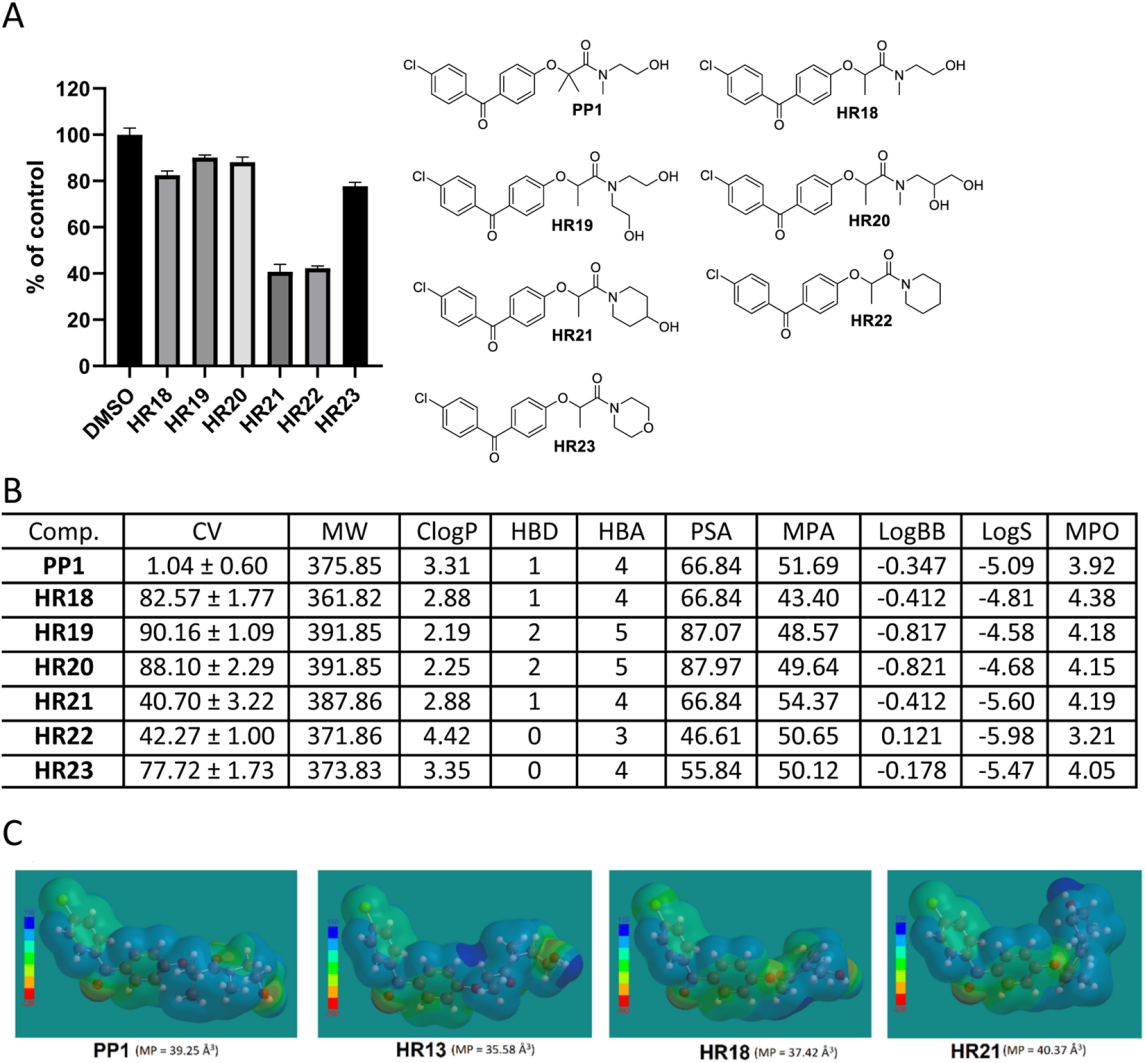
Drug candidates with alpha monomethylated BPA. Panel (A) Cell viability (MTT assay) following exposure to modified variants of PP1 with alpha monomethylated BPA (25 μM, for 72 hrs). Panel (B) CV = Cell viability (% of control) mean ± SD at 25 μM; ClogP = calculated partitioning; HBD = hydrogen bond donor at pH = 7; HBA = hydrogen bond acceptor at pH = 7; ClogBB = calculated blood-brain partition; PSA = Polar surface area (Å^2^); MPA = Minimal projection area (Å^2^); LogS = Aqueous solubility (mg/ml); MPO = Central nervous system multiparameter optimization (CNS MPO). Panel (C) Comparison of the electrostatic potential maps di-, mono- and non-methylated drug candidate (HR13, HR18 and HR21) with **PP1**.

**Figure 8:**
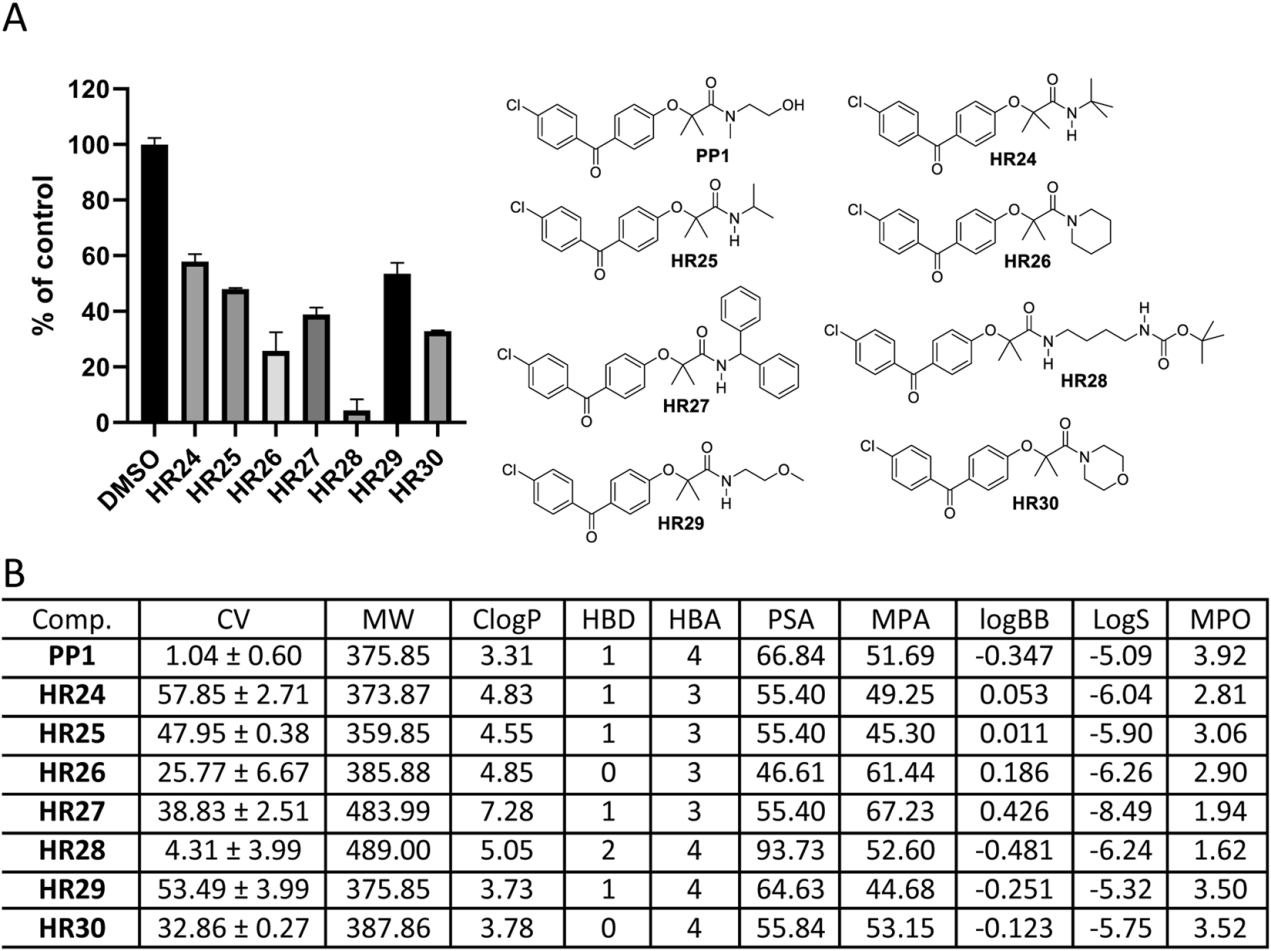
Drug candidates with a pH neutral amide moiety. Panel (A) Cell viability (MTT assay) following exposure to modified variants of **PP1** with pH neutral amide moiety (25 μM, for 72 hrs). Panel (B) CV = Cell viability (% of control) mean ± SD at 25 μM; ClogP = calculated partitioning; HBD = hydrogen bond donor at pH = 7; HBA = hydrogen bond acceptor at pH = 7; ClogBB = calculated blood-brain partition; PSA = Polar surface area (Å^2^); MPA = Minimal projection area (Å^2^); LogS = Aqueous solubility (mg/ml); MPO = Central nervous system multiparameter optimization (CNS MPO). **H28** activities at 10 μM (38.31 ± 3.50), 5 μM (65.90 ± 2.82), and 1 μM (92.75 ± 3.59).

**Figure 9:**
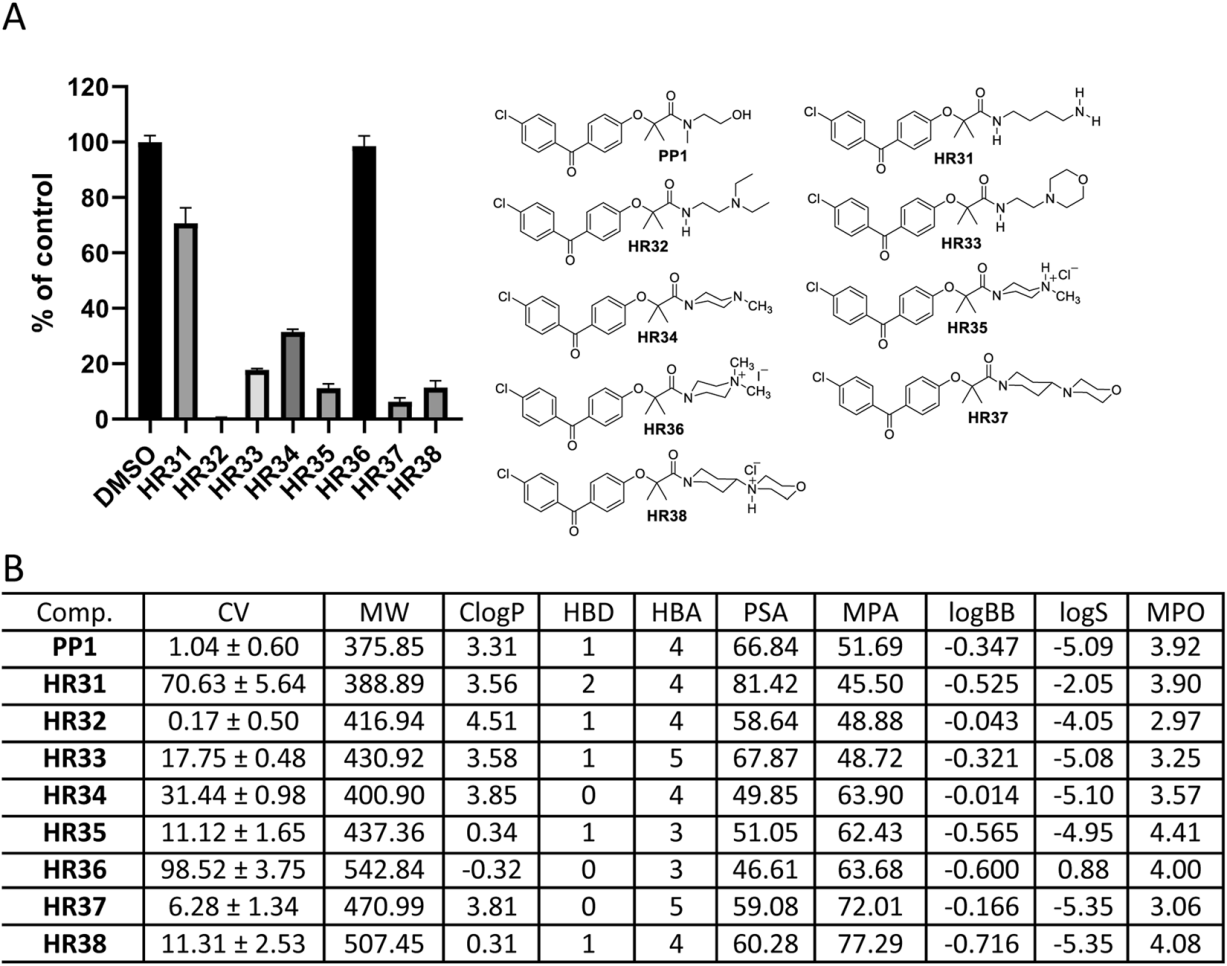
Drug candidates with basic amide moiety - protonated and alkylated. Panel (A) Cell viability (MTT assay) following exposure to modified variants of **PP1** with basic amide moiety (25 μM, for 72 hrs). Panel (B) CV = Cell viability (% of control) mean ± SD at 25 μM; ClogP = calculated partitioning; HBD = hydrogen bond donor at pH = 7; HBA = hydrogen bond acceptor at pH = 7; logBB = calculated blood-brain partition; PSA = Polar surface area (Å^2^); MPA = Minimal projection area (Å^2^); LogS = Aqueous solubility (mg/ml); MPO = Central nervous system multiparameter optimization (CNS MPO). **H32** CV at 10 μM (41.49 ± 7.94), CV at 5 μM (77.76 ± 7.24), and CV at 1 μM (96.80 ± 6.51). **H35** CV at 10 μM (56.26 ± 0.59), CV at 5 μM (79.34 ± 1.70), and CV at 1 μM (93.64 ± 2.08). **H37** CV at 10 μM (47.02 ± 1.23), CV at 5 μM (75.02 ± 1.42), and CV at 1 μM (109.20 ± 5.73). **H38** CV at 10 μM (52.00 ± 1.86), CV at 5 μM (74.89 ± 1.79), and CV 1 μM (97.97 ± 11.41).

**Figure 10:**
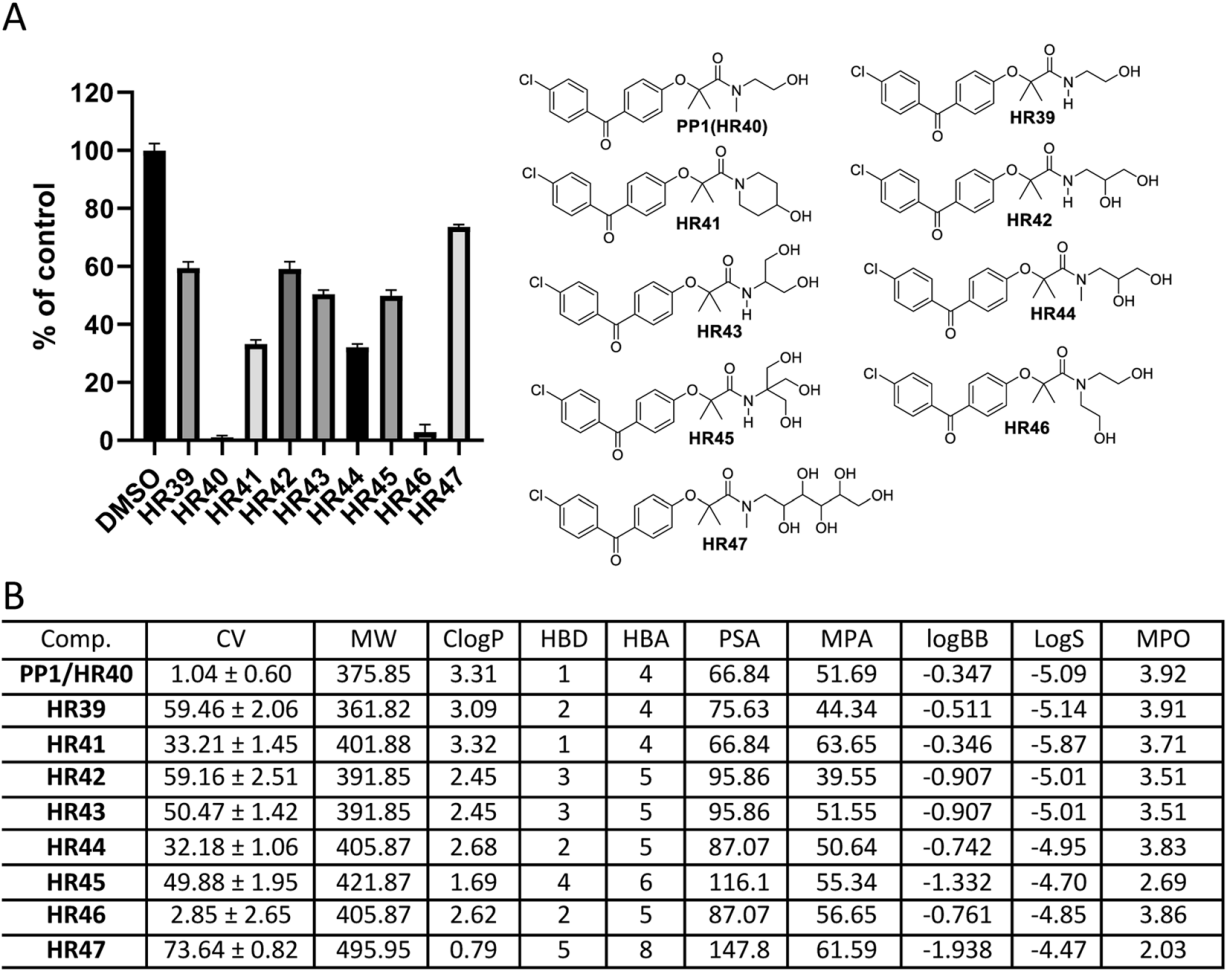
Drug candidates with one or several hydroxy groups in the amide moiety. Panel (A) Cell viability (MTT assay) following exposure to modified variants of **PP1** with one or several hydroxy groups in the amide moiety (25 μM, for 72 hrs). Panel (B) CV = Cell viability (% of control) mean ± SD at 25 μM; ClogP = calculated partitioning; HBD = hydrogen bond donor at pH = 7; HBA = hydrogen bond acceptor at pH = 7; logBB = calculated blood-brain partition; PSA = Polar surface area (Å^2^); MPA = Minimal projection area (Å^2^); LogS = Aqueous solubility (mg/ml); MPO = Central nervous system multiparameter optimization (CNS MPO). **PP1** CV at 10 μM (43.67 ± 1.88), CV at 5 μM (70.03 ± 2.04), and CV at 1 μM (98.36 ± 1.61). **H46** CV at 10 μM (51.97 ± 10.27), CV at 5 μM (86.15 ± 8.50), and CV at 1 μM (103.00 ± 2.98).

One fundamental challenge for the design of CNS penetrant drugs is the need to cross the blood-brain barrier (BBB). But, BBB-permeable compounds form a very small subset of oral drugs currently in existence, and experimental models for testing BBB penetration is quite complex. Therefore, an independent indicator of the BBB penetration was needed for the initial screening and selection of a large number of compounds (**BPA** variants) to evaluate their potential for reaching the intracranial tumor site at therapeutically relevant concentrations. Prior to the preparation of all **BPA** variant compounds in this study, we performed extensive molecular modeling to describe their physicochemical properties. A cell viability (CV) assay was then performed using the LN229 human glioblastoma cell line, and the cells were treated with BPA variants at 25 μM for 72 hours. The results of this computational characterization and cell viability testing are outlined in Figs. 3–10.

One currently accepted way to define physicochemical properties is by using a weighted scoring approach, known as the Central Nervous System – Multiparameter Optimization (CNS-MPO)^51–53^. The CNS-MPO algorithm uses a weighted scoring function that assesses 6 key physicochemical properties (clogP, clogD, MW, TPSA, HBD, and pKa) that indicate relative BBB penetration. The CNS-MPO scale is between 0 and 6.0, with scores ≥4.0 widely used as a cut-off to select compounds for hit CNS therapeutic drug discovery programs^53^. The validation of this approach utilizes a library of 616 compounds to evaluate the experimental distribution of the computed parameters incorporated into CNS-MPO scores^52,53^. It was found that CNS-MPO scores of 1–2 (0%), 2–3 (11.6%), 3–4 (40.8%), 4–5 (53.8%) and 5–6 (81.1%) correlate with the increased probability (%) of drugs to be found in the brain^54^.

In addition, parameters that are routinely used for a Quantitative Structure Activity Relationship (QSAR) study are molecular polarizability (MP), minimal molecular projection area (MPA), and water solubility (LogS). These parameters are not incorporated into the CNS-MPO score, however they are also considered as factors that affect BBB penetration. Therefore, in the associated figures below, these additional parameters were defined and calculated for each **BPA-**derived compound analyzed in this study. Comparing these values is a valuable tool for understanding how different substituents may change polarization- and dispersion-type interactions with the active sites of their interacting proteins^55^.

Molecular Polarizability (MP) is a response of electron distribution to an externally-applied static electrical field. It was postulated that an MP between 30–40 is optimal for a molecule to bind to a biotarget^56^. The minimal projected area (MPA) is also very important for drug transport and ultimately for drug activity. For instance, in recent studies by Cha, Müller, and Pos, a distinct phenotypical pattern of drug recognition and transport for the G616N variant was reported, indicating that drug substrates with MPA over 70 Å^2^ are less well transported than the smaller substrates^57,58^. Finally, water solubility (LogS) of −4.5 and greater are indicators of acceptable water solubility^59,60^ of the studied compounds.

The BBB permeation propensity of all studied compounds is also indicated by the decimal logarithm of brain to-plasma concentration ratio (logBB) value, which is derived from the modified Clark’s equation: logBB = 0.152 ClogP -0.0148PSA + 0.139 30. LogBB was also calculated for the compounds in this study, and is listed in the corresponding tables (Figs. 3–10. It has been shown that chemical compounds with logBB > 0.3 readily cross the BBB, while those with logBB < −1.0 are poorly distributed to the brain^61^. Finally, the rate of passive diffusion is inversely proportionate to the square root of molecular size (Graham’s law^62^), which is also included in our compound analysis.

Surprisingly, simple amide derivatives of **FF**, such as **AA**, **MAA**, and **DMA** (Fig. 3), have not been previously synthesized. But, in our quest for a better anti-glioblastoma drug, we began with preparation of these three small compounds, and an evaluation of their anti-glioblastoma activity as well as their potential ability to cross the BBB. Computed physicochemical parameters for **FF**, **FFA**, **MA**, **DMA** and **AA** and their anti-glioblastoma activity are presented in Fig. 3B. Each of the three amides showed better anti-tumor activity than **FF**. In fact, the simple amide **AA** is twice as potent, and the dimethyl amide **DMA** almost four times more potent, as **FF** at 25 µM (Fig. 3A). Computed CNS-MPO scores for the three amides are almost identical, around 3.5, suggesting that the likelihood of these compounds penetrating the BBB is around 40%. All estimated MPA for these molecules are between 35 and 50 Å2 so, based on their size alone they should be capable of crossing the cell membrane^57,63^. Therefore, MPA results presented in Fig. 3 confirm our postulate that the simple amides of FFA are viable structural motifs for exploring and possibly improving the anti-glioblastoma activity of FF.

Since our previously reported drug candidate **PP1** (**HR40**) has a very potent activity against glioblastoma tumor cells^47^, we turned our attention to larger molecules based on this **BPA** structure. To evaluate the products of the following structural modifications, we used **PP1** as a standard of comparison. We proceeded, by investigating the importance of the second aromatic ring in **BPA**, the conjugation of the carbonyl group, and the presence of a chlorine atom. All computed parameters for resulting compounds (HR1-HR5) (Fig. 4) suggest that these compounds will have moderately desirable physical properties for BBB penetration. ClogPs values are in the middle of the desirable range 2.0 to 5.0, and MPO values are between 3.5 and 4.5, therefore both suggest a possibility for these compounds to accumulate in the CNS (Fig. 4B). However, according to the calculated LogBB for these compounds, a low brain penetration ability could be also expected. The cell viability data at 25 μM for these 5 compounds (HR1-HR5) showed moderate to low anti-glioblastoma activity (Fig. 4A). Replacing the 4-chlorobenzoyl moiety of **PP1** with a cyclohexylmethyl generates the new drug candidate **HR1**. However, this modification results in a decrease of anti-glioblastoma activity (Fig. 4A). This is a somewhat drastic structural change, resulting in conformational and noticeable structural electrostatic potential surface changes (Fig. 4C)^64^. Lipophilicity is higher and molecular polar surface area is substantially lower (from 66.84 for **PP1** to 49.77 for **HR1** (Fig. 4B). However, this modification results in a decrease of anti-glioblastoma activity (Fig. 4A,B). If the presence of a polar group in the middle of the molecule is important, then replacement of the carbonyl group of the benzophenone moiety of **PP1** with an ether group (oxygen atom) should produce a new drug candidate, **HR4**, with similar activity to **PP1**. However, HR4 has only moderate anti-glioblastoma activity (CV = 58.5%), and another candidate in this group, HR5, lost its anti-glioblastoma activity (CV = 90.47%) (Fig. 4A). These results indicate that the presence of a second aromatic ring, the carbonyl linker, and chlorine are all important for retaining anti-glioblastoma activity of **PP1**.

Additionally, it is also important to assess the significance of the halogen atoms on the **BPA** skeleton. It is well documented that substituting hydrogen with fluorine substantially changes molecular polarizability (MP) and lipophilicity, and increases the binding affinity to targeted proteins^65^. Also, halogen bonding is stronger between chloro-aryls and carbonyl compounds, than between corresponding fluoro-aryls. Therefore, the computed data, as well as, the cell viability data of modified variants of **PP1** in which the chlorine atom was replaced with fluorine (**HR8-HR11**) are presented in Fig. 5. Although, the ester of fluoro-**FF** (**HR8**) has virtually the same activity (CV) as **FF**, other esters (**HR6**, **HR7**) are virtually inactive at the same concentration (25 μM) (Fig. 5A). So, further exploration of the anti-glioblastoma activity for ester derivatives of **FF** is not necessary.

Fluoro-**PP1** (**HR9**) has a lower potency than **PP1**, and therefore, halogen bonding appears to be very important for anti-glioblastoma activity in BPAs (Fig. 5A). The order of activity for the tested amides presented in Fig. 5 is **PP1 (HR40)** > **HR11** > **HR9**. However, the computed physicochemical parameters do not include an indicator of the effect of halogen bonding, which therefore, underestimates the contribution of the chlorine bonding in **PP1**. In addition, electrostatic potential maps for **PP1** and **HR9** (Fig. 5C) are very similar, therefore they are expected to have similar anti-glioblastoma activity in terms of molecular potential. However molecular polarizability MP = 39.25 Å^3^ for **PP1** and MP = 37.05 Å^3^ for **HR9** are quite different indicating different binding capabilities and may explain why **HR9** is less active (CV = 35.55%) compared to **PP1** (CV = 1.04%). Surprisingly, the molecular polarizability of **HR11** (39.36 Å^3^) is quite close to that of **PP1** (Fig. 5C), indicating that **HR11** might have a stronger binding capability to the targeted molecule than **HR9**, which is reflected by a slightly higher biological activity of **HR11** (Fig. 5A,B). These data indicate that calculated physicochemical properties are not always in a full agreement with the predicted biological responses, and once the candidate compounds are preselected, they require further validation using relevant cell culture animal models.

The **BPA** structure was further modified in order to explore the importance of two methyl groups in the alpha position (Fig. 2A; Region C). For non-methylated (unsubstituted alpha position) phenoxyamides, the physicochemical parameters and biological activity results are presented first in Fig. 6. All computed compounds, here, have both acceptable computed polar surface area (PSA) and minimal projected area (MPA), which indicate that they can penetrate cellular membranes^52,53,57^. The estimated CNS-MPO for these amides (**HR13**-**HR17)** are all more desirable than for **PP1** (Fig. 6B). Just based on this data, one would expect these compounds to have at least comparable biological activity to, if not better than, **PP1**. However, they are almost all inactive at the tested 25 μM concentration (Fig. 6A,B). Therefore, it is obvious that the presence of at least one methyl group is crucial for retaining activity.

The computed physical properties for the prepared monomethylated amides (shown in Fig. 7) are closer to the estimated physical properties of **PP1**, in respect to potential for BBB penetration, but their cell toxicity at 25 μM is very low (Fig. 7A,B). It is now also clear that two methyl groups are essential for retaining anti-glioblastoma activity. Comparison of the electrostatic potential for di –, mono-, and non-methylated compounds (HR13, HR18, HR21) suggests that a large positive area in the molecule is lost by removing the methyl groups (Fig. 7C). But, when a larger non-polar amide moiety is introduced, such as in the creation of **HR21** (Fig. 7C), a new positive electrostatic potential surface is generated that partially offsets the nonexistence of the methyl group. This is also indicated by a computed molecular polarizability (MP) that is slightly higher than that of **PP1**, resulting in modest activity (CV = 40.7%) for both **HR21** and **HR22** compared to **PP1** (CV = 1.04%) (Fig. 7A,B). So, in spite of the slight offset of the electrostatic potential maps, it seems apparent that two methyl groups in the alpha position of **BPA** are essential for retaining anti-glioblastoma activity. Therefore, the structural configuration necessary for anti-glioblastoma activity is approximately equal to the **FF** derivative, **AA**, introduced in Fig. 3.

After establishing the importance of the 2-(4-chlorobenzoyl)phenoxy-2,2-dimethylacetamide (**AA**) structural skeleton for anti-glioblastoma activity, it is now essential to assess the nature of the amide moiety (Fig. 2A; region C). Three groups of **AA** variants were designed, prepared and tested. The first group of compounds contained an amide moiety with neutral pH (Fig. 8) in order to establish the size and structural branching on the amide part of the molecule. Again, the physicochemical parameters and biological activity data were compared to our good drug candidate **PP1**. The computed MPAs for **HR24-HR30** are acceptable (40–60 Å^2^) and suggest that based on this property alone all of these compounds should have good cell permeability. On the other hand, based on the estimated PSA and CNS-MPO data, **HR28** should be expected to have a lower probability of penetrating into the cell, and possibly lower accumulation in the CNS. This is in spite of the fact that **HR28** is very potent in eliminating glioblastoma cells *in vitro* (CV = 4.31%) (Fig. 8A,B).

Next, amides with a basic amide moiety are presented in Fig. 9. For these compounds, cell permeability is strongly associated with the pH of the media. In acidic media, they are protonated and very hydrophilic. Additionally, they can be easily alkylated, which in turn can modify their polarity. All cell testing was done at physiological pH (pH = 7.4); therefore, the basic drug candidates were not protonated. Amide **HR31** has two nitrogen atoms separated by four methylene groups. One nitrogen is part of an amide bond and other is part of a primary amine. This compound has a CV value similar to that of 25 μM **FF**^27^ (Fig. 9A,B). However, when the carbon chain is shortened between the two nitrogens by two carbon atoms and the primary amine is transferred to a tertiary amine with two ethyl groups, a new drug candidate, **HR32**, is formed, with the highest anti-glioblastoma activity, thus far (CV = 0.17), and is more potent than **PP1** (Fig. 9A). Almost all computed parameters suggest that **HR32** is a good candidate (Fig. 9B); however, it has a CNS-MPO estimated value that is relatively low (2.97), suggesting that it may have a low probability of accumulating in the CNS. But, additionally, both the computed logBB and logS for this compound suggest that this, in fact, would be a good drug candidate (Fig. 9B).

The other amides in this group (**HR33–HR38**) with the exception of **HR36**, have a good anti-glioblastoma activity, including the hydrochloric salt **HR35**, which in addition has a more promising CNS-MPO value (4.41). In fact, **HR35** is more potent than its free base **HR34** (Fig. 9A). One could argue that this is the result of better initial solubility of this hydrochloric salt. However, in the cell culture environment, **HR35** should be deprotonated and become the free base, **HR34**, that is less potent (Fig. 9A). The reverse is true in the case of **HR37** and its hydrochloride salt **HR38**. The free base, **HR37**, is more potent than its hydrochloric salt (**HR38**) (Fig. 9A).

For the alkylated drug candidates, such as methylated **HR34**, the activity is substantially diminished because they cannot be deprotonated. If one examines the computed parameters for **HR36**, it is apparent that the compound is very hydrophilic (ClogP = −0.31; logS = 0.88) and has a very low estimated BBB penetration (logBB = −0.6). Surprisingly the CNS-MPO of **HR36** is 4.0, which would suggest that this compound should be capable of accumulating in CNS, however it is completely inactive at 25 μM (CV = 98%) (Fig. 9A,B). In conclusion, the best candidate from this group of compounds is **HR32**, which is more potent than **PP1**. In addition, **HR35** and **HR37** are also good candidates mostly because of their relatively high CNS-MPO and low CV values. In contrast, **HR36**, which is permanently positively charged, completely loses its anti-glioblastoma activity.

Water solubility is a major obstacle in the proper administration of drug candidates^66^. One approach to increasing water solubility is to introduce hydroxy groups in the non-essential structural area of the compound. The amide moiety of new compounds, listed in Fig. 10, seems to be an appropriate location to place one to several hydroxy groups. This modification of **PP1** starts by replacing the N-methyl group with N-H to make **HR39**. Although CNS-MPO values are virtually identical for **PP1** and **HR39**, the latter has less desirable ClogP and LogBB values, indicating potentially lower cell penetration. The resulting cell viability agrees with these estimates because **HR39** is significantly less potent than **PP1** (Fig. 10A,B). This finding suggests that tertiary amides are required for high anti-glioblastoma activity.

Introducing rigidity to stabilize a desired drug conformation could result in increased drug potency^67^ in many instances. One of the ways to introduce molecular rigidity is by replacing a linear carbon skeleton with a cyclic carbon skeleton. This modification to **PP1** resulted in the new drug candidate **HR41**, which is unfortunately less potent than **PP1** (Fig. 10A).

Four drug candidates containing two hydroxy groups (**HR42**, **HR43**, **HR44** and **HR46**) are also presented in Fig. 10. Two of them (**HR42** and **HR43**) are structural isomers, but both are secondary amides, as is **HR39**. Interestingly, **HR42** and **HR43** activities (CV) are almost identical, indicating that the presence of the tertiary amide moiety is more important than the presence of the two hydroxy groups. Two tertiary amides with two hydroxy groups, **HR44** and **HR46**, are also structural isomers. They are both more potent than the secondary amide, **HR39**, but the tertiary amide **HR46**, with two CH_2_CH_2_OH moieties, is almost as potent as **PP1**. However, introducing three (**HR45**) or more (**HR57**) hydroxy groups slightly decreases the potency, possibly because the compounds are becoming substantially more hydrophilic, as is demonstrated by lower Clog, MPO, and logBB estimated values (Fig. 10B). In conclusion, from this group (**HR39-HR47**) the best candidate is **HR46** due to its low CV value and acceptable CNS-MPO values that are very similar to the prototype drug, **PP1** (**HR40**).

**Figure 11:**
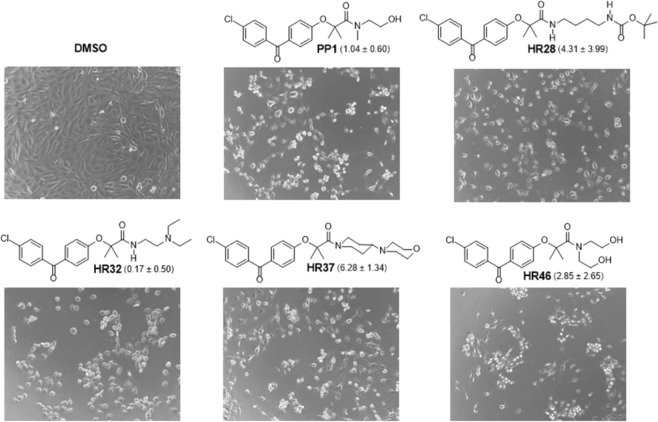
Low magnification phase contrast images of LN229 human glioblastoma cells treated with five selected drug candidates at 25 μM concentration (HR28, HR32, HR37,HR46 and **PP1** (HR40)). Control cells were treated with the equal volume of vehicle (DMSO). Images were taken 48 hours following the treatment. CV values for each compound are in parentheses.

In conclusion, we have identified four drug candidates, similar to **PP1** (HR40), that have strong *in vitro* anti-glioblastoma activity, but have physical properties that may contribute to improved brain tumor penetration (Fig. 11). The IC_50_ concentration of these compounds is around 10 μM, which is an acceptable therapeutic concentration for most clinically relevant anticancer drugs^68^. By exploring the computed structural and functional properties of benzylphenoxyacetamide (**BPA**), along with testing of cell viability, it was demonstrated that two methyl groups in the 2-position of **BPA** are important for retaining anti-glioblastoma activity. Substitution of the chloro- substituent in the 4-position of the benzophenone moiety, resulted in a significant loss of anticancer activity of the modified compound. The molecular rigidity between the two aromatic rings of the benzophenone moiety is essential because both methylene and oxygen replacement of the carbonyl group resulted in lost or diminished anti-tumoral activity. It was also demonstrated that tertiary amides are more potent than secondary amides, which, in turn, are more potent than primary amides. In tertiary amides, it is important to keep one substituent small (methyl), while the other group can be a hydroxy or nitrogen substituted alkyl. In addition to the evaluated chemical modifications in the BPA structure, there is still a high degree of flexibility for additional structural variations predominantly to the amide moiety (Fig. 2A; Region D). Exploring these additional structural variations, applying experimental and computational predictors of the BBB penetration, and testing drug efficacy in highly relevant brain tumor animal models, will produce more effective anti-glioblastoma drug candidates in the future.

## Methods

All starting materials were reagent grade and purchased from Sigma–Aldrich, ArkPharm, TCI America, and AbaChemScene. ^1^H-NMR spectra were recorded on Varian Mercury 300 and Varian Mercury 400 Plus instruments in CDCl_3_, DMSO-d_6_, using the solvent chemical shifts as an internal standard. Electrospray Mass Spectroscopy (EMS) was recorded on Waters LCT Premier XE (that’s a Tof MS) with an ESI source, scanning 100–2000 m/z with direct injections of 5 µl sample, using a 0.2 ml/min flow of acetonitrile. All molecular physical properties were calculated using Marvin Sketch software. All computed molecular descriptors were generated by ChemAxon MarvinSketch version 19.4. Electrostatic potential maps were calculated with PM3 semi-empirical method as implemented in Spartan ’18 v 1.1.0. NMR and MS spectra for all HR compounds generated in this study are included in Supplementary Materials.

### Cell culture and viability assays

Human glioblastoma LN-229 cells were maintained as semi-confluent monolayer culture in DMEM with 1 g/L glucose, sodium pyruvate, and L-glutamine (Corning) supplemented with 10% heat inactivated FBS (Gibco) and P/S (50 units/mL of penicillin and 50 µg/mL of streptomycin) at 37 °C in a 5% CO_2_ atmosphere. Prior to treatment, cells were plated in 96-well plates (BD Falcon) at initial density of 2 × 10^4^ cells/cm^2^. Stock solutions of the compounds were prepared in DMSO, diluted in cell culture medium and added to the cells in triplicate for every experimental condition 24 h after plating (final concentration 25 µM). For the vehicle control, DMSO was used at 0.5%. MTT assay^69^ (measuring cell metabolic activity) was performed after a 72 h incubation in the presence of the compounds, as previously described. Following 1 h incubation with MTT, formazan crystals were dissolved in 5 mM HCl in isopropanol and absorbance read at 540 nm. Data represent mean values expressed as a percentage of the vehicle control ± SD. Phase contrast images of treated cells were taken 48 hours following the treatment with a BZ-X800 fluorescence microscope (Keyence) equipped with a 20x objective.

### Method A

Preparation of isopropyl 2-(4-(4-chlorobenzoyl)phenoxy)acetate (**3n**). A water (10 ml) solution of sodium hydroxide (410 mg; 10.25 mmol), (4-chlorophenyl)(4-hydroxyphenyl)methanone (2.3 g; 0.1 mol) and benzene (100 ml) was refluxed for 10 minutes and water was azeotropically removed by using a Deen-Stark distillation apparatus followed by removal of benzene under reduced pressure. The resulting white powdery sodium phenoxide was mixed with dry isopropanol (100 ml) and isopropyl bromoacetate (1.9 g; 10.5 mmol). The resulting mixture was stirred with sonication for 1 hour and refluxed for 4 hours. Solvent was evaporated to solid residue and mixed with dichloromethane (100 ml) and water (100 ml). The water layer was discarded, and organic layer was washed with 5% sodium carbonate (3 × 50 ml) and dried over anhydrous sodium carbonate. After solvent evaporation, the resulting white residue was dried under vacuum to give a white solid product; isolated yield of 93% (3.1 g). ^1^H-NMR (400, DMSO-d_6_) δ 7.79 (2H, d, J = 9.2 Hz), 7.71 (2H, d, J = 8.8 Hz), 6.97 (2H, d, J = 8.8 Hz), 5.16 (1H, septet, J = 7.2 Hz), 4.67 (2H, s), and 1.28 (6H, d, J = 7. 2 Hz) ppm.

### Method B

Isopropyl 2-(3-benzoylphenoxy)-2-methylpropanoate (**4f**). An isopropanol (300 ml) suspension of sodium carbonate (21.2 g; 200 mmol), (3-hydroxyphenyl)(phenyl)methanone (4 g; 20 mmol) and isopropyl 2-bromo-2-isobutirate (4.2 g; 20 mmol) was refluxed with stirring for 3 days. After cooling to room temperature, the resulting white solid was separated by filtration washed with isopropanol (3 × 20 ml). Filtrates were evaporated and evaporated to an oily residue. This residue was mixed with water/chloroform (100 ml/200 ml). The water layer was discarded, and the chloroform layer was washed with 5% sodium hydroxide (3 × 50 ml), water (3 × 50 ml), and dried over anhydrous sodium carbonate. After filtration the chloroform was evaporated to oily residue, that standing at 5 °C overnight, gave a white solid in 92% (6 g) isolated yield. ^1^H-NMR(300 MHz, CDCl_3_) δ 7.77 (2H, d, J = 6.9 Hz), 7.68 (1H, t, J = 6.9 Hz), 7.48 (2H, d, J = 7.5 Hz), 7.5–7.3 (3H, m), 7.27 (1H, s), 7.07 (1H, d, J = 6.9 Hz), 5.06 (1H, septet, J = 6.3 Hz), 1.60 (6H, s), and 1.18 (6H, d, J = 6.9 Hz). ^13^C-NMR (200 MHz, CDCl_3_) δ 173.2, 155.4, 128.6, 137.5, 129.9, 129.0, 128.2, 127.5, 123.8, 123.1, 120.3, 79.4, 69.1, 25.3 and 21.5 ppm

### Method C

Acid preparation. Preparation of 2-methyl-2-(4-phenoxyphenoxy)propanoic acid (**1e**). A water (10 ml) solution of sodium hydroxide (0.42 mg;10.5 mmol), benzene (100 ml) and 4-phenoxyphenol (1.86 g; 10 mmol) was stirred at room temperature for one hour. Water was evaporated away by Dean-Stark distillation. The remaining benzene was removed under reduced pressure, and the resulting white powdery residue was mixed with isopropanol (200 ml) and isopropyl 2-bromo-2-methylpropanoate (2.1 g; 10 ml). The resulting mixture was stirred by sonication at 60 °C for two hours followed by refluxing overnight. After cooling to room temperature, 5% sodium hydroxide (100 ml) was added and the resulting mixture was refluxed for 2 hours. The solvent was evaporated to solid residue mixed with water (150 ml) and acidified with concentrated hydrochloric acid to pH ~3. The resulting white suspension was mixed with chloroform (100 ml) and the chloroform layer was separated, washed with water (3 × 50 ml), and then washed with 10% sodium carbonate (100 ml). The sodium carbonate layer was acidified with concentrated hydrochloric acid to pH ~ 3. The white precipitate was separated by filtration, washed with water and dried at room temperature under a vacuum to give a pure product [92% yield (2.5 g)]. ^1^H-NMR (400 MHz, CDCl_3_) δ 7.31 (2H, t, J = 7.6 Hz), 7.08 (1H, t, J = 7.6 Hz), 6.96 (2H, d, J = 7.6 Hz), 6.93 (4H, s), and 1.58 (6H, s) ppm.

### Method D

(nitrogen unsubstituted amides). Preparation of 2-(4-(4-chlorobenzoyl)phenoxy)-2-methylpropanamide (**AA**). A dichloromethane (10 ml) suspension of fenofibric acid (**FFA**, 318 mg; 1 mmol) and two drops of DMF was stirred at room temperature for 3 hours. The resulting clear dichloromethane solution was evaporated at 30 °C under reduced pressure. The remaining white solid residue was dissolved in tetrahydrofuran (20 ml) and mixed with aqueous ammonia (10 ml; 3.08 g; 0.17 mol) and stirred at room temperature for one hour. The resulting mixture was mixed with dichloromethane (50 ml) and water (50 ml). Organic solvent was separated, washed with water (3 × 50 ml), 5% sodium carbonate (50 ml), and dried over anhydrous sodium carbonate. The solvent was evaporated to give white solid product that was recrystallized from dichloromethane-cyclohexane to give pure product [93% yield (295 mg)]. ^1^H-NMR (400, DMSO-d_6_) δ 7.69 (4H, d + d, J_1_ = J_2_ = 8.4 Hz), 7.58 (2H, d, J = 8.4 Hz), 7.57 (1H, s), 7.32 (1H, s), 6.97 (2H, d, J = 8.4 Hz), and 1.50 (6H, s) ppm. ^13^C-NMR (400, DMSO-d_6_) δ 193.7, 175.4, 159.8,137.5, 136.7, 132.1, 131.6, 130.0, 129.0, 118.5, 80.9, and 25.4 ppm.

### Method E

(*N*-methyl amides). Preparation of 2-(4-(4-chlorobenzoyl)phenoxy)-N,2-dimethylpropanamide (**MA**). An aqueous solution (20 ml) of methylamine hydrochloride (675 mg; 10 mmol) and sodium carbonate (530 mg; 5 mmol) were mixed with tetrahydrofuran (20 ml) solution of acid chloride prepared, as explained above for fenofibric acid (**FFA**, 318 mg; 1 mmol), and stirred at room temperature for two hours. Dichloromethane was added (50 ml) and the organic layer washed with water (3 × 50 ml), 5% sodium carbonate (50 ml) and dried over anhydrous sodium carbonate. After evaporation, the resulting oily residue was mixed with cyclohexane (5 ml) and left at room temperature overnight. The white needle-like precipitates were separated by filtration and dried at 60 °C under vacuum to give pure product [90% yield (300 mg)]. ^1^H-NMR (400, DMSO-d_6_) δ 8.09 (1H, q, J = 4.8 Hz), 7.67 (2H, d, J = 8.8 Hz), 7.66 (2H, d, J = 8.8 Hz), 7.56 (2H, d, J = 8.8 Hz), 6.93 (2H, d, J = 8.8 Hz), 2.58 (3H, d, J = 4.8 Hz), and 1.47 (6H, s) ppm. ^13^C-NMR (400, DMSO-d_6_) δ 194.0, 174.0, 159.6, 137.6, 136.5, 132.2, 131.6, 130.2, 129.1, 118.9, 81.2, 30.7, and 25.4 ppm.

### Method F

(*N*,*N*-dimethyl amides). Preparation 2-(4-(4-chlorobenzoyl)phenoxy)-N,N,2-trimethylpropanamide (**DMA**). A mixture of 40% dimethylamine in water (10 ml; 88 mmol) and the acid chloride of fenofibric acid (**FFA**, 1 mmol) was stirred at room temperature for two hours. The reaction mixture was worked up as described above (Method E). Product was purified by crystallization from cyclohexane. The isolated yield was 89% (310 mg). ^1^H-NMR (400, DMSO-d_6_) δ 7.71 (2H, d, J = 8.8 Hz), 7.69 (2H, d, J = 8.8 Hz), 6.89 (2H, d, J = 8.8 Hz), 3.02 (3H, s), 2.82 (3H, s), and 1.59 ppm. ^13^C-NMR (400, DMSO-d_6_) δ 193.9, 171.5, 159.6, 137.5, 136.7, 132.6, 131.6, 130.1, 129.1, 116.8, 81.6, 37.3, and 25.9 ppm.

### Method G

(pH neutral nitrogen substituted phenoxyacetamides). Preparation of 2-(4-(4-chlorophenoxy)phenoxy)-*N*,*N*-bis(2-hydroxyethyl)-2-methylpropanamide (**HR5**). A dry dichloromethane (20 ml) solution of acid **1k** (306 mg; 1 mmol), oxalyl chloride (0.260 ml; 380 mg; 3 mmol) and two drops of DMF was stirred at room temperature for 4 hours. The solvent was evaporated, and residue was dissolved in dichloromethane and mixed with mixture of tetrahydrofuran (10 ml) in diethanolamine (160 mg; 1.5 mmol) and water (10 ml) with sodium carbonate (212 mg; 2 mmol). The resulting mixture was stirred at room temperature for 2 hours and the solvent was evaporated under reduced pressure. The solid residue was mixed with dichloromethane (100 ml) and water (100 ml). The water layer was discarded, and the organic layer was washed with 5% hydrochloric acid (3 × 50 ml), 5% sodium carbonate (3 × 50 ml), water (3 × 50 ml) and dried over anhydrous sodium carbonate. The solvent was evaporated under reduced pressure to give pure product yielding 84% (330 mg). ^1^H-NMR (300 MHz, CDCl_3_) δ 6.92 (6H, m), 6.82 (2H, d, J = 9.3 Hz), 3.92 (4H, m), 3.66 (2H, t, J = 5.8 Hz), 3.60 (2H, t, J = 5.8 Hz), and 1.65 (6H) ppm.

### Method H

(basic nitrogen substituted aryloxyacetamides). Preparation of 2-(4-(4-chlorobenzoyl)phenoxy)-N-(2-(diethylamino)ethyl)-2-methylpropanamide (**HR32**). A tetrahydrofuran (20 ml) mixture of **FFA** (318.75; 1 mmol), oxalyl chloride (0.26 ml; 3 mmol), and two drops of DMF was stirred at room temperature for 2 hours. The solvent was evaporated, and solid residue was mixed with dry tetrahydrofuran (20 ml) and cooled down to 5 °C. This solution was mixed at 5 °C with ice cold water solution (20 ml) of sodium carbonate (212 mg; 2 mol) and N,N-diethylethylenediamine (127 mg; 1.1 mmol). Resulting mixture was stirred at room temperature for two hours and evaporated to an oily residue. This residue was mixed with dichloromethane (100 ml) and 5% sodium carbonate (50 ml). Water layer was discarded, and organic layer was extensively washed with water (10 × 50 ml) and dried over anhydrous sodium carbonate. After solvent evaporation, the product was further purified by silica gel filtration with 5% triethylamine in ethyl acetate as the solvent. The isolated yield was 75% (317 mg). ^1^H-NMR (400 MHz, CDCl_3_) δ 7.73 (2H, d, J = 8.8 Hz), 7.69 (2H, d, J = 8.4 Hz), 7.45 (2H, d, J = 8.4 Hz), 7.730 (1H, broad s), 6.95 (2H, d, J = 8.8 Hz), 3.31 (2H, q, J = 6.0 Hz), 2.49 (2H, t, J = 6.0 Hz), 2.41 (4H, q, J = 7.2 Hz), 1.61 (6H, s), and 0.87 (6H, t, J = 7.2 Hz) ppm. ^13^C-NMR (300, CDCl_3_) δ 194.1, 173.8, 158.9, 138.5, 136.2, 131.8, 131.1, 128.5, 119.4, 119.0, 81.7, 51.3, 46.6, 36.8, 25.1, and 11.6 ppm.

### Method I

(catalytic hydrogenation of benzoylphenoxyacetamides) Preparation of 2-(4-(cyclohexylmethyl)phenoxy)-N-(2-hydroxyethyl)-N,2-dimethylpropanamide (**HR1**). An ethanol (50 ml) suspension of amide **HR40** (190 mg; 0.5 mmol) and 3% Pd/C (150 mg) was stirred at room temperature overnight under atmospheric hydrogen pressure. The catalyst was removed by filtration and the solvent was evaporated under reduced pressure to give a pure product with isolated yield of 96% (320 mg) isolated yield. ^1^H-NMR (400, CDCl_3_) δ 6.99 (2H, d, J = 8.4 Hz), 6.74 (2H, d, J = 8.4 Hz, 3.79 (2H, t, J = 5.2 Hz), 3.54 (2H, t, J = 5.2 Hz), 3.24 (3H, s), 3.21 (1H, m), 2.39 (2H, d, J = 7.6 Hz), 1,64 (4H, m), 1.62 (6H, s), 1.45 (2H, m), 1.17 (2H, m), and 0.98 (2H, m) ppm.

### Method J

(sodium borohydride – trifluoracetic acid reduction of benzolphenoxyacetamide carbonyl group). Preparation of 2-(4-(4-chlorobenzyl)phenoxy)-*N*-(2-hydroxyethyl)-*N*,2-dimethylpropanamide (**HR2**). A dichloromethane (30 ml) suspension of **HR40** (190 mg; 0.5 mmol) and fine grinded sodium borohydride (110 mg; 3 mmol) was kept at −5 °C for one hour. While stirring this suspension at −5 °C, trifluoracetic acid was added dropwise (15 ml), over a period of half an hour. The resulting suspension was then stirred at 0 °C for one hour and at room temperature for an additional two hours. The suspension was filtered, solid was discarded and dichloromethane filtrate was washed with 5% sodium carbonate (3 × 30 ml), dried over anhydrous sodium carbonate and evaporated to oily residue to give crude product. The product was purified by silica gel column chromatography with ethyl acetate – dichloromethane (7:3). The isolated yield was 72%. ^1^H-NMR (400, CDCl_3_) δ 7.23 (2H, d, *J* = 8.4 Hz), 7.08 (2H, d, *J* = 8.4 Hz), 7.01 (2H, d, *J* = 8.4 Hz), 6.76 (2H, d, *J* = 8.4 Hz), 3.85 (2H, s), 3.78 (2H, q, *J* = 4.8 Hz), 3.53 (2H, t, *J* = 4.8 Hz), 3.21 (3H, s), 2.73 (1H, t, *J* = 5.2 Hz), and 1.62 (6H, s) pppm.

### Method K

(hydrochloric salts of basic phenoxyacetamide). Preparation of 4‐{2‐[4‐(4‐chlorobenzoyl)phenoxy]‐2‐methylpropanoyl}6-8,17-22,27,46,50,52,53,55,63,67‐1‐methylpiperazin‐1‐ium chloride (**HR35**). A mixture of concentrated hydrochloric (3 ml) and **HR34** (200 mg; 0.5 mmol) was sonicated at room temperature for five minutes. The clear water solution was left under stream of nitrogen in hood for several hours to result in a white powder product. The white powder was dissolved in dichloromethane (10 ml) and dried over 4 Å molecular sieve overnight. The solvent was evaporated, and white residue was dried in vacuum to give 208 mg (95% yield). ^1^H-NMR (CDCl_3_) δ 13.20 (1H, broad s), 7.75 (2H, d. J = 8.8 Hz), 7.71 (2H, d, *J* = 8.0 Hz), 7.56 (2H, d, *J* = 8.0 Hz), 6.90 (2H, d, *J* = 8.8 Hz), 4.78 (2H, d, *J* = 13.2 Hz), 3.93 (1H, t, *J* = 12.8 Hz), 3.52 (1H, t, *J* = 13.2 Hz), 3.45 (1H, d, *J* = 12.4 Hz), 3.27 (1H, d, *J* = 10.0 Hz), 2.63 (3H, d, *J* = 4 Hz), 2.53 (1H, q, *J* = 10.0 Hz), 1.98 (1H, q, *J* = 9.6 Hz) and 1.71 (6H, s) ppm.

### Method L

(ammonium salts of basic phenoxyacetamides. Preparation of 4-{2-[4-(4-chlorobenzoyl)phenoxy]-2-methylpropanoyl}^15^-1,1-dimethylpiperazin-1-ium iodide (**HR36**). An acetone (20 ml) solution of **HR34** (100 mg; 0.25 mmol) and methyl Iodide (141 mg; 0.06 ml; 1 mmol) was left in the dark at room temperature for three days (~70 hours). During this procedure, a white solid precipitate was formed. This solid product was separated by filtration, washed with acetone (3 × 3 ml) and dried at 110 °C for two hours to give pure product in 96% yield (130 mg). ^1^H-NMR (DMSO-d_6_) δ 7.73 (2H, d, *J* = 8.8 Hz), 7.71 (2H, d, *J* = 8.8 Hz), 7.60 (2H, d, *J* = 7.6 Hz), 6.93 (2H, d, *J* = 8.4). 4.06 (2H, broad singlet), 3.86 (2H, broad singlet), 3.30 (2H, broad singlet), 3.25 (2H, broad singlet), 3.12 (6H, s) and 1.60 (3H, s) ppm. ^13^C-NMR (DMSO-d_6_) δ 193.7, 170.7, 159.1, 137.6, 136.5, 132.7,131.7, 130.6, 129.1, 117.7, 81.8, 60.7, 51.2, and 26.2 ppm.

## Supplementary information

Supplementary Dataset 1

## Data Availability

All data generated or analyzed during this study are included in this published article and the Supplementary Information Files. All HR compounds described in the paper are protected under the LSU provisional patent 29327-056-US2 and can be provided for testing upon request.

## References

[1] Di Carlo DT, Cagnazzo F, Benedetto N, Morganti R, Perrini P (2019). Multiple high-grade gliomas: epidemiology, management, and outcome. A systematic review and meta-analysis. Neurosurg Rev.

[2] Nakada M (2007). Molecular targets of glioma invasion. Cell Mol Life Sci.

[3] Paolillo, M., Boselli, C. & Schinelli, S. Glioblastoma under Siege: An Overview of Current Therapeutic Strategies. *Brain Sci* **8**, 10.3390/brainsci8010015 (2018). 10.3390/brainsci8010015 PMC5789346 29337870

[4] Terzis AJ, Niclou SP, Rajcevic U, Danzeisen C, Bjerkvig R (2006). Cell therapies for glioblastoma. Expert opinion on biological therapy.

[5] Davis ME (2016). Glioblastoma: Overview of Disease and Treatment. Clin J Oncol Nurs.

[6] Cen L (2013). Efficacy of protracted temozolomide dosing is limited in MGMT unmethylated GBM xenograft models. Neuro Oncol.

[7] Fu J (2009). Glioblastoma stem cells resistant to temozolomide-induced autophagy. Chinese medical journal.

[8] Munoz JL (2014). Temozolomide resistance in glioblastoma cells occurs partly through epidermal growth factor receptor-mediated induction of connexin 43. Cell death & disease.

[9] Ghosh D, Nandi S, Bhattacharjee S (2018). Combination therapy to checkmate Glioblastoma: clinical challenges and advances. Clin Transl Med.

[10] Ohgaki H, Kleihues P (2007). Genetic pathways to primary and secondary glioblastoma. The American journal of pathology.

[11] Desai K, Hubben A, Ahluwalia M (2019). The Role of Checkpoint Inhibitors in Glioblastoma. Target Oncol.

[12] Basu B, Ghosh MK (2019). Extracellular Vesicles in Glioma: From Diagnosis to Therapy. Bioessays.

[13] Fidoamore A (2017). Energy metabolism in glioblastoma stem cells: PPARalpha a metabolic adaptor to intratumoral microenvironment. Oncotarget.

[14] Kore RA (2018). Hypoxia-derived exosomes induce putative altered pathways in biosynthesis and ion regulatory channels in glioblastoma cells. Biochem Biophys Rep.

[15] Pirmoradi L, Seyfizadeh N, Ghavami S, Zeki AA, Shojaei S (2019). Targeting cholesterol metabolism in glioblastoma: a new therapeutic approach in cancer therapy. J Investig Med.

[16] Geng F (2016). Inhibition of SOAT1 Suppresses Glioblastoma Growth via Blocking SREBP-1-Mediated Lipogenesis. Clin Cancer Res.

[17] Drukala J (2010). ROS accumulation and IGF-IR inhibition contribute to fenofibrate/PPARalpha -mediated inhibition of glioma cell motility *in vitro*. Mol Cancer.

[18] Wybieralska E (2011). Fenofibrate attenuates contact-stimulated cell motility and gap junctional coupling in DU-145 human prostate cancer cell populations. Oncol Rep.

[19] Yamasaki D (2011). Fenofibrate suppresses growth of the human hepatocellular carcinoma cell via PPARalpha-independent mechanisms. Eur J Cell Biol.

[20] Wilk A (2012). Fenofibrate-induced nuclear translocation of FoxO3A triggers Bim-mediated apoptosis in glioblastoma cells *in vitro*. Cell Cycle.

[21] Koltai T (2015). Fenofibrate in cancer:mechanisms involved in anticancer activity. F1000Research.

[22] Grabacka MM (2016). Fenofibrate Induces Ketone Body Production in Melanoma and Glioblastoma. Cells. Frontiers in endocrinology.

[23] Araki H (2009). Analysis of PPARalpha-dependent and PPARalpha-independent transcript regulation following fenofibrate treatment of human endothelial cells. Angiogenesis.

[24] Bajaj, M. *et al*. Effects of peroxisome proliferator-activated receptor (PPAR)-alpha and PPAR-gamma agonists on glucose and lipid metabolism in patients with type 2 diabetes mellitus. *Diabetologia* (2007). 10.1007/s00125-007-0698-9 17520238

[25] Kraja, A. T. *et al*. Fenofibrate and metabolic syndrome. *Endocr Metab Immune Disord Drug Targets* **10**, 138–148, doi:EMID-DT-ABS-33 [pii] (2010). 10.2174/187153010791213047 PMC5278640 20406163

[26] Grabacka Maja, Pierzchalska Malgorzata, Dean Matthew, Reiss Krzysztof (2016). Regulation of Ketone Body Metabolism and the Role of PPARα. International Journal of Molecular Sciences.

[27] Wilk Anna, Wyczechowska Dorota, Zapata Adriana, Dean Matthew, Mullinax Jennifer, Marrero Luis, Parsons Christopher, Peruzzi Francesca, Culicchia Frank, Ochoa Augusto, Grabacka Maja, Reiss Krzysztof (2014). Molecular Mechanisms of Fenofibrate-Induced Metabolic Catastrophe and Glioblastoma Cell Death. Molecular and Cellular Biology.

[28] Clendening JW, Penn LZ (2012). Targeting tumor cell metabolism with statins. Oncogene.

[29] Egerod FL (2005). Biomarkers for early effects of carcinogenic dual-acting PPAR agonists in rat urinary bladder urothelium *in vivo*. Biomarkers.

[30] Goard CA (2010). Differential interactions between statins and P-glycoprotein: implications for exploiting statins as anticancer agents. Int J Cancer.

[31] Grabacka M, Plonka PM, Urbanska K, Reiss K (2006). Peroxisome proliferator-activated receptor alpha activation decreases metastatic potential of melanoma cells *in vitro* via down-regulation of Akt. Clin Cancer Res.

[32] Grabacka M, Reiss K (2008). Anticancer Properties of PPARalpha-Effects on Cellular Metabolism and Inflammation. PPAR Res.

[33] Panigrahy D (2008). PPARalpha agonist fenofibrate suppresses tumor growth through direct and indirect angiogenesis inhibition. Proc Natl Acad Sci USA.

[34] Saidi SA, Holland CM, Charnock-Jones DS, Smith SK (2006). *In vitro* and *in vivo* effects of the PPAR-alpha agonists fenofibrate and retinoic acid in endometrial cancer. Mol Cancer.

[35] Gardette, V. *et al*. Ten-year all-cause mortality in presumably healthy subjects on lipid-lowering drugs (from the Prospective Epidemiological Study of Myocardial Infarction [PRIME] prospective cohort). *Am J Cardiol* **103**, 381–386, doi:S0002-9149(08)01709-8 [pii], 10.1016/j.amjcard.2008.09.092 (2009). 10.1016/j.amjcard.2008.09.092 19166693

[36] Grabacka M, Pierzchalska M, Reiss K (2013). Peroxisome Proliferator Activated Receptor alpha Ligands as Anticancer Drugs Targeting Mitochondrial Metabolism. Curr Pharm Biotechnol.

[37] Grabacka M (2004). Inhibition of melanoma metastases by fenofibrate. Arch Dermatol Res.

[38] Shigeto T, Yokoyama Y, Xin B, Mizunuma H (2007). Peroxisome proliferator-activated receptor alpha and gamma ligands inhibit the growth of human ovarian cancer. Oncol Rep.

[39] Urbanska K (2008). Activation of PPARalpha inhibits IGF-I-mediated growth and survival responses in medulloblastoma cell lines. Int J Cancer.

[40] Yokoyama Y, Xin B, Shigeto T, Mizunuma H (2011). Combination of ciglitazone, a peroxisome proliferator-activated receptor gamma ligand, and cisplatin enhances the inhibition of growth of human ovarian cancers. J Cancer Res Clin Oncol.

[41] Sterba J (2006). Combined biodifferentiating and antiangiogenic oral metronomic therapy is feasible and effective in relapsed solid tumors in children: single-center pilot study. Onkologie.

[42] Zapletalova D (2012). Metronomic chemotherapy with the COMBAT regimen in advanced pediatric malignancies: a multicenter experience. Oncology.

[43] Gamerdinger, M., Clement, A. B. & Behl, C. Cholesterol-like effects of selective COX inhibitors and fibrates on cellular membranes and amyloid-{beta} production. *Mol Pharmacol* (2007). 10.1124/mol.107.034009 17395689

[44] Nadanaciva, S., Dykens, J. A., Bernal, A., Capaldi, R. A. & Will, Y. Mitochondrial impairment by PPAR agonists and statins identified via immunocaptured OXPHOS complex activities and respiration. *Toxicol Appl Pharmacol* **223**, 277–287, doi:S0041-008X(07)00265-7 [pii], 10.1016/j.taap.2007.06.003 (2007). 10.1016/j.taap.2007.06.003 17658574

[45] Zungu, M., Felix, R. & Essop, M. F. Wy-14,643 and fenofibrate inhibit mitochondrial respiration in isolated rat cardiac mitochondria. *Mitochondrion* **6**, 315–322, doi:S1567-7249(06)00175-9 [pii], 10.1016/j.mito.2006.09.001 (2006). 10.1016/j.mito.2006.09.001 17046337

[46] Grabacka M (2015). Fenofibrate subcellular distribution as a rationale for the intracranial delivery through biodegradable carrier. Journal of physiology and pharmacology: an official journal of the Polish Physiological Society.

[47] Stalinska, J. *et al*. Chemically modified variants of fenofibrate with anti-glioblastoma potential. *Translational Oncology* (2019). 10.1016/j.tranon.2019.04.006 PMC6514324 31078963

[48] Eisenbraun EJ, Payne KW (1999). Dean-Stark apparatus modified for use with molecular sieves. Ind Eng Chem Res.

[49] Valeur E, Bradley M (2009). Amide bond formation: beyond the myth of coupling reagents. Chem Soc Rev.

[50] Rani, P., Pal, D., Hegde, R. R. & Hashim, S. R. Anticancer, Anti-Inflammatory, and Analgesic Activities of Synthesized 2-(Substituted phenoxy) Acetamide Derivatives. *Biomed Res Int*, doi:Artn 386473, 10.1155/2014/386473 (2014). 10.1155/2014/386473 PMC4150437 25197642

[51] Kim M (2019). Brain Distribution of a Panel of Epidermal Growth Factor Receptor Inhibitors Using Cassette Dosing in Wild-Type and Abcb1/Abcg2-Deficient Mice. Drug Metabolism and Disposition.

[52] Wager TT, Hou XJ, Verhoest PR, Villalobos A (2010). Moving beyond Rules: The Development of a Central Nervous System Multiparameter Optimization (CNS MPO) Approach To Enable Alignment of Druglike Properties. Acs Chem Neurosci.

[53] Wager TT, Hou XJ, Verhoest PR, Villalobos A (2016). Central Nervous System Multiparameter Optimization Desirability: Application in Drug Discovery. Acs Chem Neurosci.

[54] Rankovic Z (2015). CNS Drug Design: Balancing Physicochemical Properties for Optimal Brain Exposure. J Med Chem.

[55] Wang JM (2011). Development of Polarizable Models for Molecular Mechanical Calculations I: Parameterization of Atomic Polarizability. J Phys Chem B.

[56] Naef R (2015). A Generally Applicable Computer Algorithm Based on the Group Additivity Method for the Calculation of Seven Molecular Descriptors: Heat of Combustion, LogPO/W, LogS, Refractivity, Polarizability, Toxicity and LogBB of Organic Compounds; Scope and Limits of Applicability. Molecules.

[57] Cha HJ, Muller RT, Pos KM (2014). Switch-Loop Flexibility Affects Transport of Large Drugs by the Promiscuous AcrB Multidrug Efflux Transporter. Antimicrob Agents Ch.

[58] Ramaswamy VK, Vargiu AV, Malloci G, Dreier J, Ruggerone P (2017). Molecular Rationale behind the Differential Substrate Specificity of Bacterial RND Multi-Drug Transporters. Sci Rep.

[59] Caudana F, Ermondi G, Vallaro M, Shalaeva M, Caron G (2019). Permeability prediction for zwitterions via chromatographic indexes and classification into ‘certain’ and ‘uncertain’. Future Med Chem.

[60] Salvi A, Carrupt P, Mayer J, Testra B (1997). Esterase-Like Activity of Human Serum Albumin Toward Prodrug Esters of Nicotinic Acid. Drug Metabolism and Disposition.

[61] Vilar S, Chakrabarti M, Costanzi S (2010). Prediction of passive blood-brain partitioning: Straightforward and effective classification models based on in silico derived physicochemical descriptors. J Mol Graph Model.

[62] Piiper J, Worth H (1980). Value and limits of Graham’s law for prediction of diffusivities of gases in gas mixtures. Respir Physiol.

[63] Shinoda W (2016). Permeability across lipid membranes. Bba-Biomembranes.

[64] Kumar, A. & Zhang, K. Y. J. Advances in the Development of Shape Similarity Methods and Their Application in Drug Discovery. *Front Chem* **6**, doi:ARTN 315, 10.3389/fchem.2018.00315 (2018). 10.3389/fchem.2018.00315 PMC6068280 30090808

[65] Shah P, Westwell AD (2007). The role of fluorine in medicinal chemistry. J Enzym Inhib Med Ch.

[66] Williams HD (2013). Strategies to Address Low Drug Solubility in Discovery and Development. Pharmacol Rev.

[67] Lawson ADG, MacCoss M, Heer JP (2018). Importance of Rigidity in Designing Small Molecule Drugs To Tackle Protein-Protein Interactions (PPIs) through Stabilization of Desired Conformers. J Med Chem.

[68] Liston DR, Davis M (2017). Clinically Relevant Concentrations of Anticancer Drugs: A Guide for Nonclinical Studies. Clinical Cancer Research.

[69] Mosmann T (1983). Rapid colorimetric assay for cellular growth and survival: application to proliferation and cytotoxicity assays. J Immunol Methods.

