# Supplementary material for: Exploring anticancer activity of structurally modified benzylphenoxyacetamide (BPA); I: Synthesis strategies and computational analyses of substituted BPA variants with high anti-glioblastoma potential
Source: Sci Rep. 2019 Nov 19;9:17021. doi: 10.1038/s41598-019-53207-0 (PMC6864087; doi:10.1038/s41598-019-53207-0)
Supplement: Supplementary file 1 — Supplementary Dataset 1 [file 41598_2019_53207_MOESM1_ESM.pdf]

Exploring anticancer activity of structurally modified benzylphenoxyacetamide (BPA). I: Synthesis strategies and computational analyses of substituted BPA variants with high anti-glioblastoma potential.

Joanna Stalinska[c,d], Lisa Houser[a], Monika Rak[c,d], Susan Colley[c], Krzysztof Reiss[c*], and Branko S. Jursic[a,b*]

[a]Department of Chemistry, University of New Orleans, New Orleans, LA 70148, United States; [b]Stepharm llc., PO Box 24220, New Orleans, LA 70184, United States; [c]Neurological Cancer Research, Stanley S. Scott Cancer Center, Department of Medicine, LSU Health Sciences Center, New Orleans, LA 70112; [d]Department of Cell Biology, Faculty of Biochemistry, Biophysics and Biotechnology, Jagiellonian University, Cracow Poland.

Supplementary Materials

Ester and Acid Structures

NMR and MS

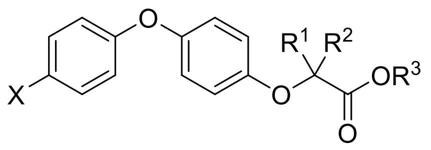

1a: X = R^1 = R^2 = H, R^3 = H
1b: X = R^1 = R^2 = H, R^3 = CH_2CH_3
1c: X = R^1 = H, R^2 = CH_3, R^3 = H
1d: X = H = R^1 = H, R^2 = CH_3, R^3 = CH_2CH_3
1e: X = H, R^1 = R^2 = CH_3, R^3 = H
1f: X = H, R^1 = R^2 = CH_3, R^3 = $CH(CH_3)_2$
1g: X = Cl, R^1 = R^2 = H, R^3 = H
1h: X = Cl, R^1 = R^2 = H, R^3 = CH_2CH_3
1i: X = Cl, R^1 = H, R^2 = CH_3, R^3 = H
1j: X = Cl, R^1 = H, R^2 = CH_3, R^3 = CH_2CH_3
1k: X = Cl, R^1 = R^2 = CH_3, R^3 = H
1l: X = Cl, R^1 = R^2 = CH_3, R^3 = $CH(CH_3)_2$

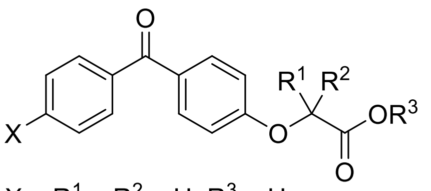

3a: X = R^1 = R^2 = H, R^3 = H
3b: X = R^1 = R^2 = H, R^3 = CH_2CH_3
3c: X = R^1 = H, R^2 = CH_3, R^3 = H
3d: X = H = R^1 = H, R^2 = CH_3, R^3 = CH_2CH_3
3e: X = H, R^1 = R^2 = CH_3, R^3 = H
3f: X = H, R^1 = R^2 = CH_3, R^3 = $CH(CH_3)_2$
3g: X = F, R^1 = R^2 = H, R^3 = H
3h: X = F, R^1 = R^2 = H, R^3 = CH_2CH_3
3i: X = F, R^1 = H, R^2 = CH_3, R^3 = H
3j: X = F, R^1 = H, R^2 = CH_3, R^3 = CH_2CH_3
3k: X = F, R^1 = R^2 = CH_3, R^3 = H
3l: X = F, R^1 = R^2 = CH_3, R^3 = $CH(CH_3)_2$
3m: X = Cl, R^1 = R^2 = H, R^3 = H
3n: X = Cl, R^1 = R^2 = H, R^3 = $CH(CH_3)_2$
3o: X = Cl, R^1 = H, R^2 = CH_3, R^3 = H
3p: X = Cl, R^1 = H, R^2 = CH_3, R^3 = CH_2CH_3
3r: X = Cl, R^1 = R^2 = CH_3, R^3 = H
3s: X = Cl, R^1 = R^2 = CH_3, R^3 = $CH(CH_3)_2$

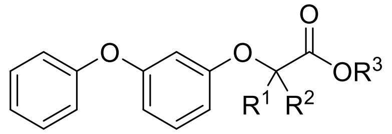

2a: R^1 = R^2 = R^3 = H
2b: R^1 = R^2 = H, R^3 = CH_2CH_3
2c: R^1 = H, R^2 = CH_3, R^3 = H
2d: R^1 = H, R^2 = CH_3, R^3 = CH_2CH_3
2e: R^1 = R^2 = CH_3, R^3 = H
2f: R^1 = R^2 = CH_3, R^3 = $CH(CH_3)_2$

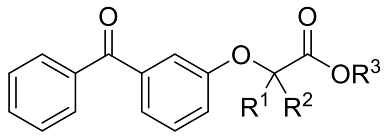

4a: R^1 = R^2 = R^3 = H
4b: R^1 = R^2 = H, R^3 = CH_2CH_3
4c: R^1 = CH_3, R^2 = H, R^3 = H
4d: R^1 = CH_3, R^2 = H, R^3 = $CH(CH_3)_2$
4e: R^1 = R^2 = CH_3, R^3 = H
4f: R^1 = R^2 = CH_3, R^3 = $CH(CH_3)_2$

Structures of synthesized ester and acids required for preparation of **BPA** derivatives

^{1}H-NMR ($DMSO-d_6$) Varian Mercury 400 Plus

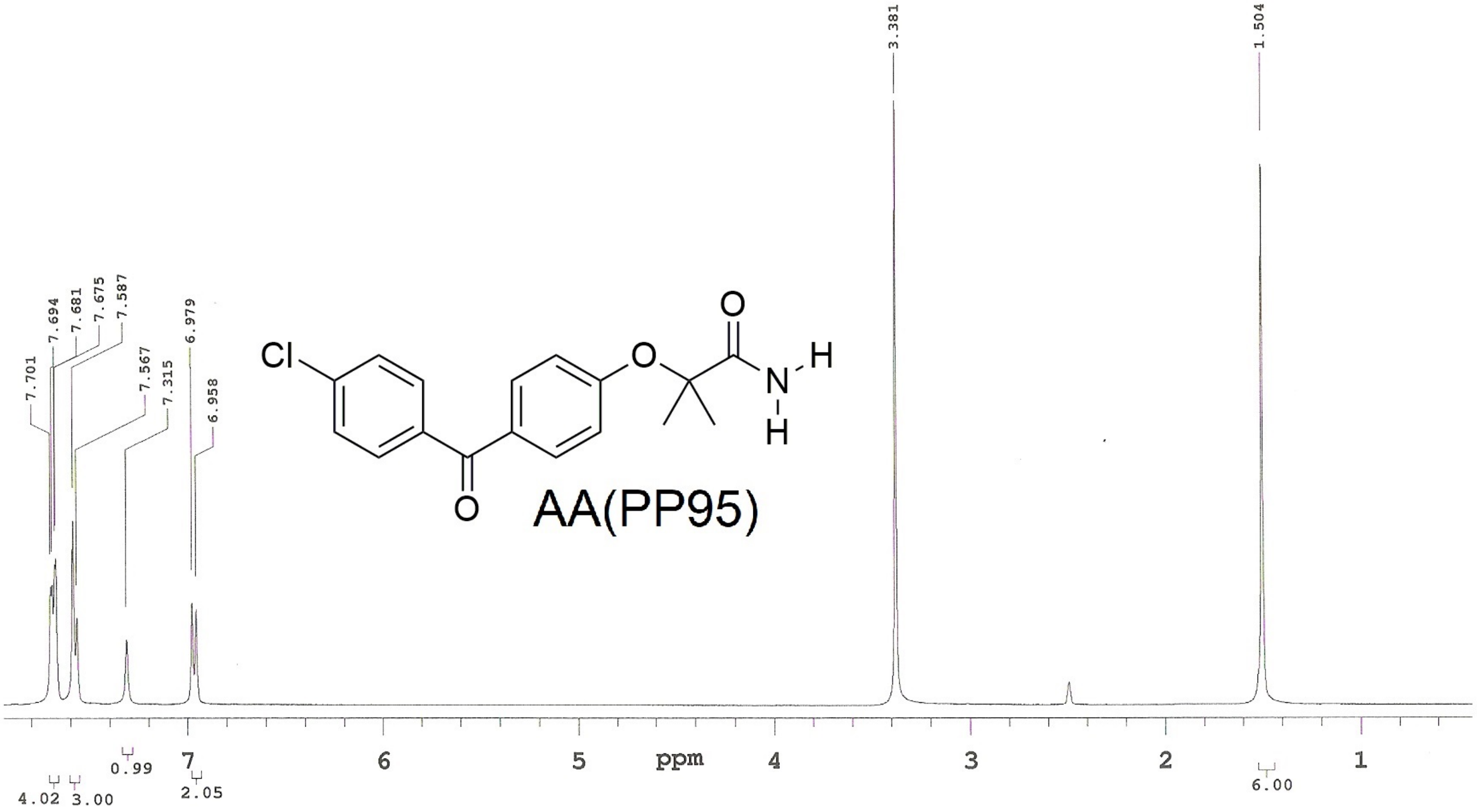

^{13}C-NMR (DMSO-d_6) Varian Mercury 400 Plus

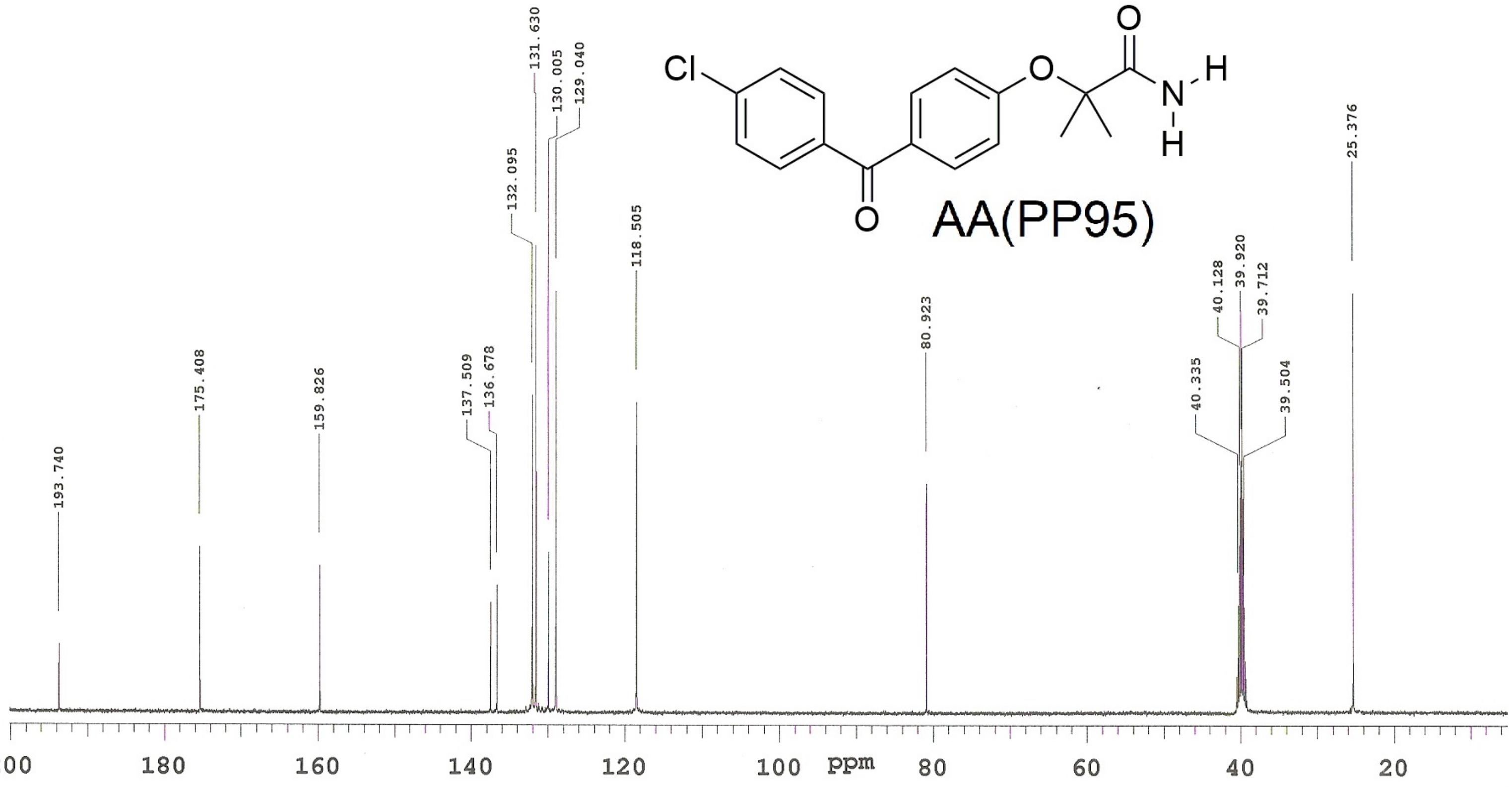

AA(PP95)

^{1}H-NMR (DMSO-d$_6$) Varian Mercury 400 Plus

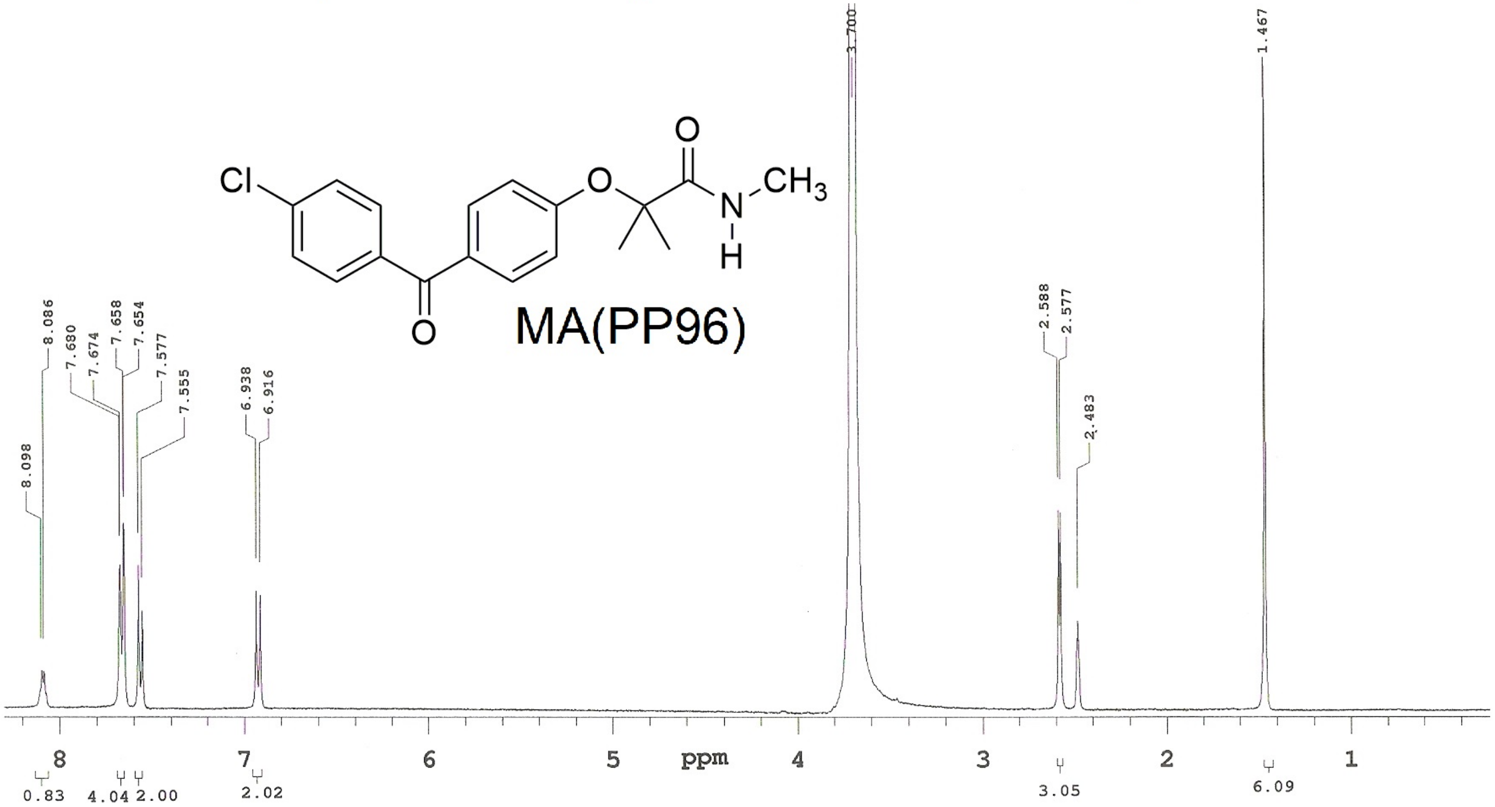

^{13}C-NMR (DMSO-d_6) Varian Mercury 400 Plus

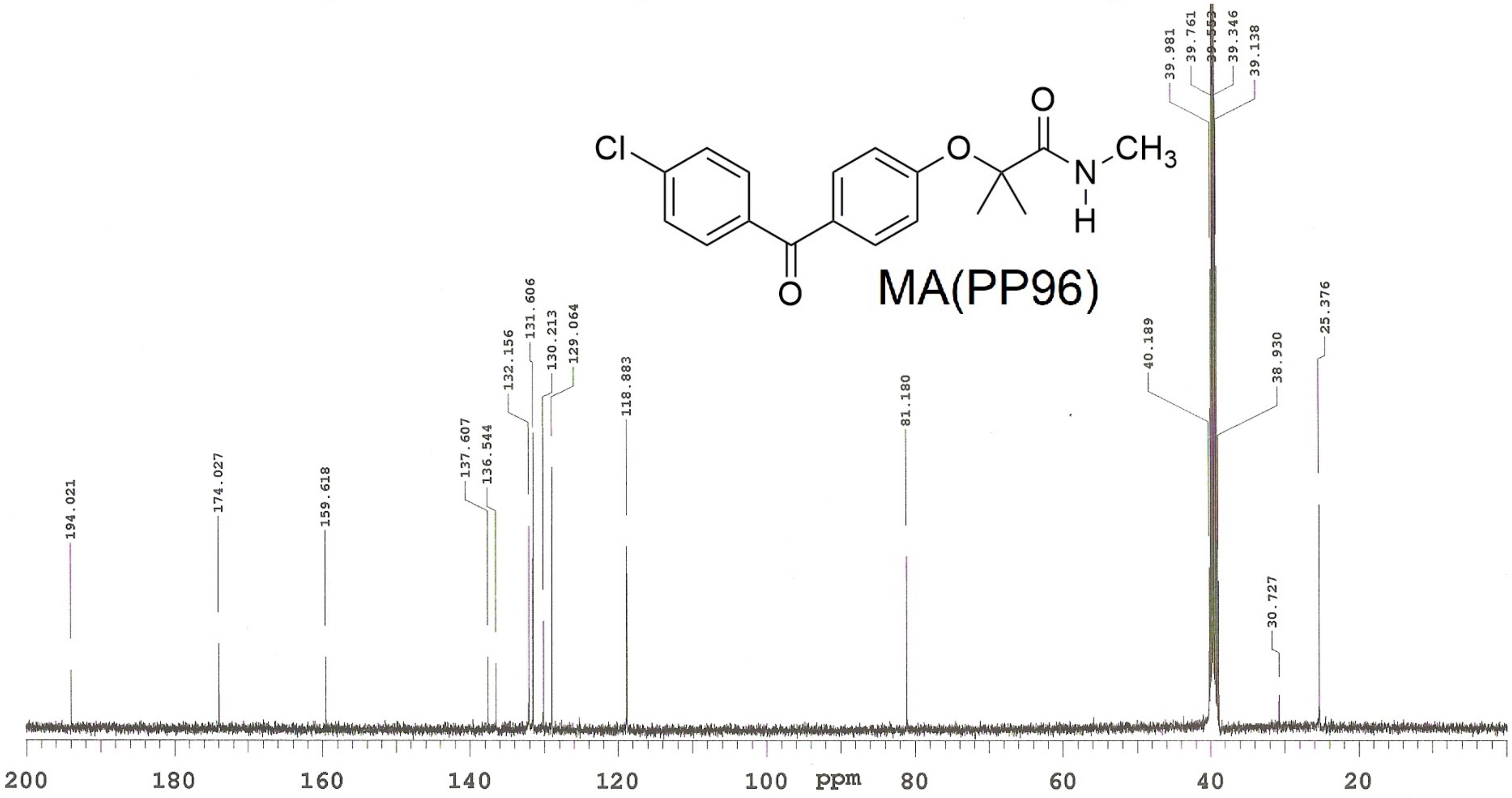

^{1}H-NMR (DMSO-d$_6$) Varian Mercury 400 Plus

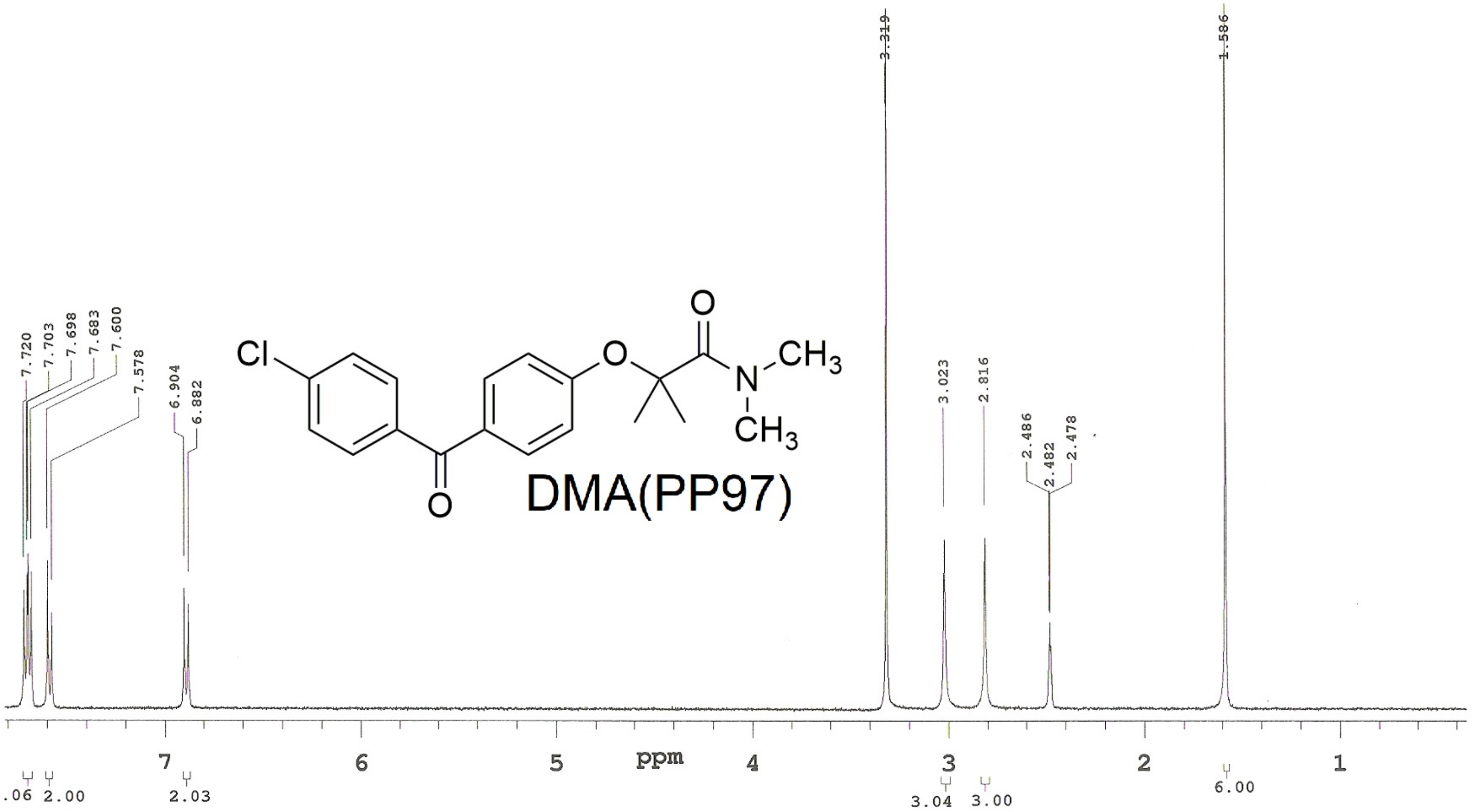

^{13}C-NMR (DMSO-d_6) Varian Mercury 400 Plus

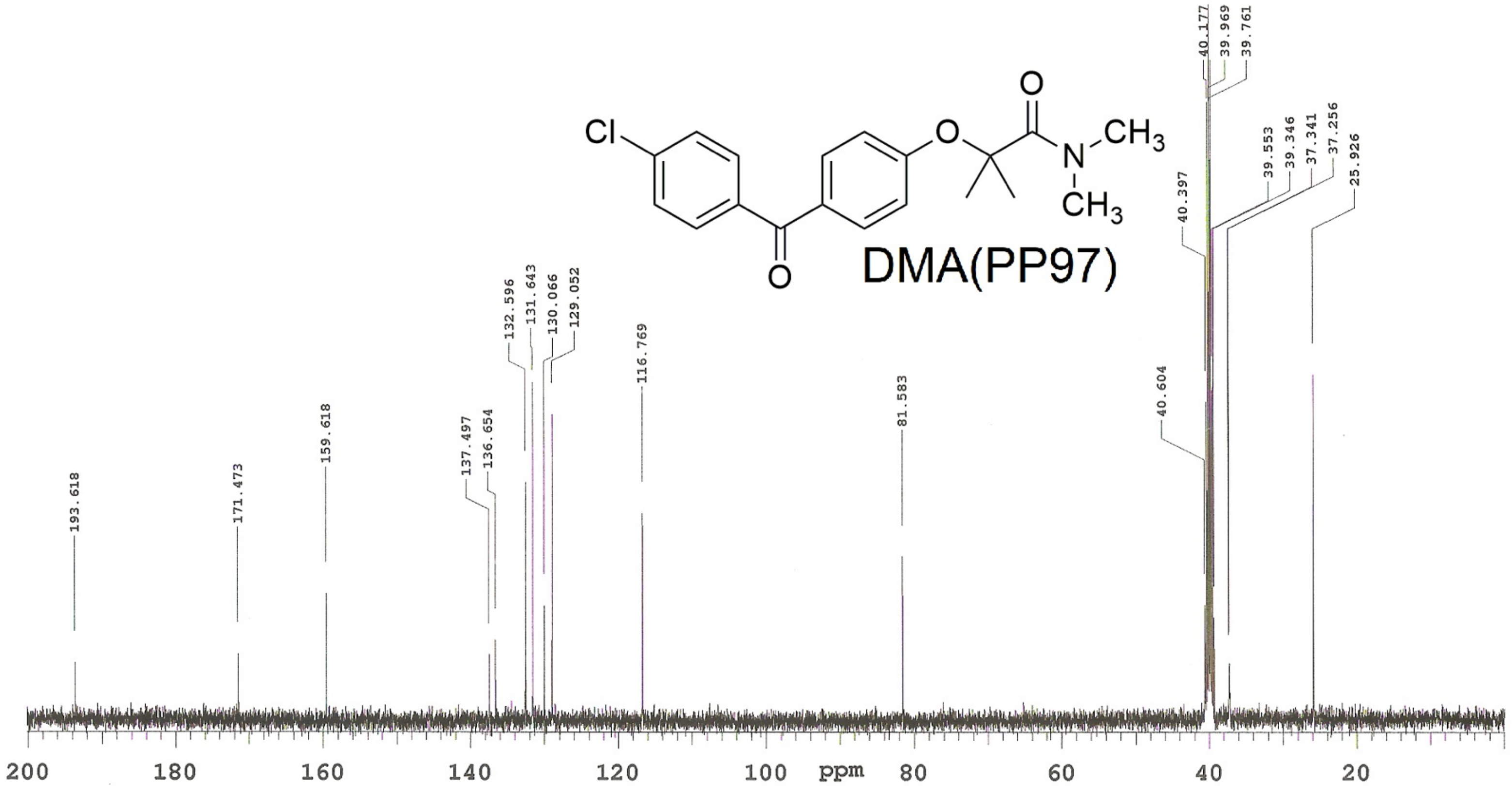

^{1}H-NMR ($CDCl_3$) Varian Mercury 400 Plus

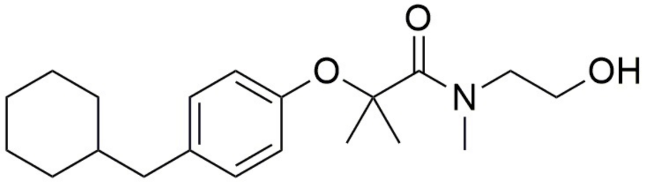

HR1(PP45)

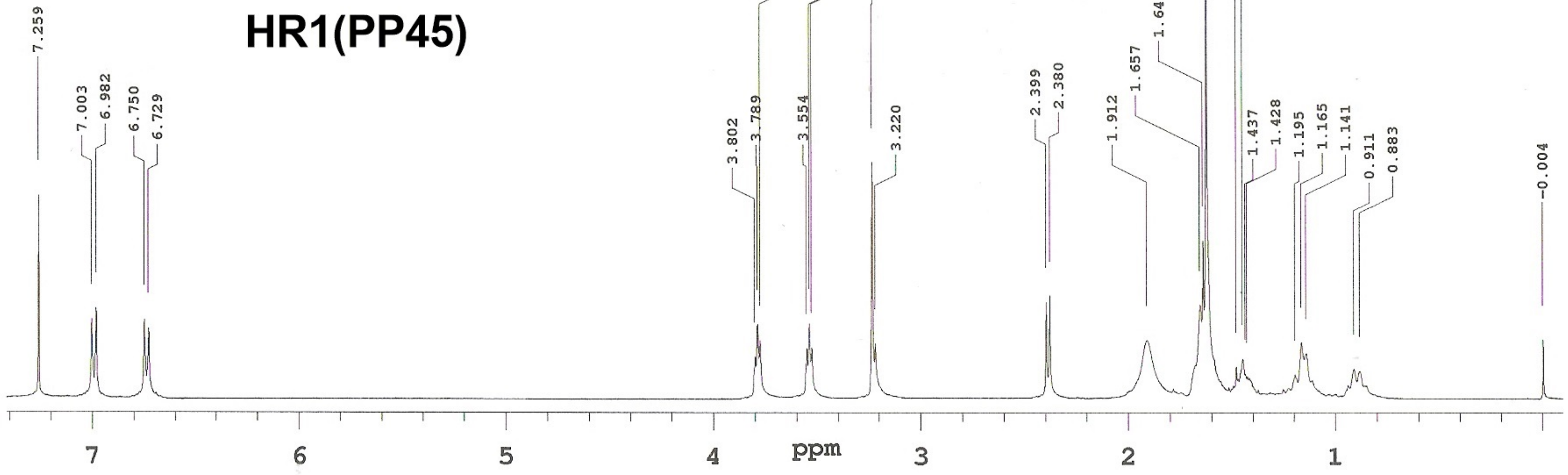

^{1}H-NMR ($CDCl_3$) Varian Mercury 400 Plus

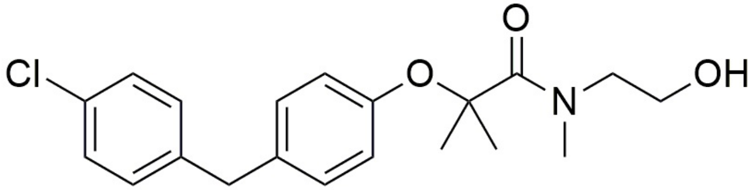

HR2(PP73)

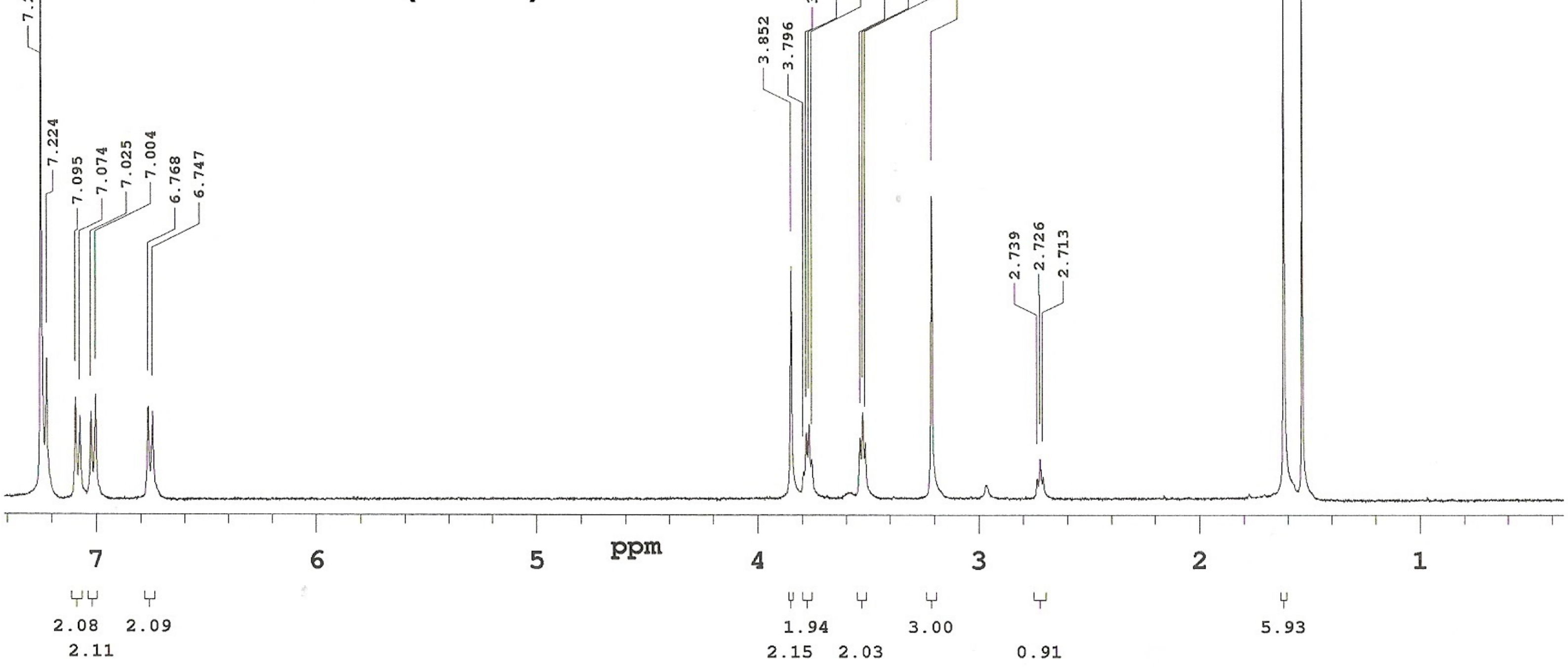

^{1}H-NMR ($CDCl_3$) Varian Mercury 400 Plus

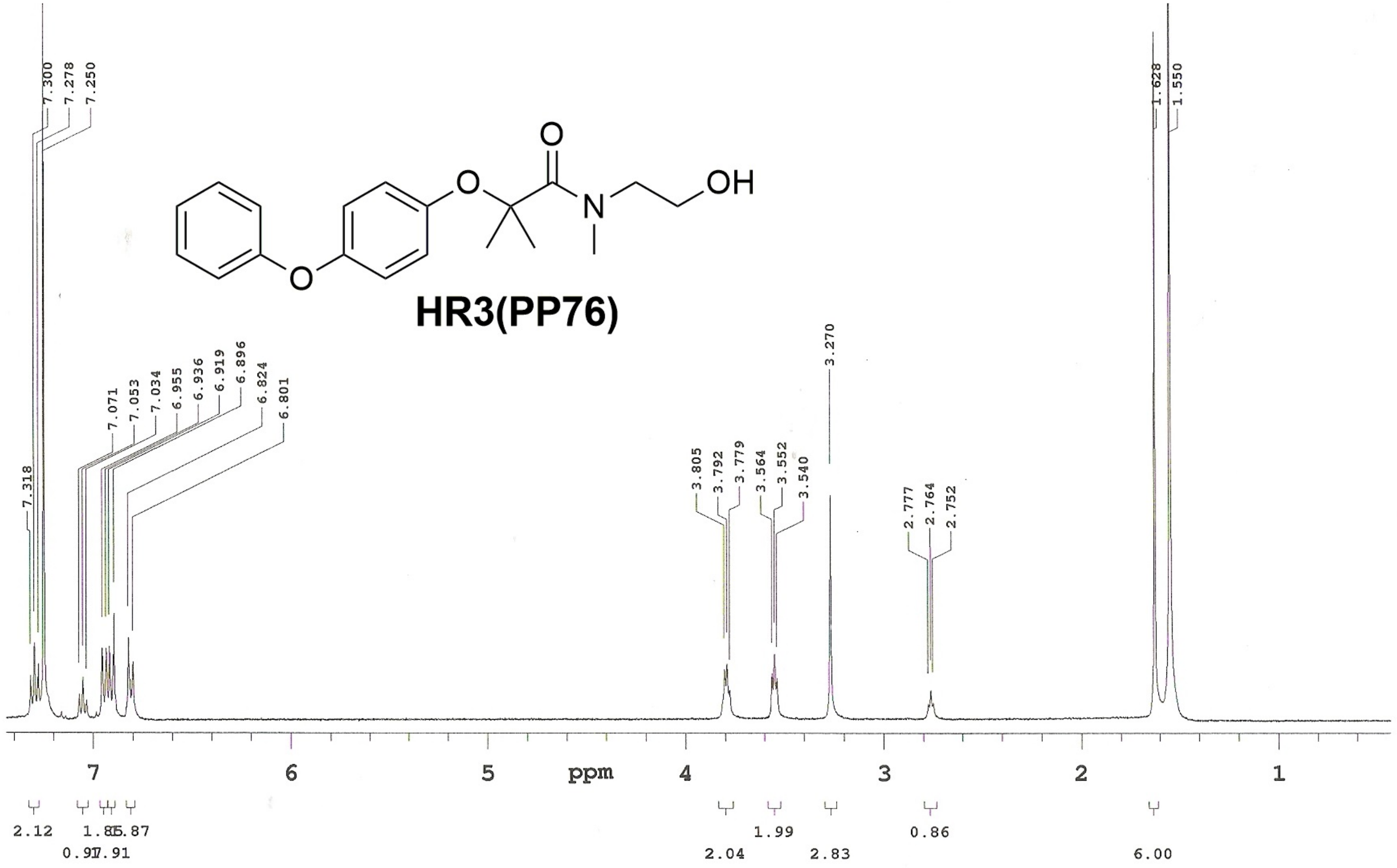

^{1}H-NMR ($CDCl_3$) Varian Mercury 300

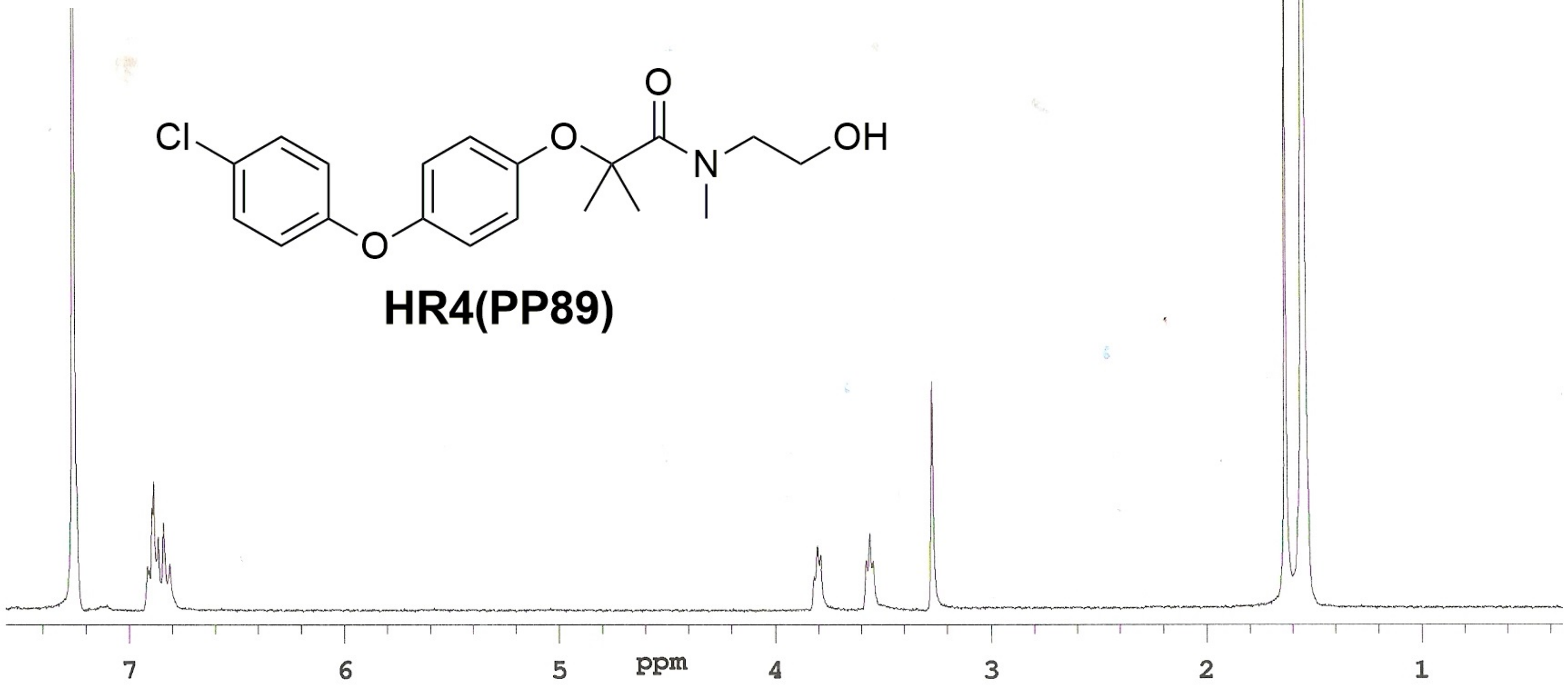

^{1}H-NMR ($CDCl_3$) Varian Mercury 300

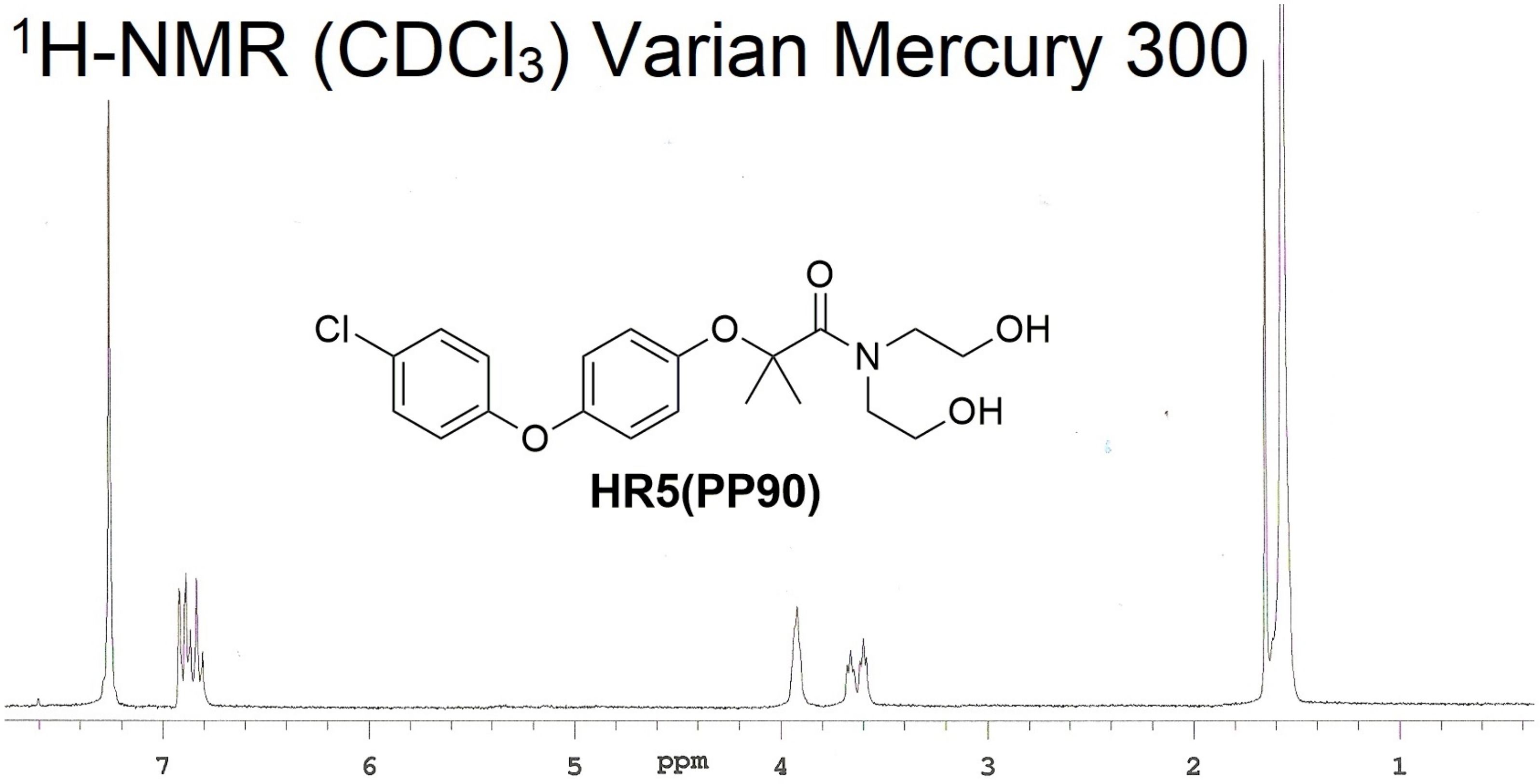

^{1}H-NMR ($CDCl_3$) Varian Mercury 300

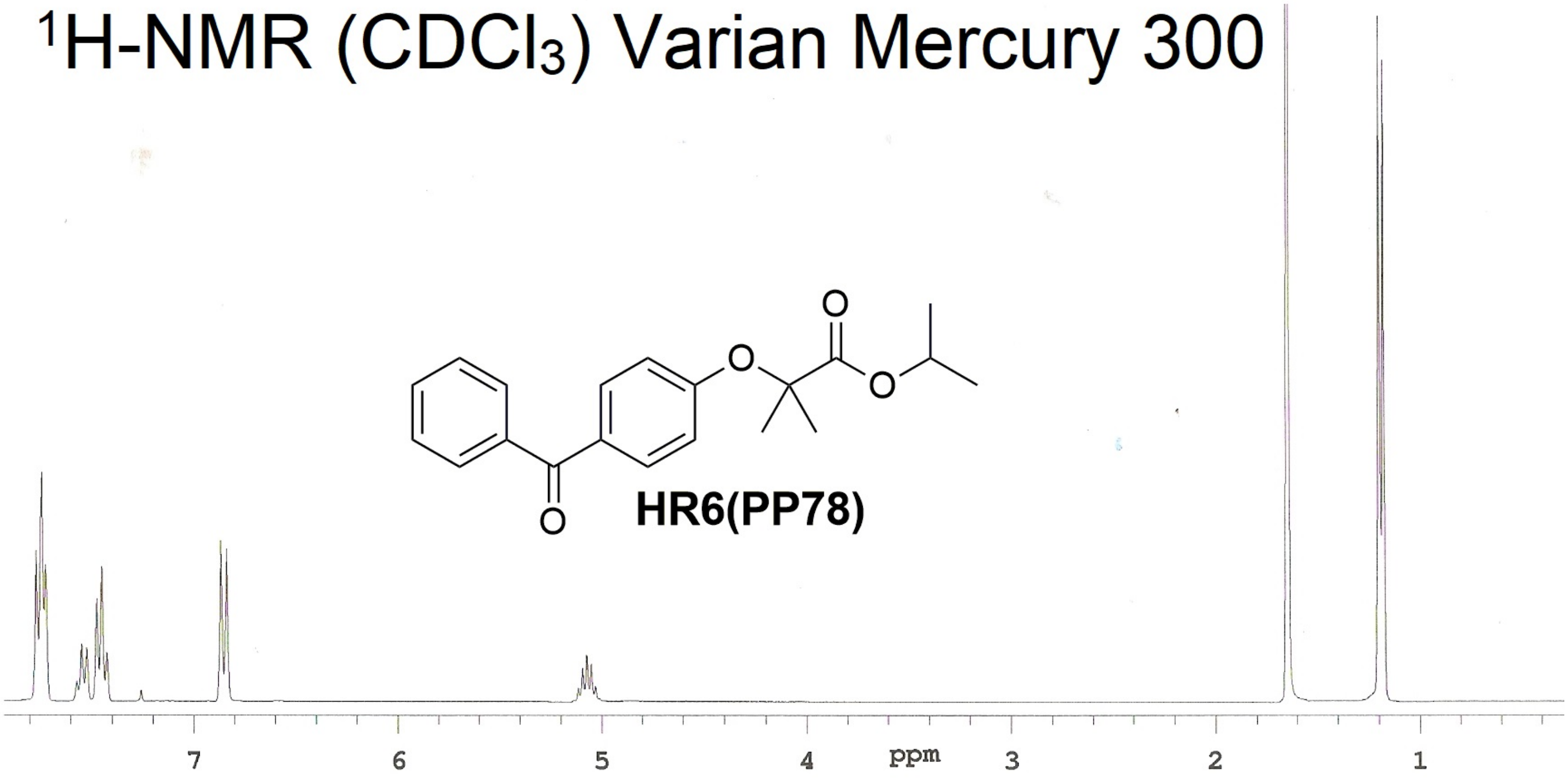

HR6(PP78)

^{13}C-NMR ($CDCl_3$) Varian Mercury 300

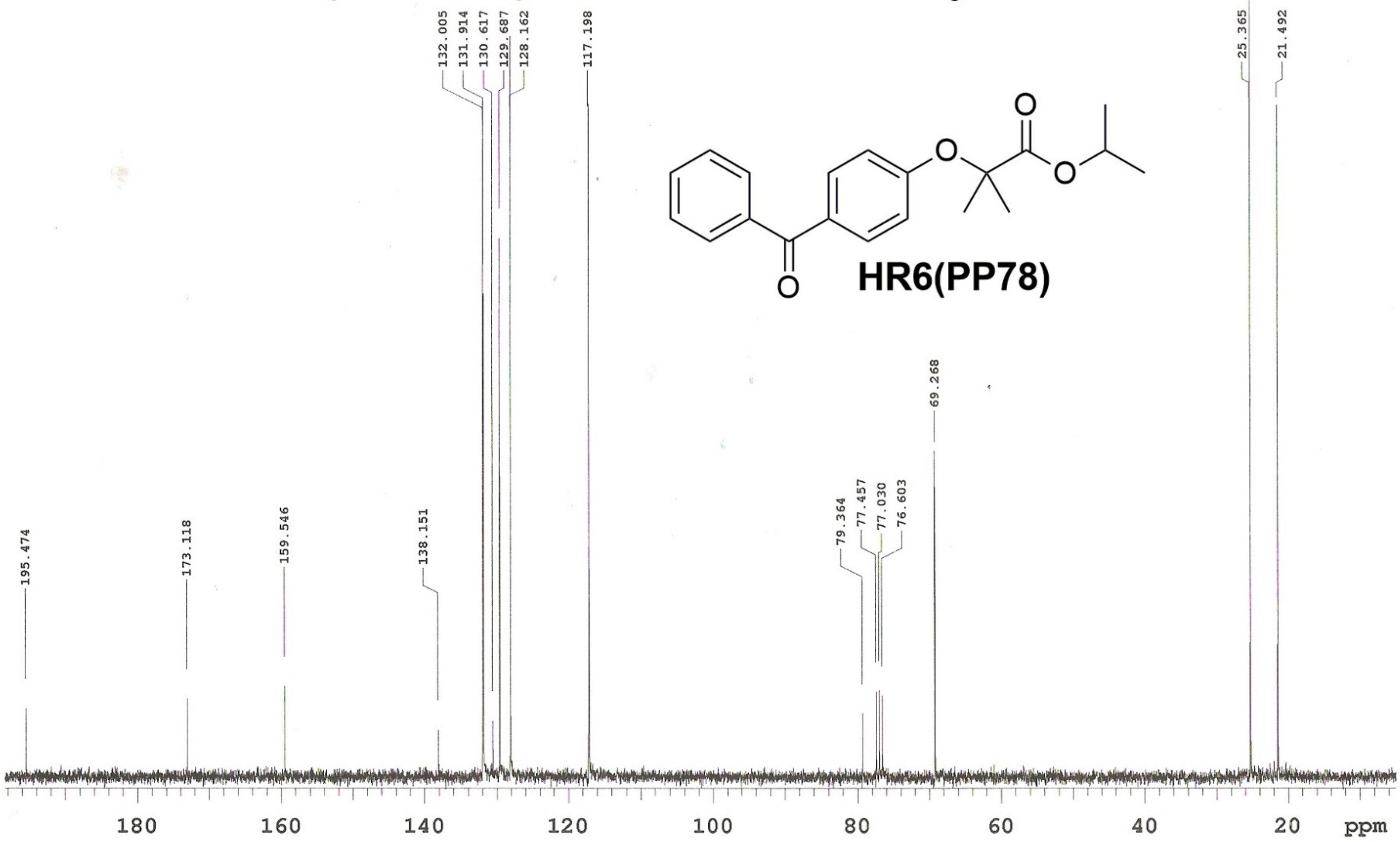

^{1}H-NMR ($CDCl_3$) Varian Mercury 300

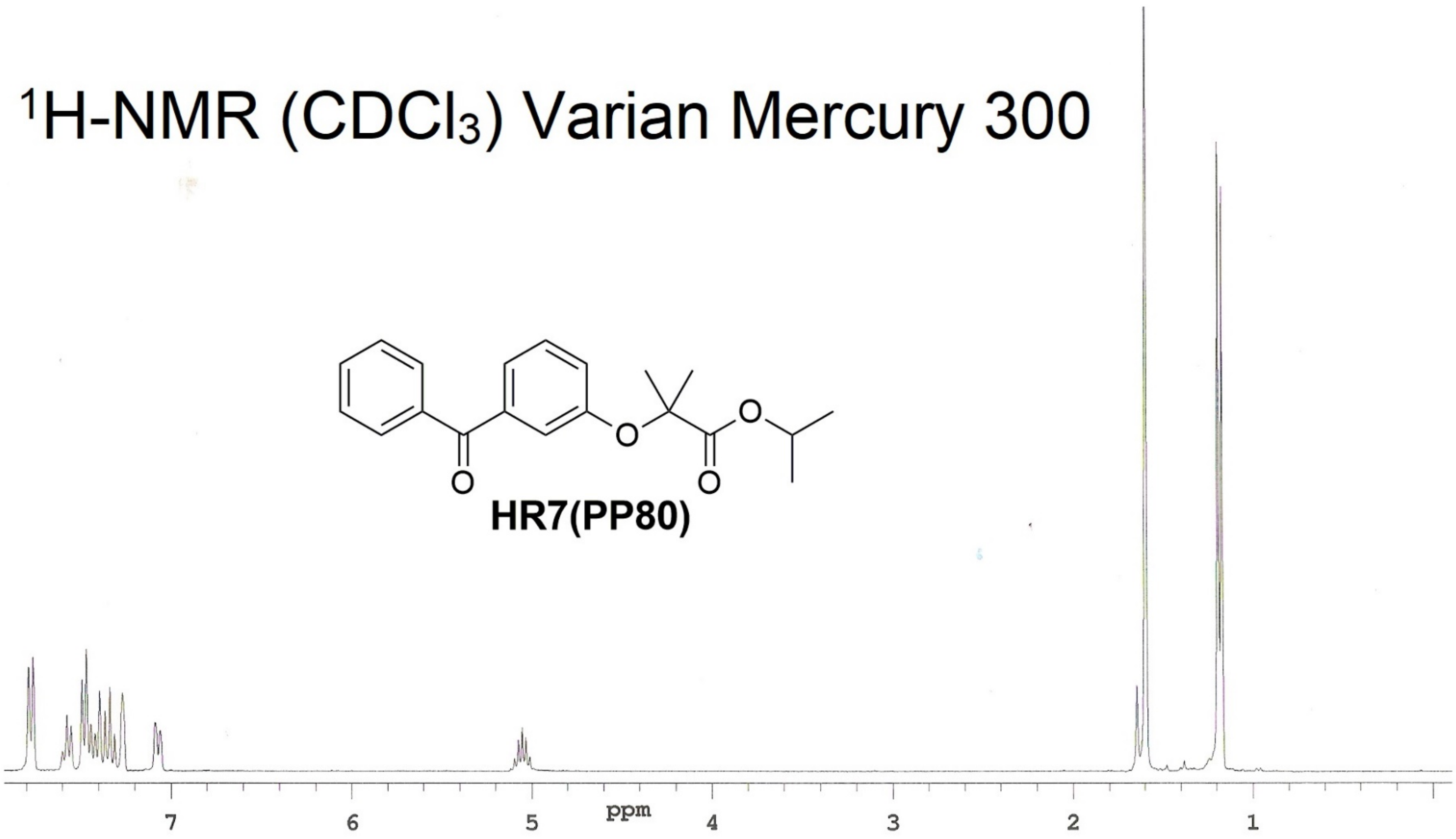

HR7(PP80)

^{13}C-NMR ($CDCl_3$) Varian Mercury 300

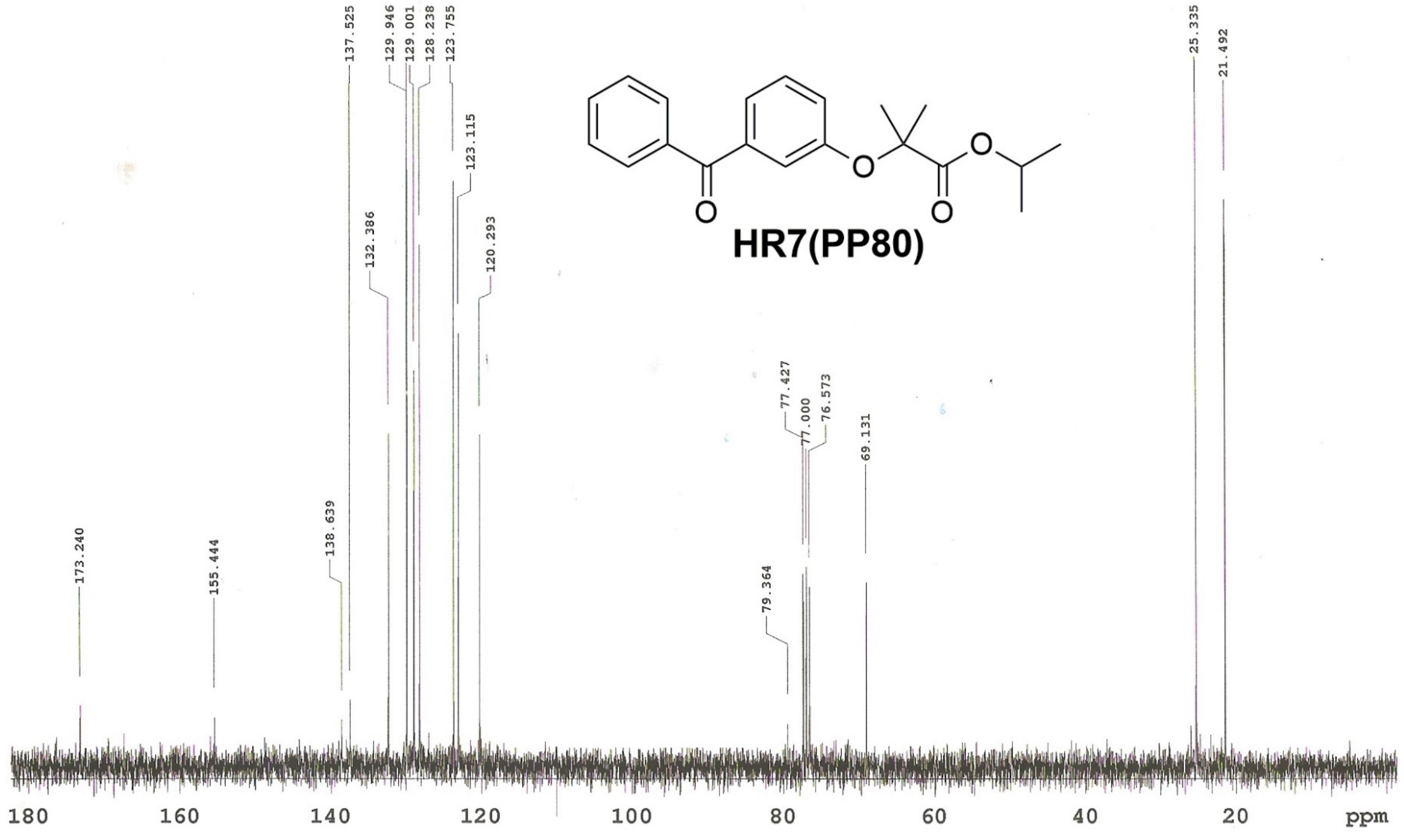

^{1}H-NMR ($CDCl_3$) Varian Mercury 300

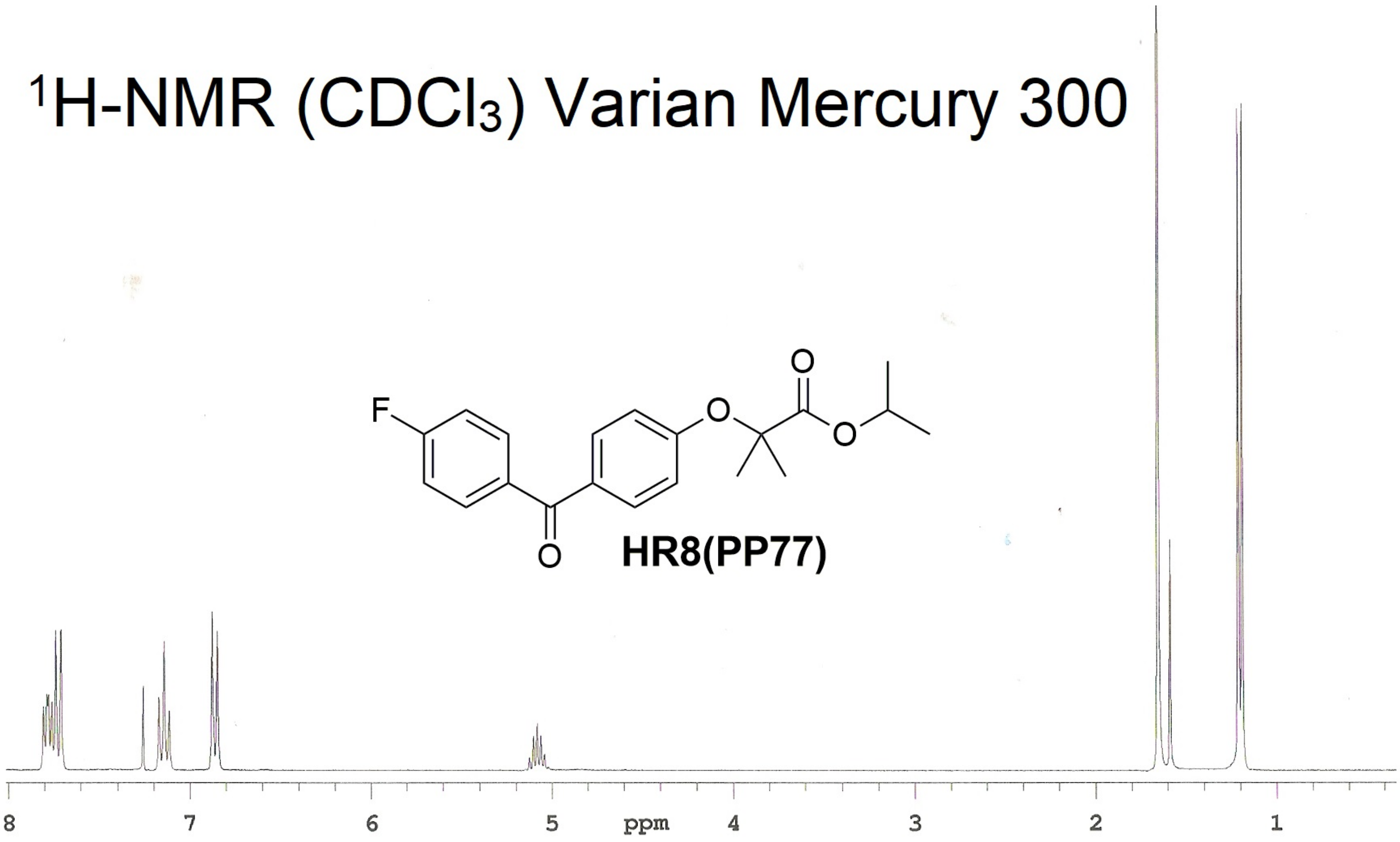

HR8(PP77)

^{1}H-NMR ($CDCl_3$) Varian Mercury 300

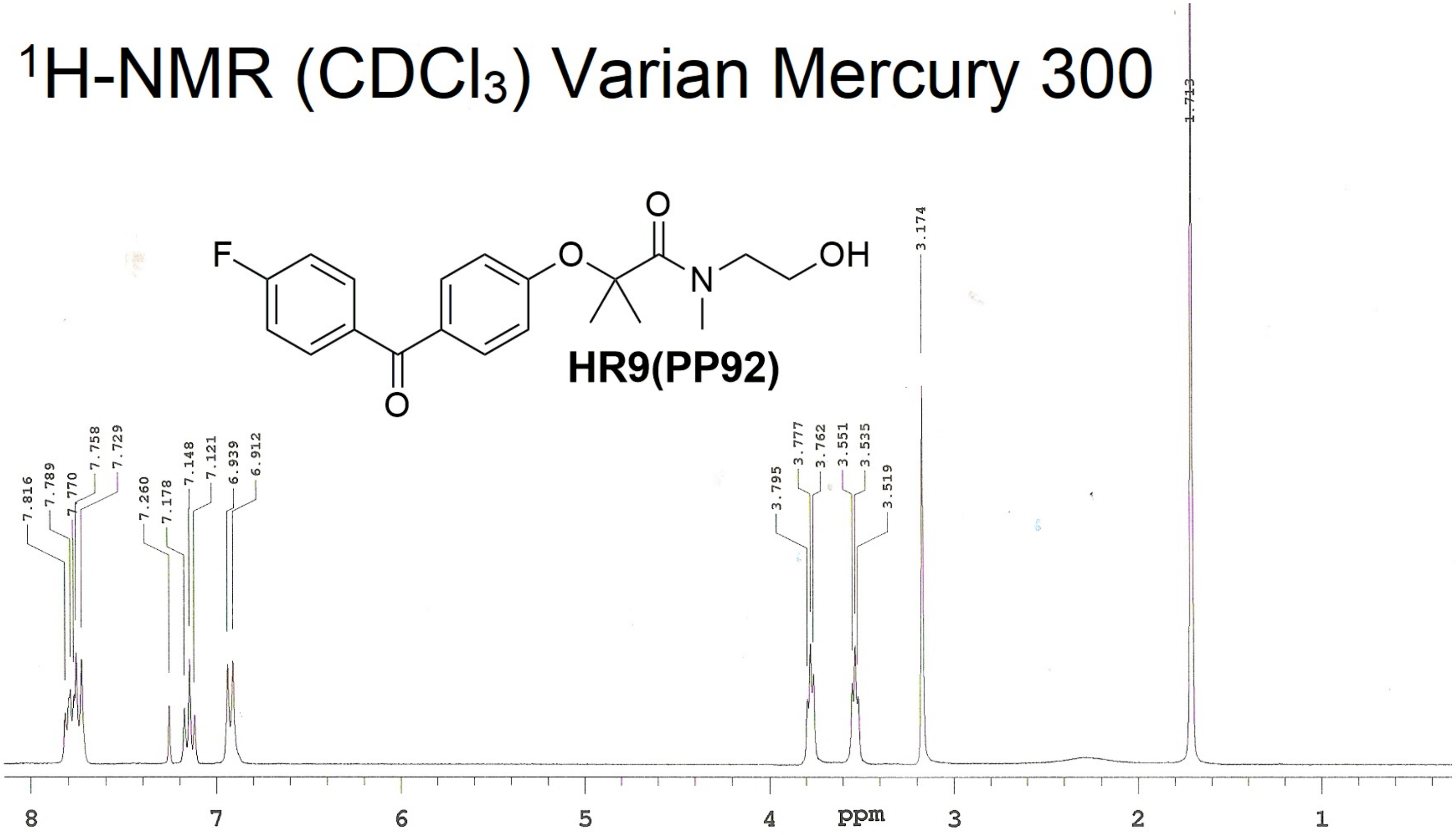

HR9(PP92)

^{13}C-NMR ($CDCl_3$) Varian Mercury 300

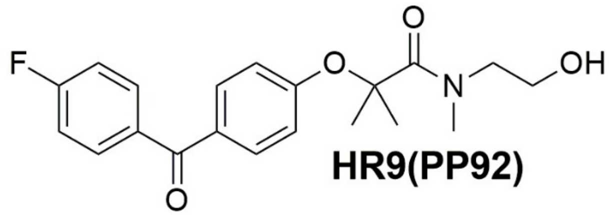

HR9(PP92)

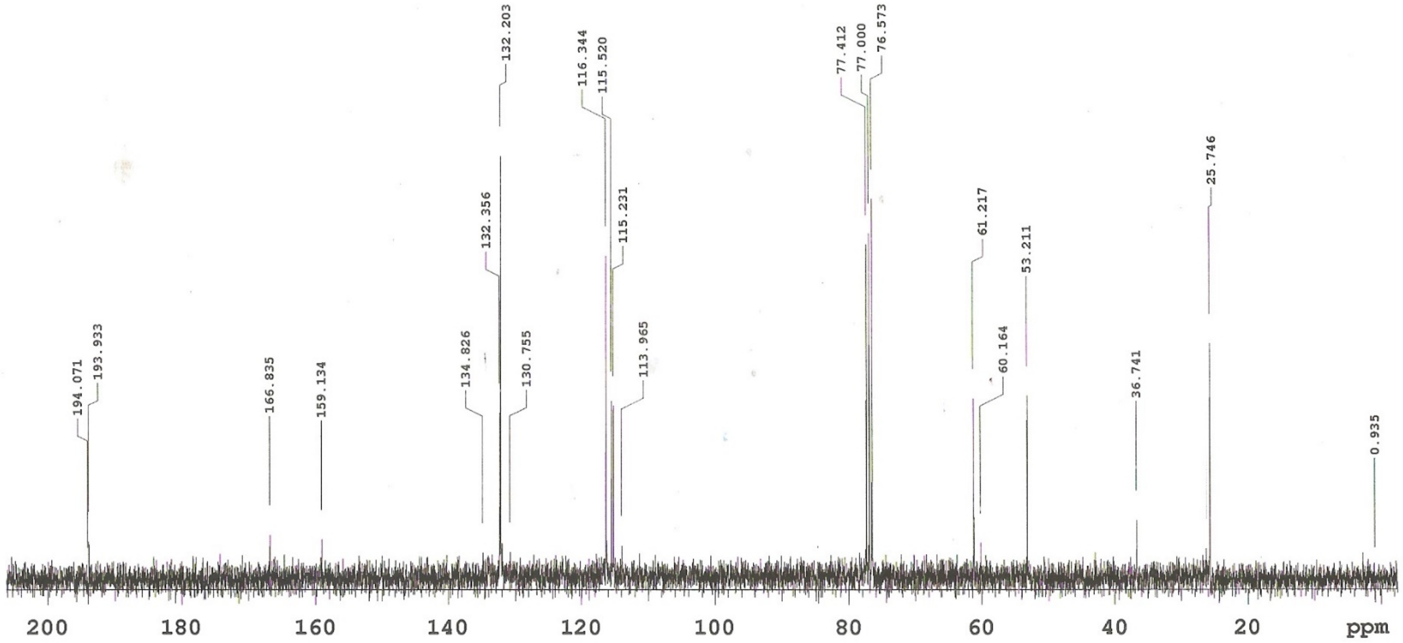

^{1}H-NMR ($CDCl_3$) Varian Mercury 300

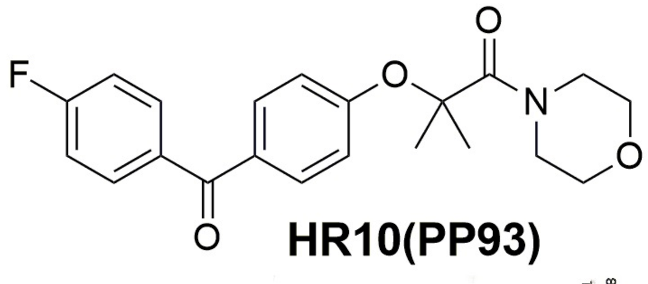

HR10(PP93)

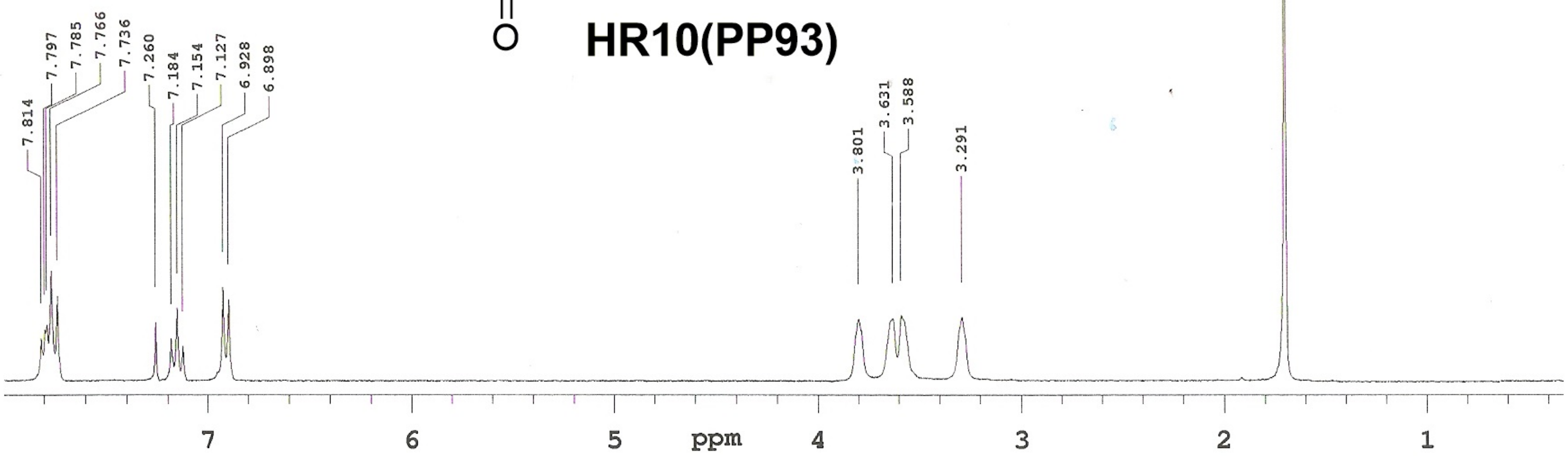

^{13}C-NMR ($CDCl_3$) Varian Mercury 300

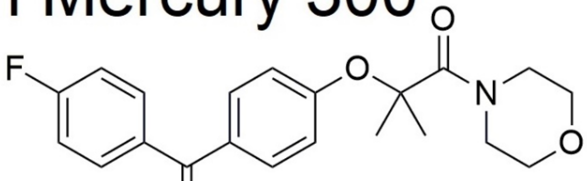

HR10(PP93)

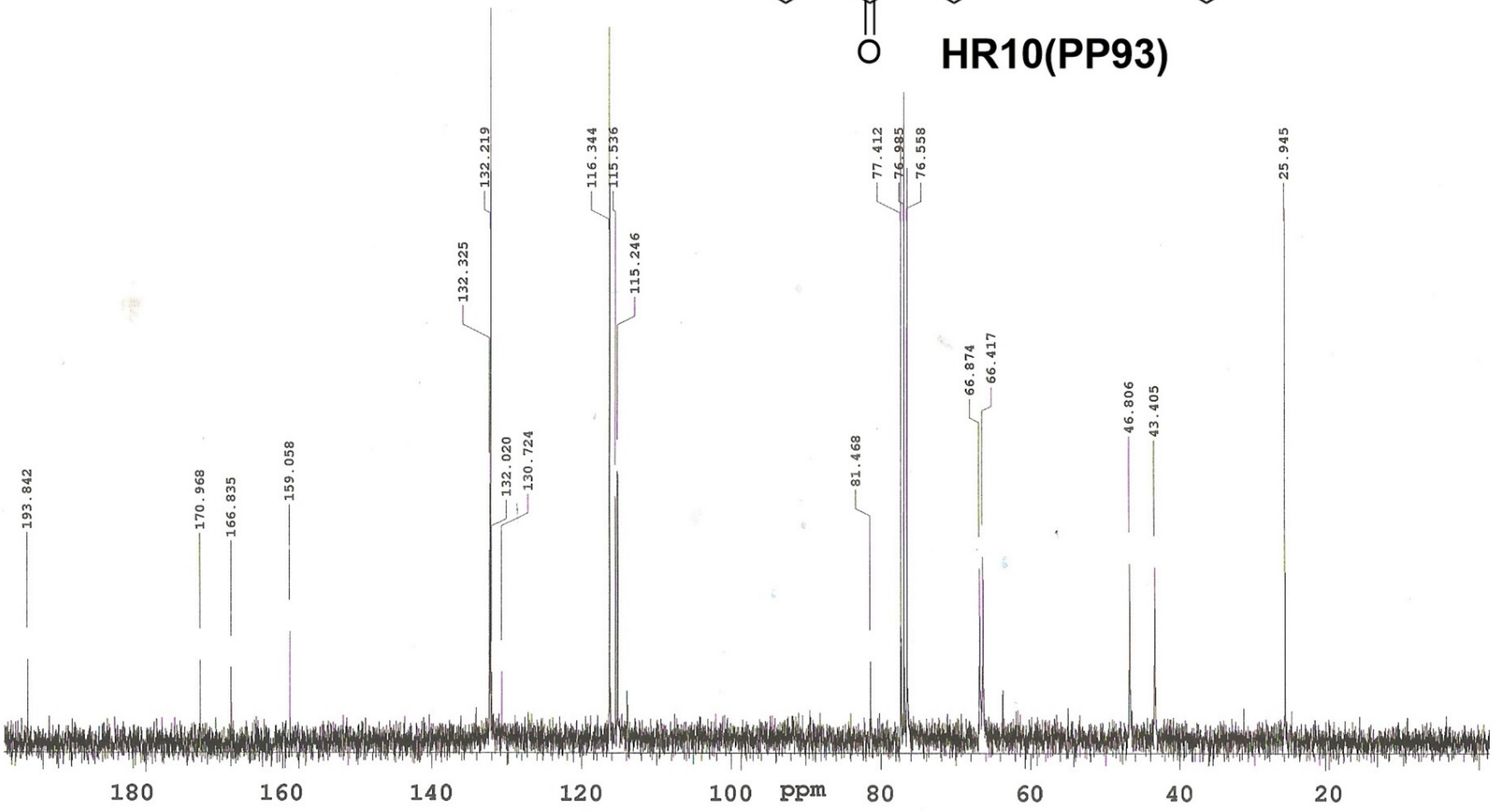

^{1}H-NMR ($CDCl_3$) Varian Mercury 300

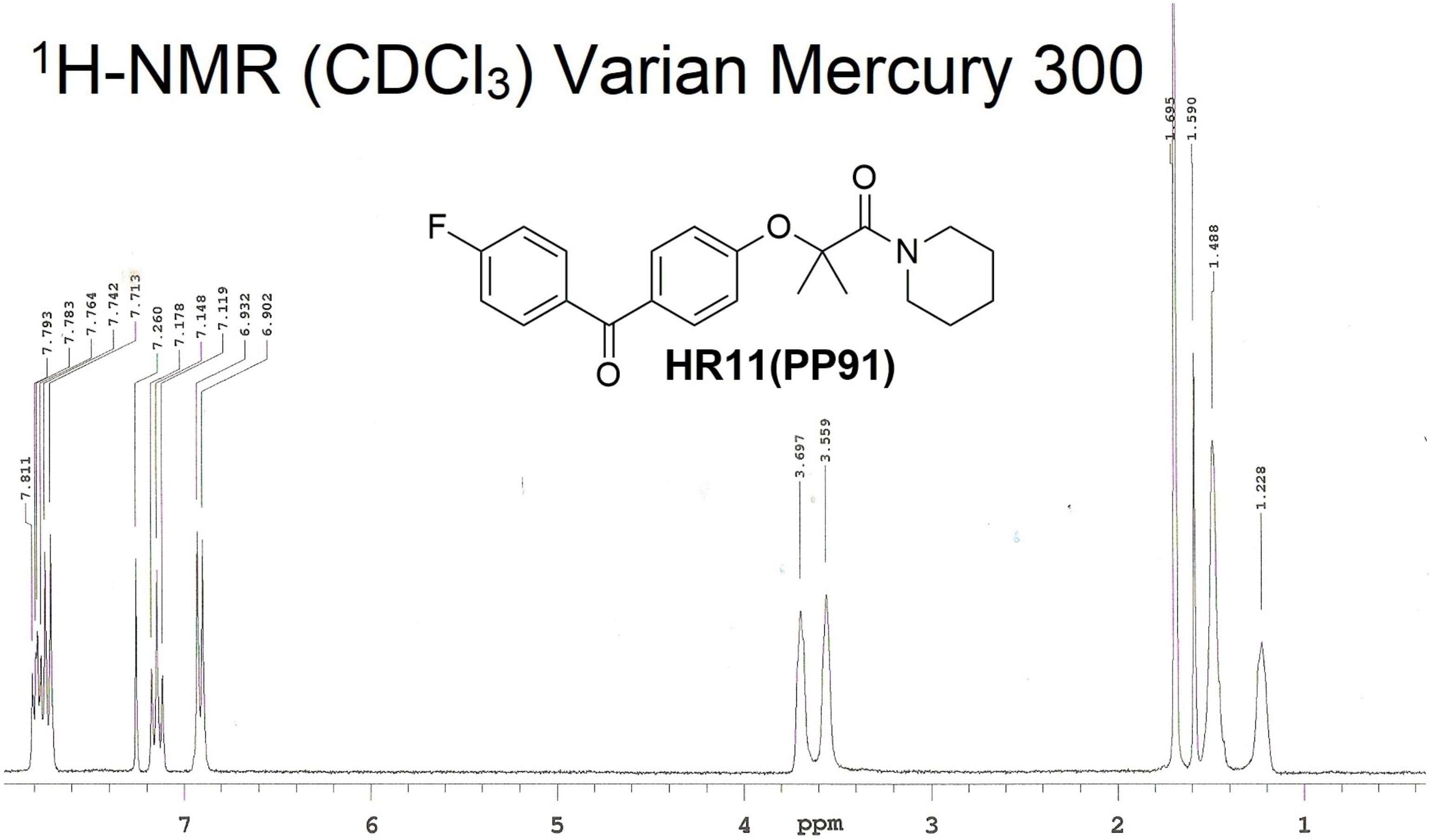

^{13}C-NMR ($CDCl_3$) Varian Mercury 300

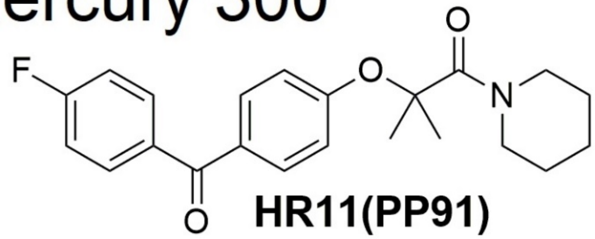

HR11(PP91)

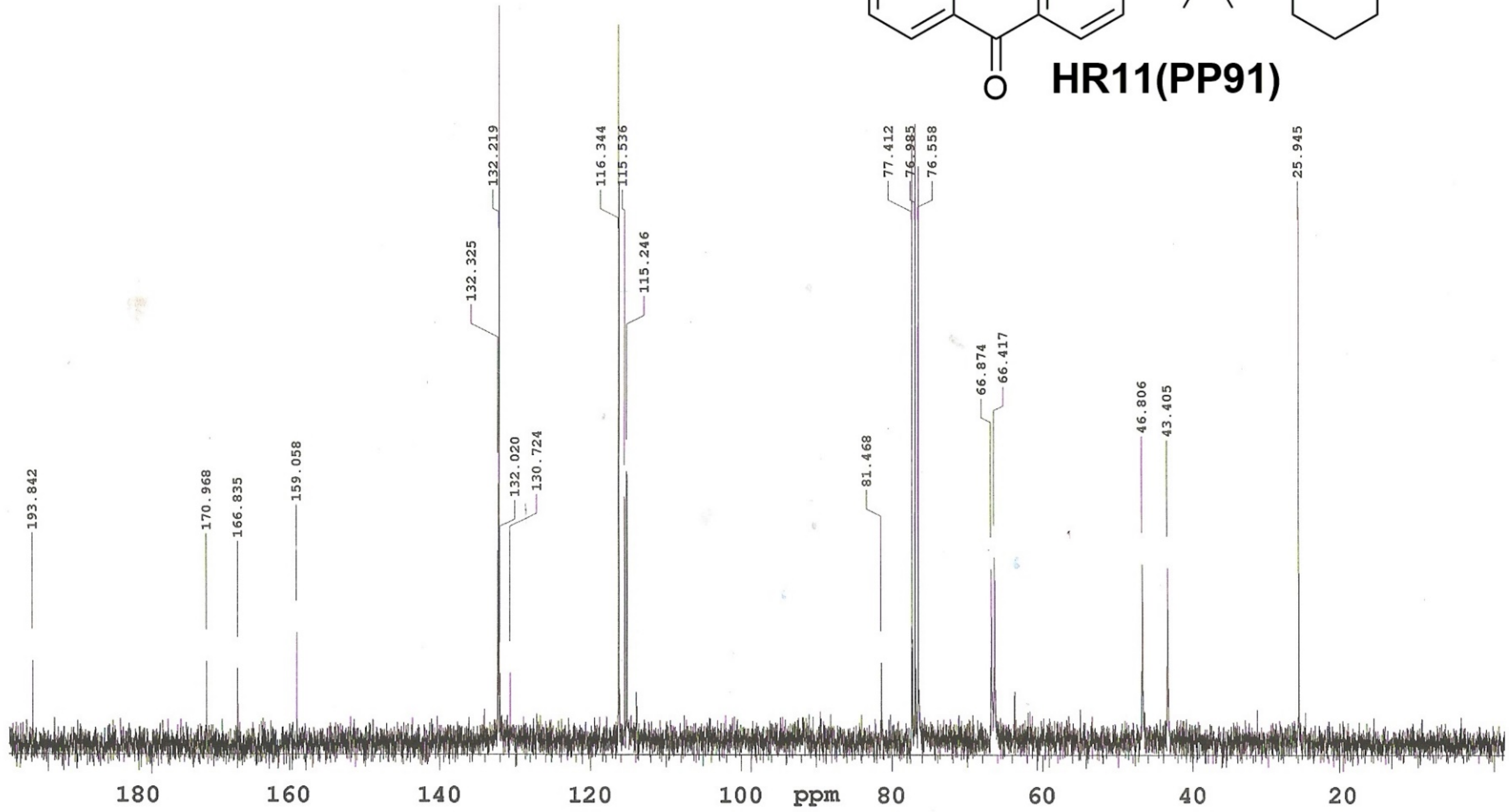

^{1}H-NMR ($CDCl_3$) Varian Mercury 400 Plus

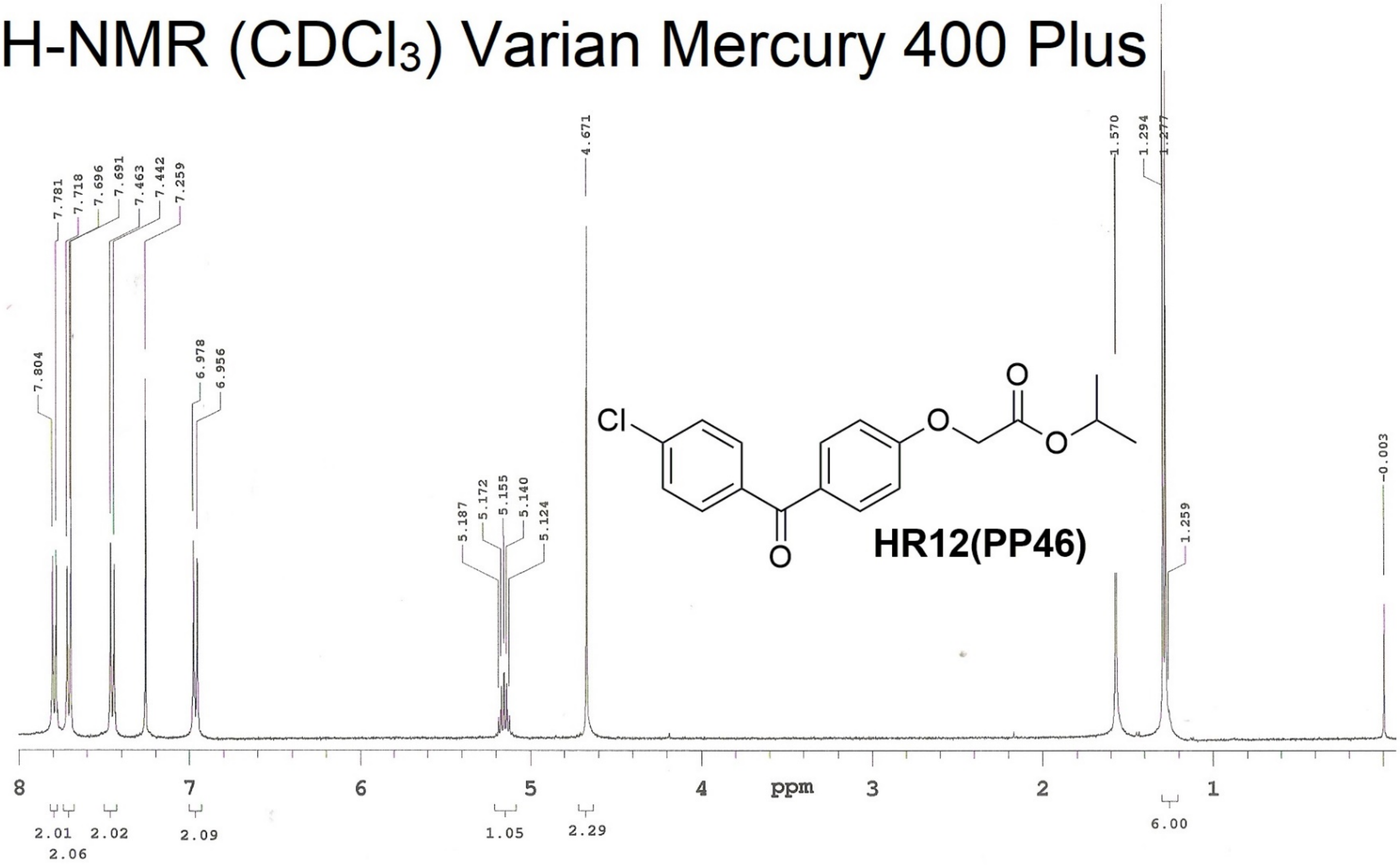

^{1}H-NMR ($CDCl_3$) Varian Mercury 400 Plus

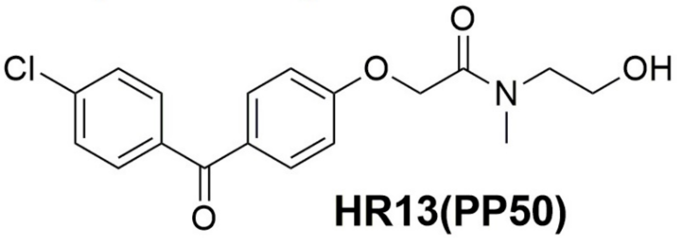

HR13(PP50)

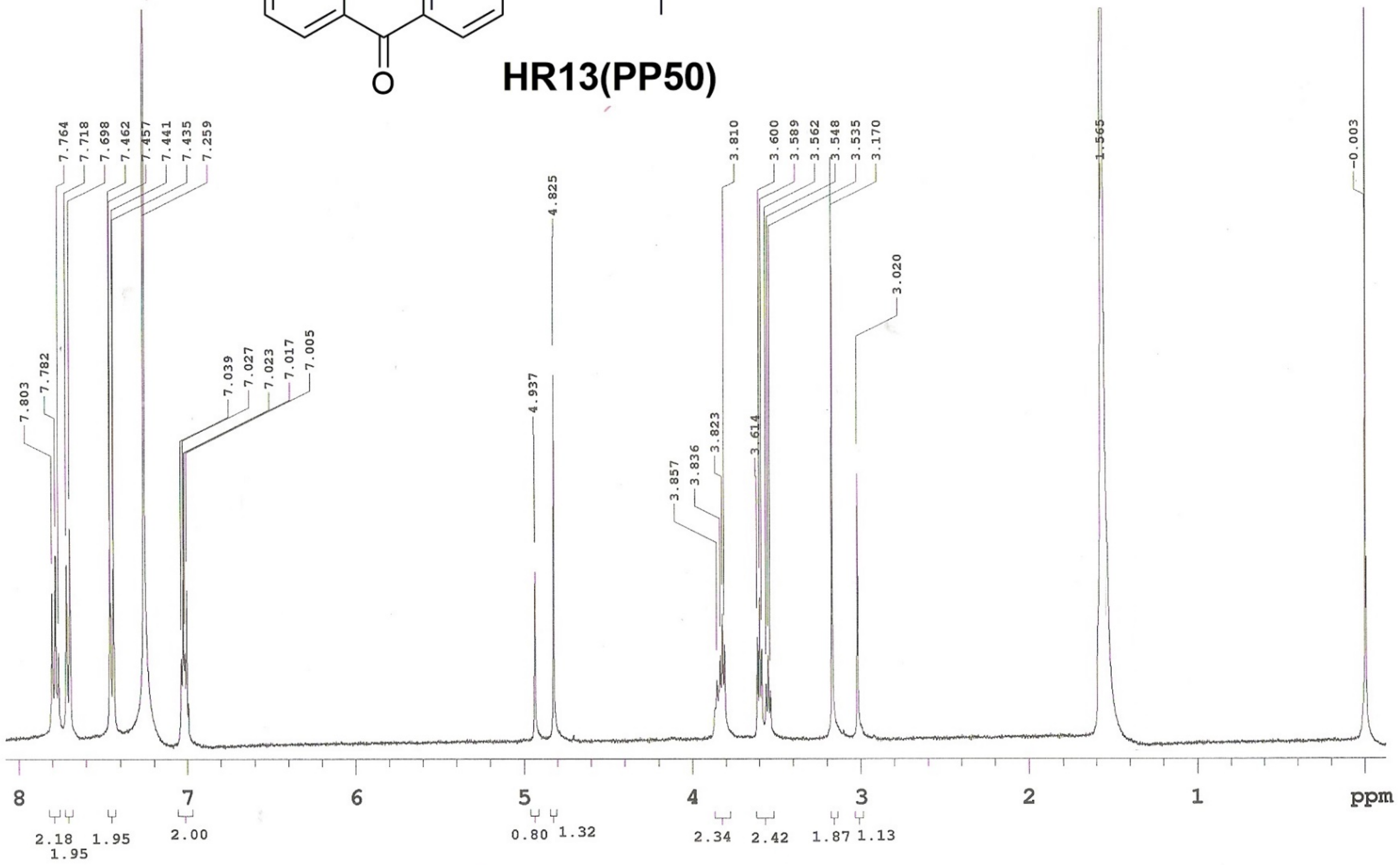

^{1}H-NMR ($CDCl_3$) Varian Mercury 400 Plus

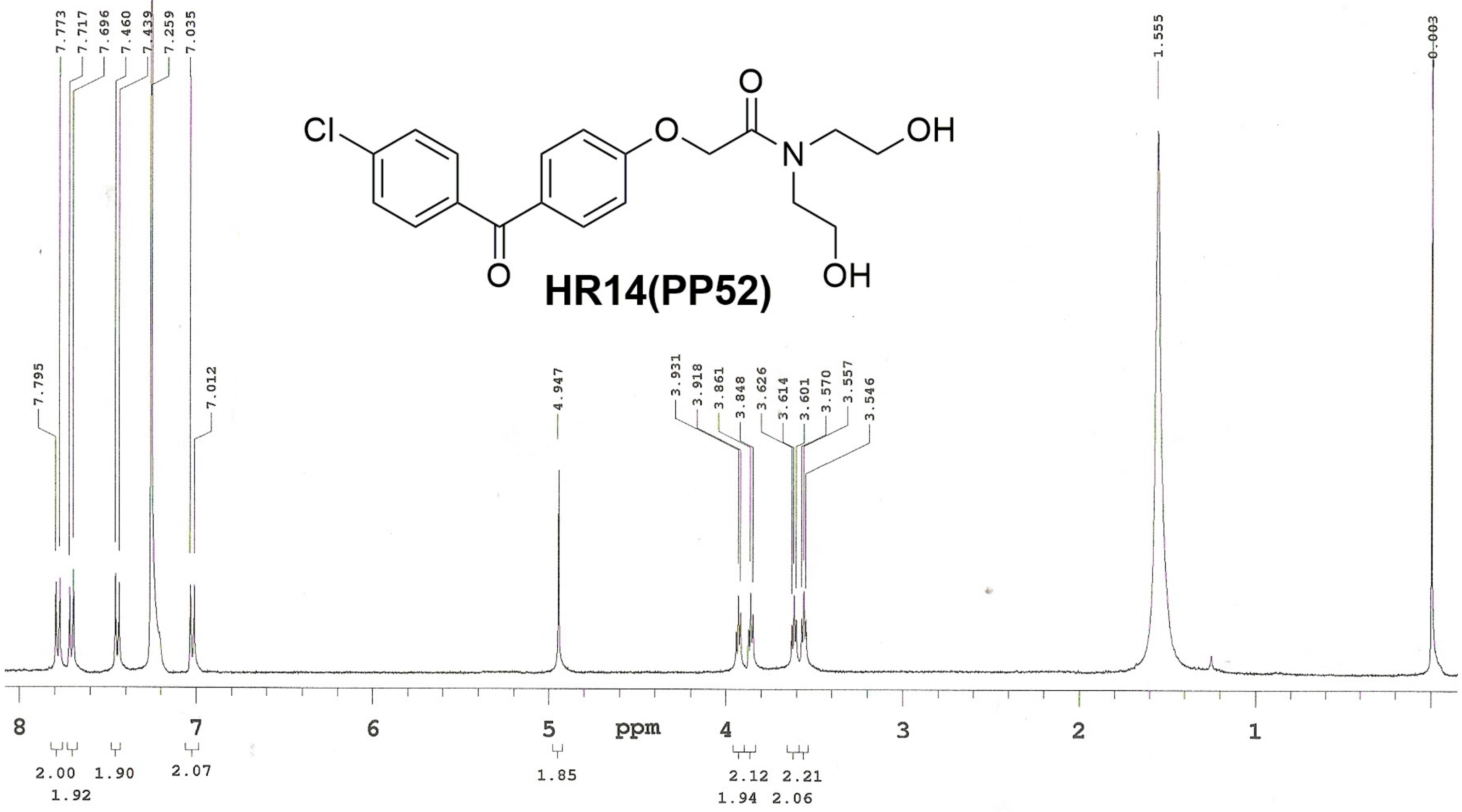

^{1}H-NMR ($CDCl_3$) Varian Mercury 400 Plus

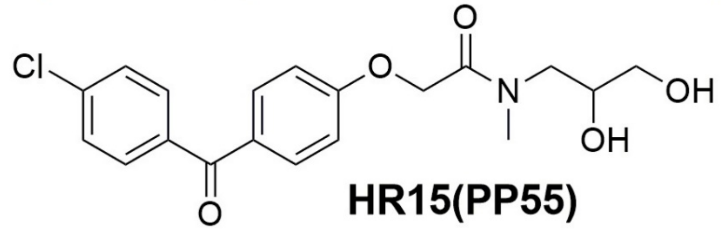

HR15(PP55)

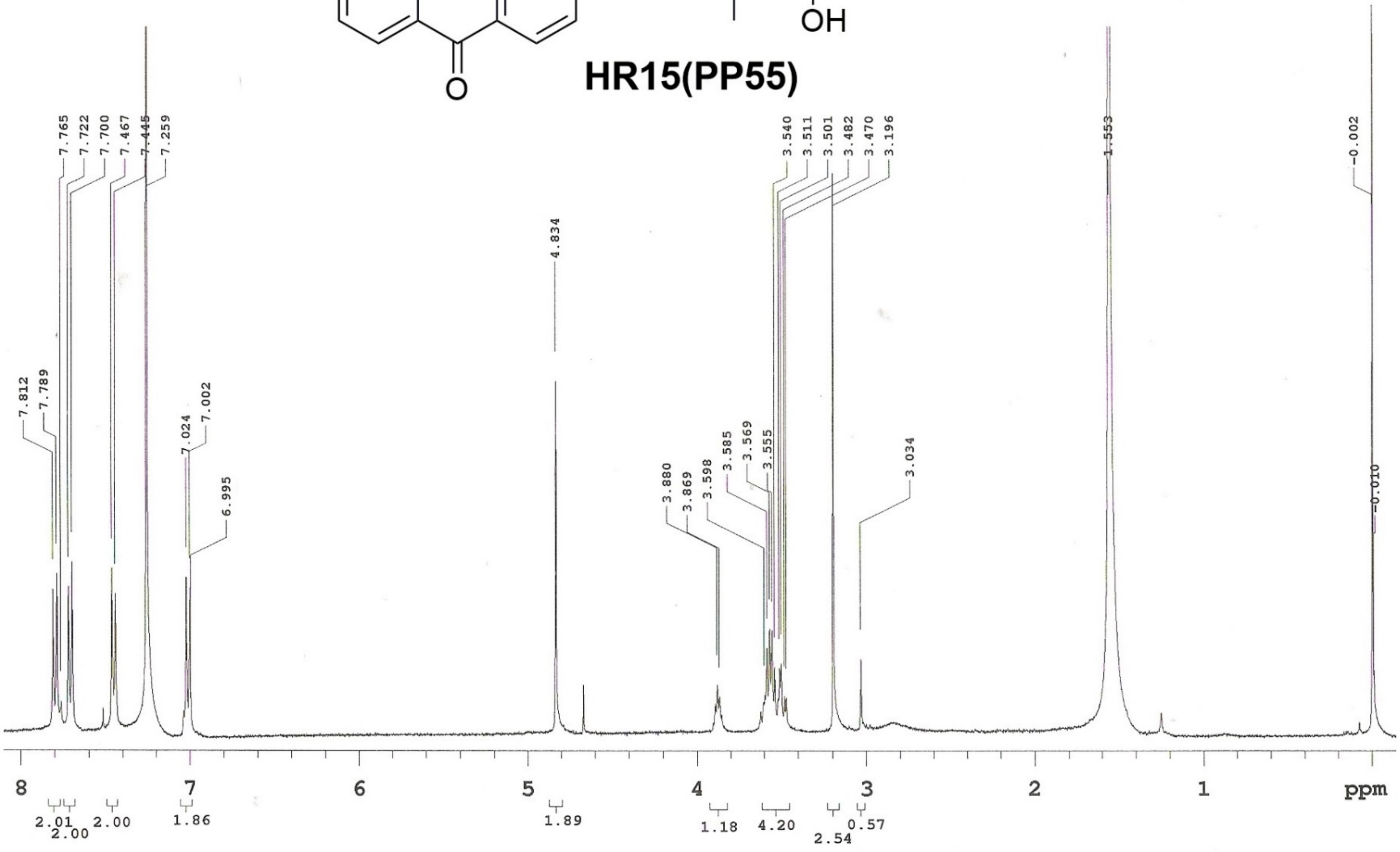

^{1}H-NMR ($CDCl_3$) Varian Mercury 400 Plus

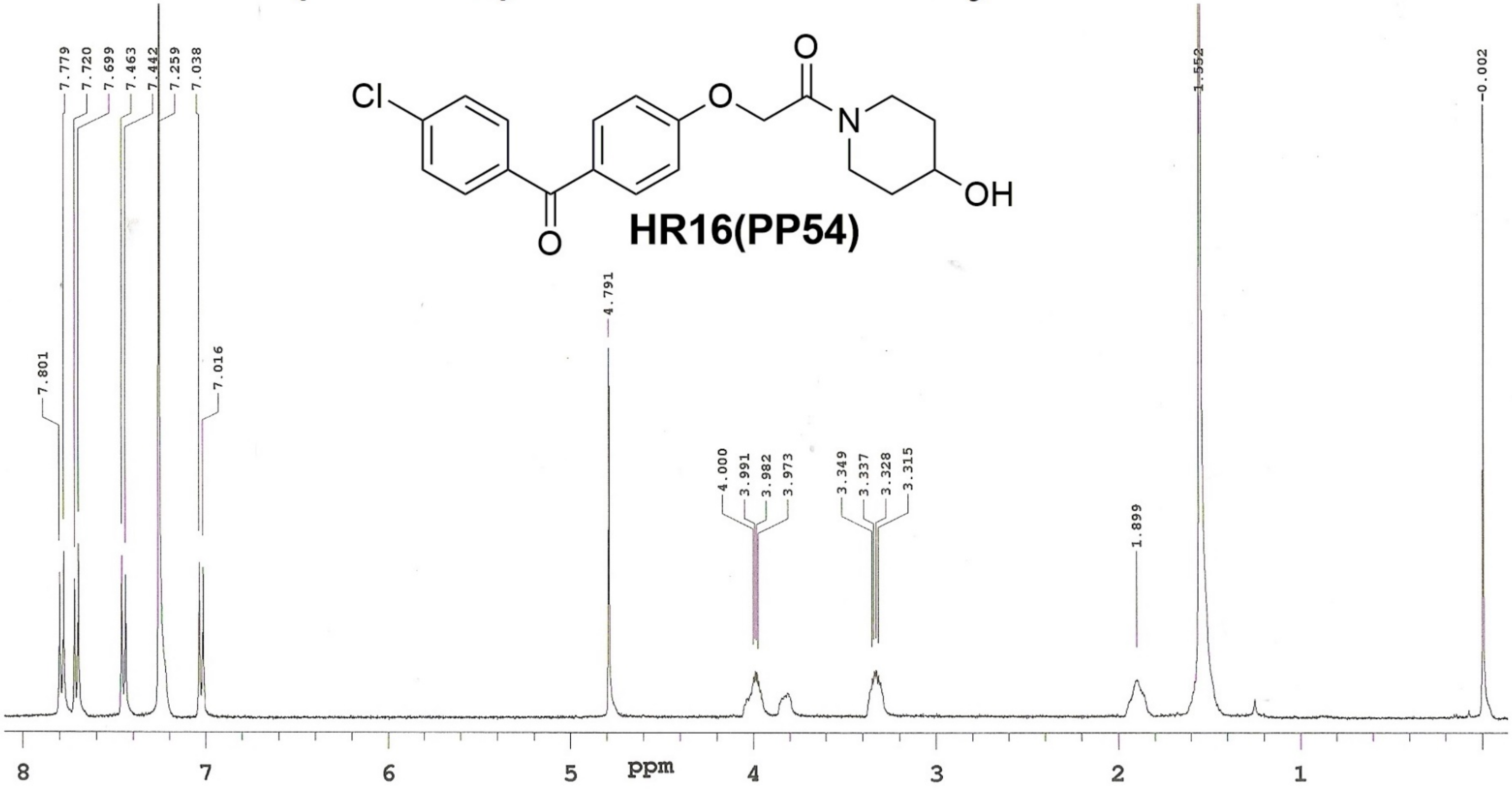

^{1}H-NMR ($CDCl_3$) Varian Mercury 400 Plus

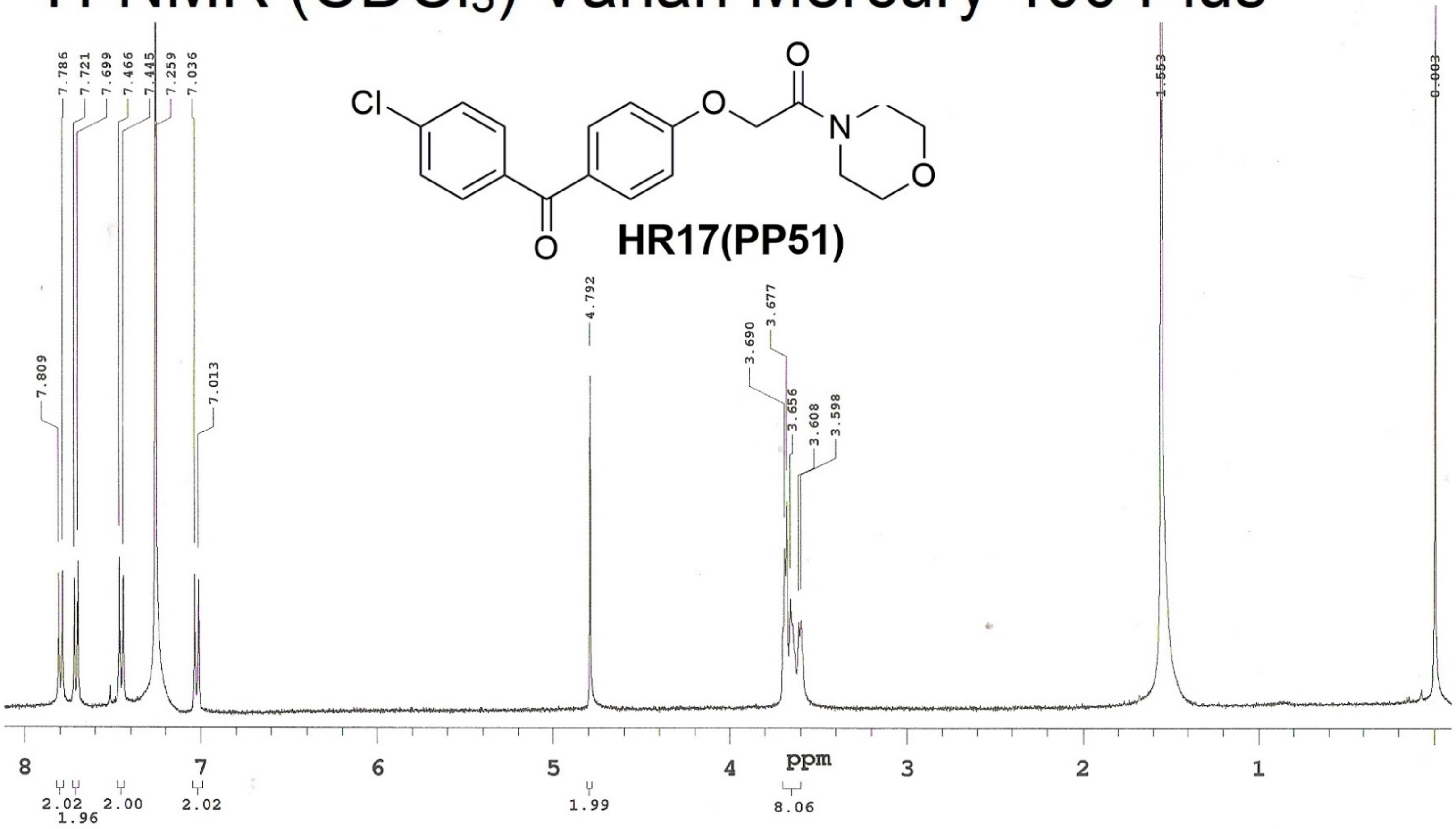

^{1}H-NMR ($CDCl_3$) Varian Mercury 400 Plus

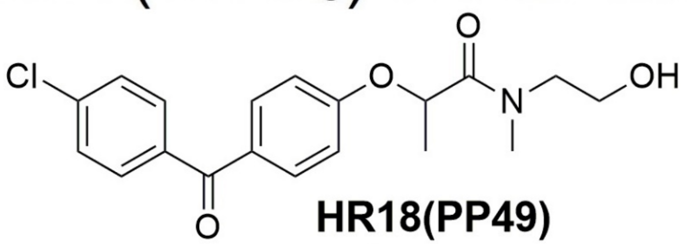

HR18(PP49)

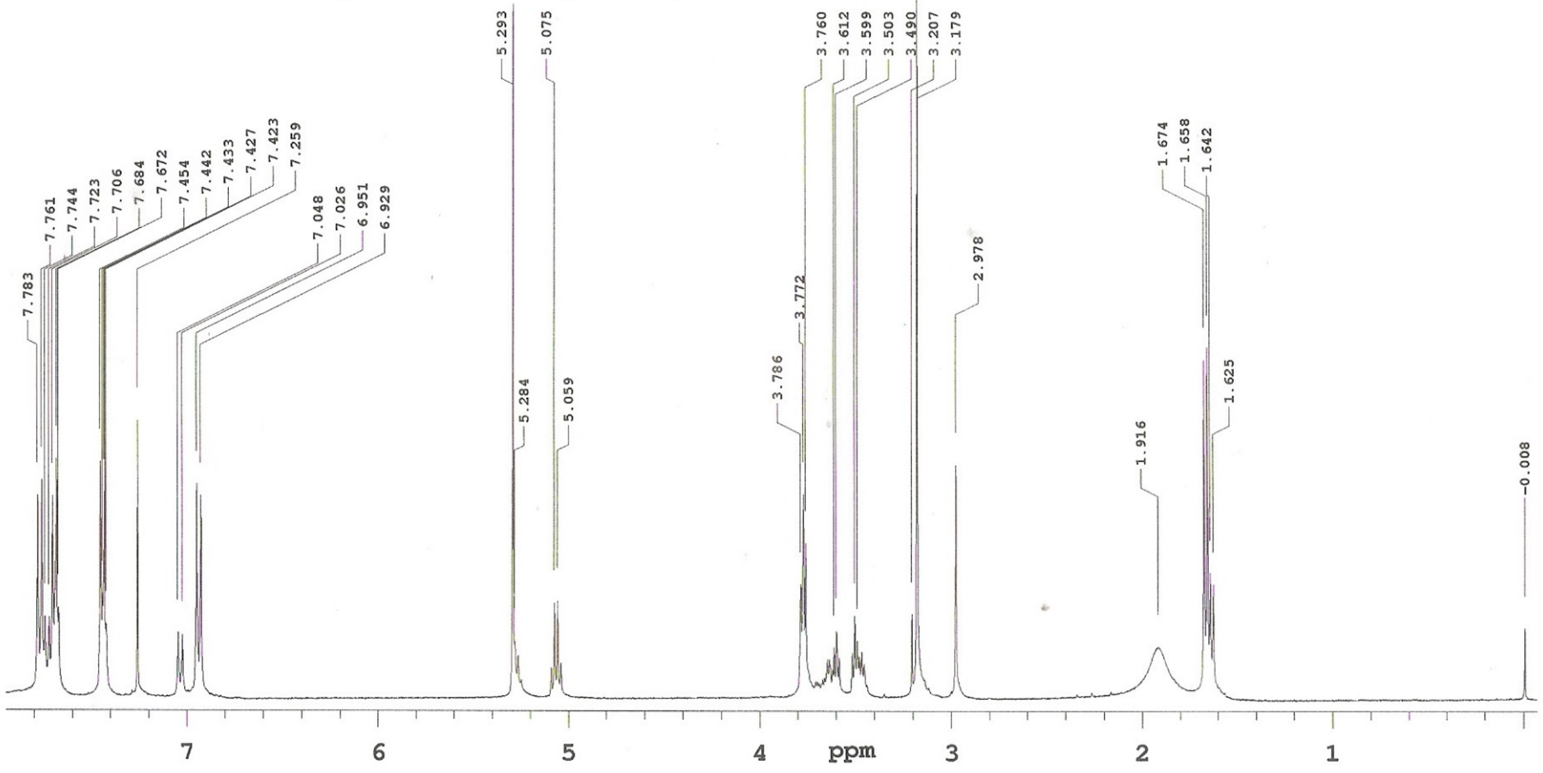

^{1}H-NMR ($CDCl_3$) Varian Mercury 400 Plus

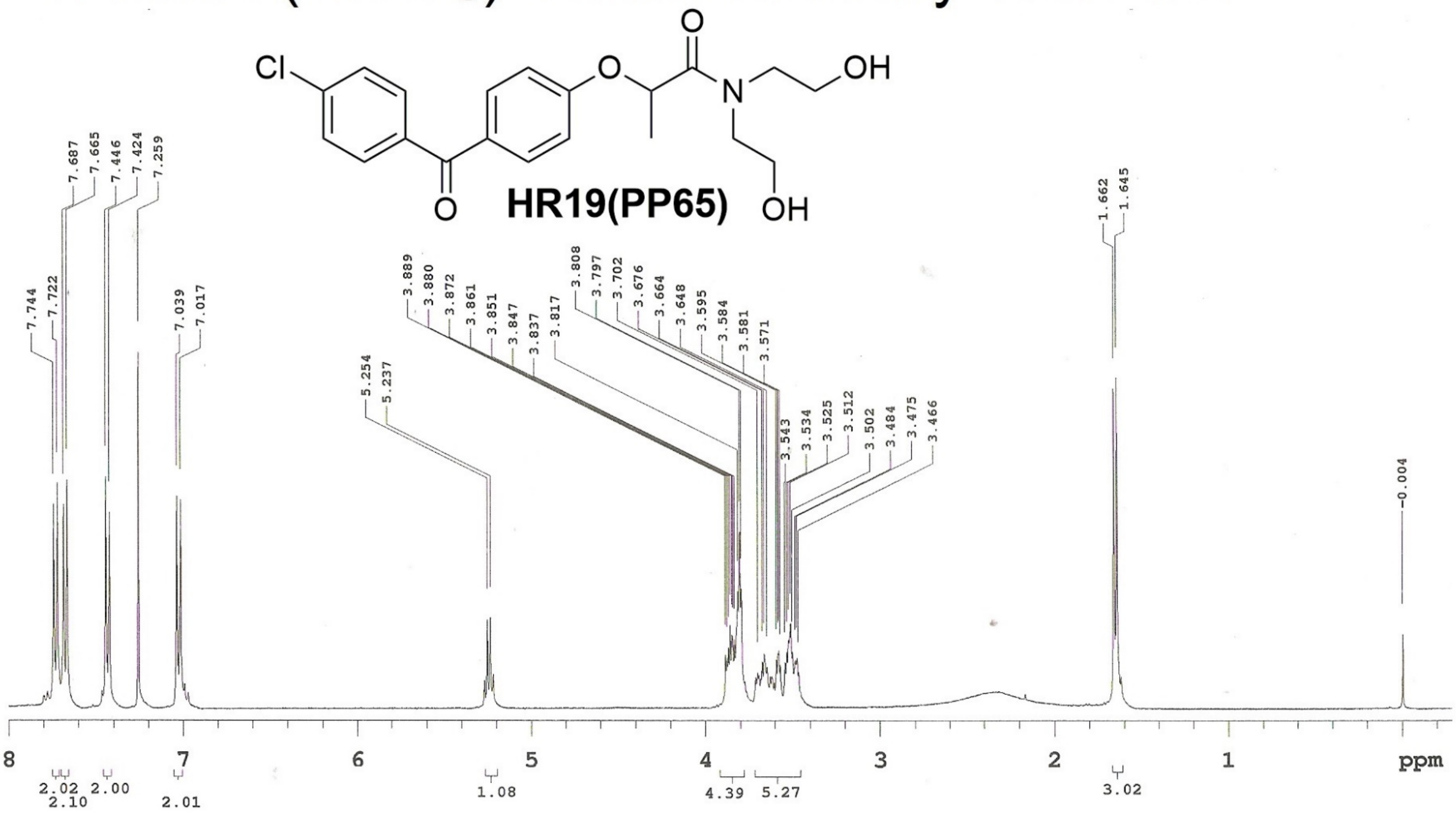

HR19(PP65)

^{1}H-NMR ($CDCl_3$) Varian Mercury 400 Plus

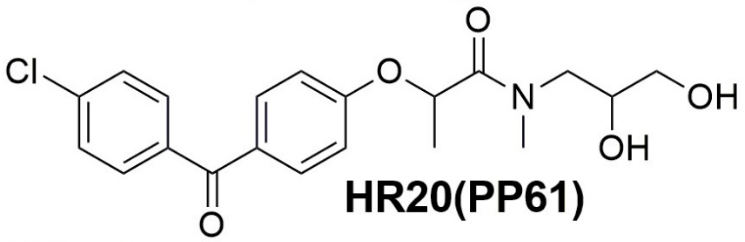

HR20(PP61)

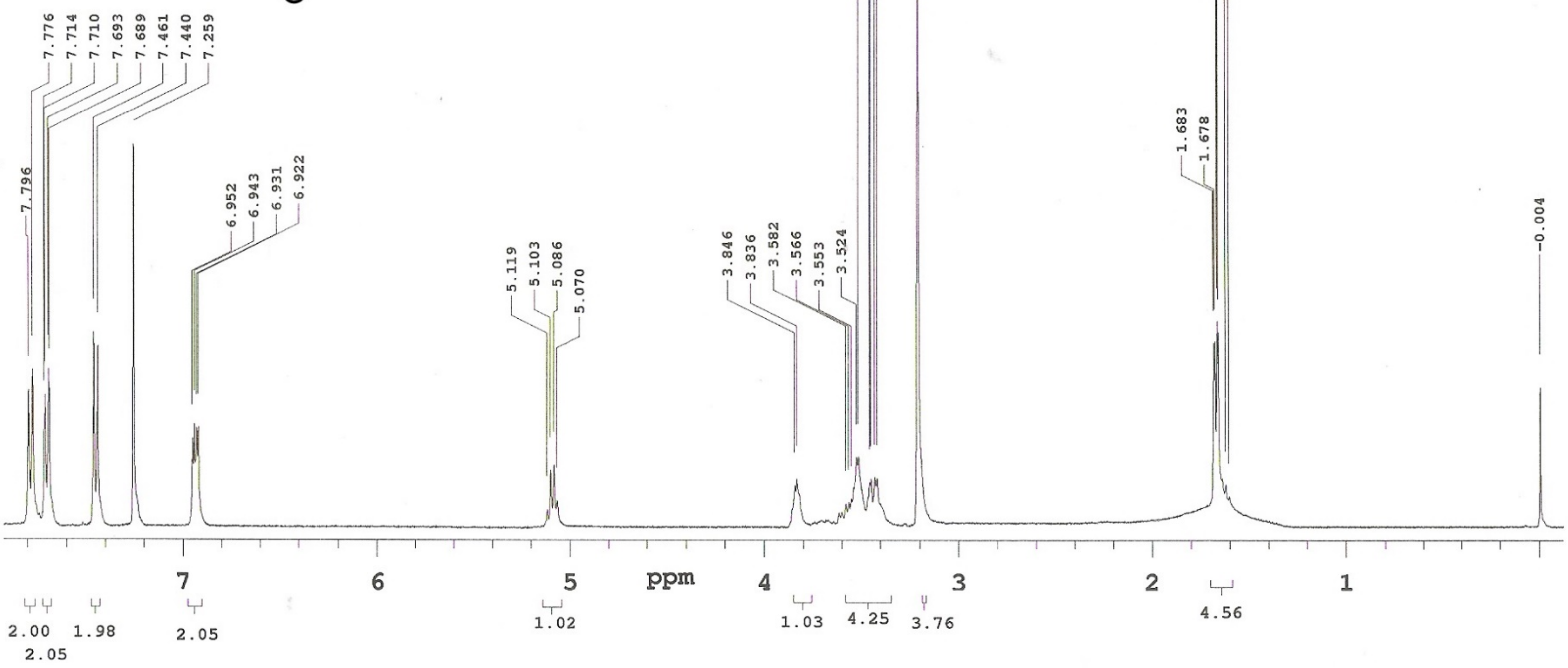

^{1}H-NMR (DMSO-d_6) Varian Mercury 400 Plus

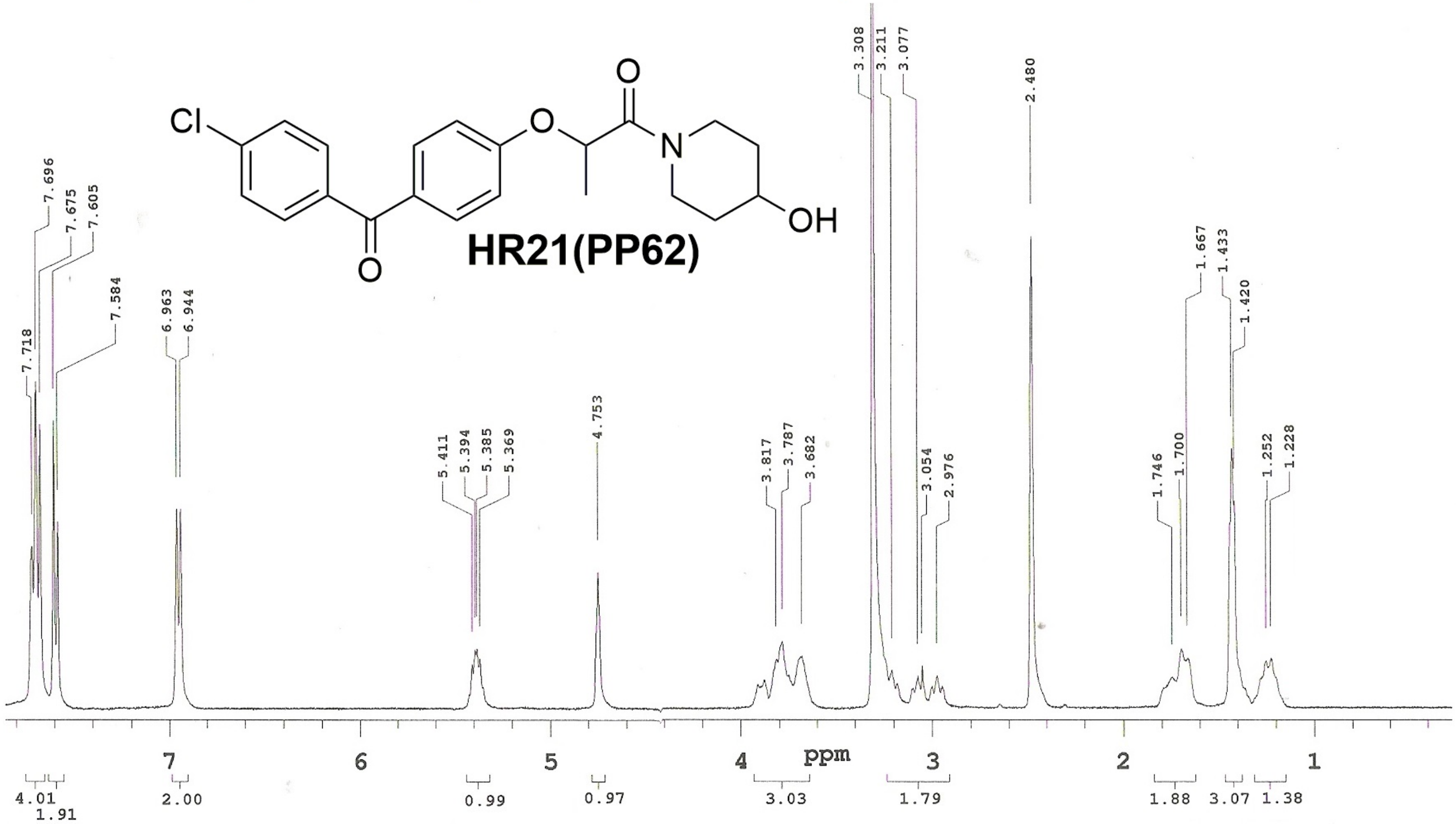

HR21(PP62)

^{1}H-NMR ($CDCl_3$) Varian Mercury 400 Plus

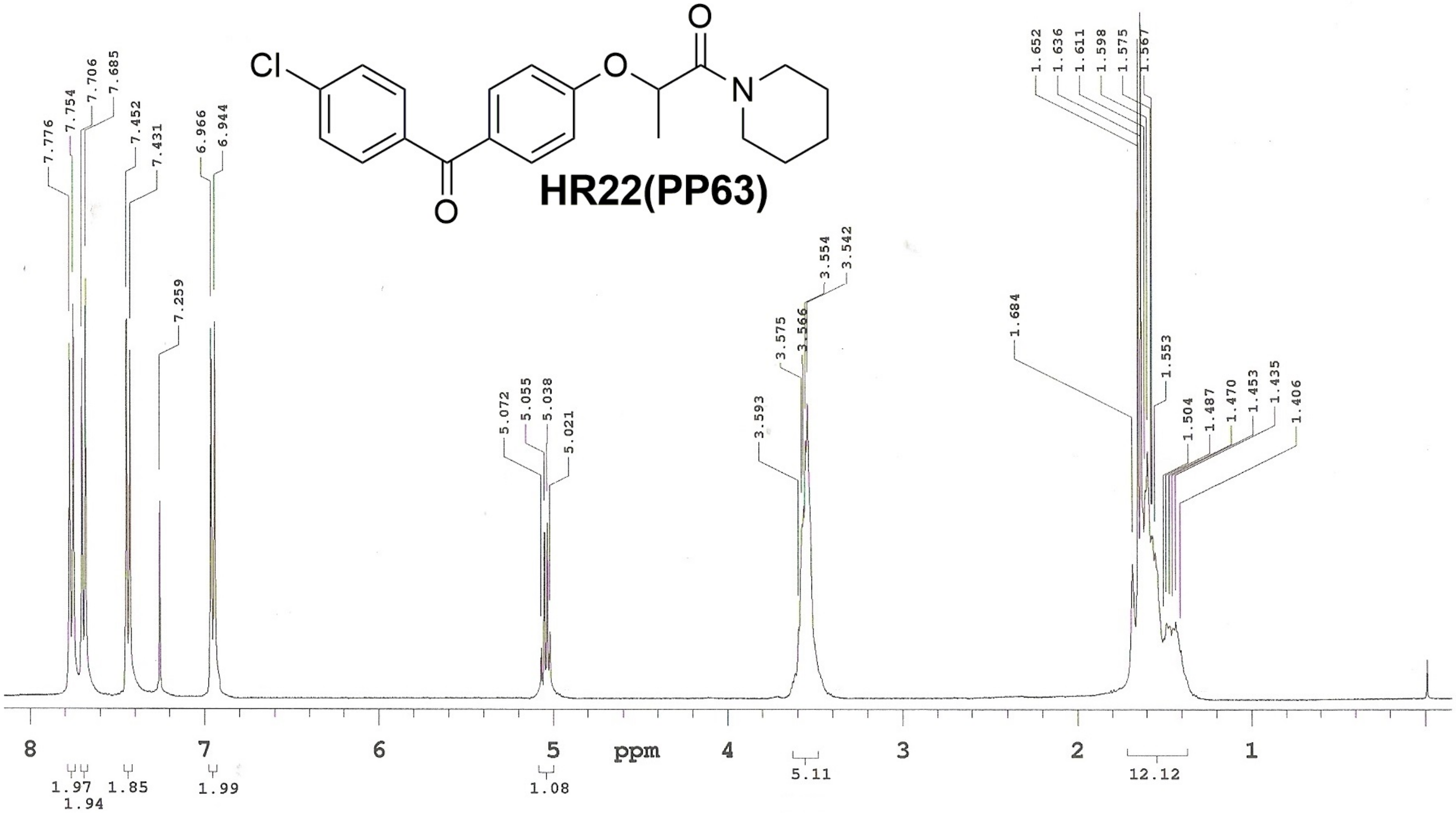

^{1}H-NMR ($CDCl_3$) Varian Mercury 400 Plus

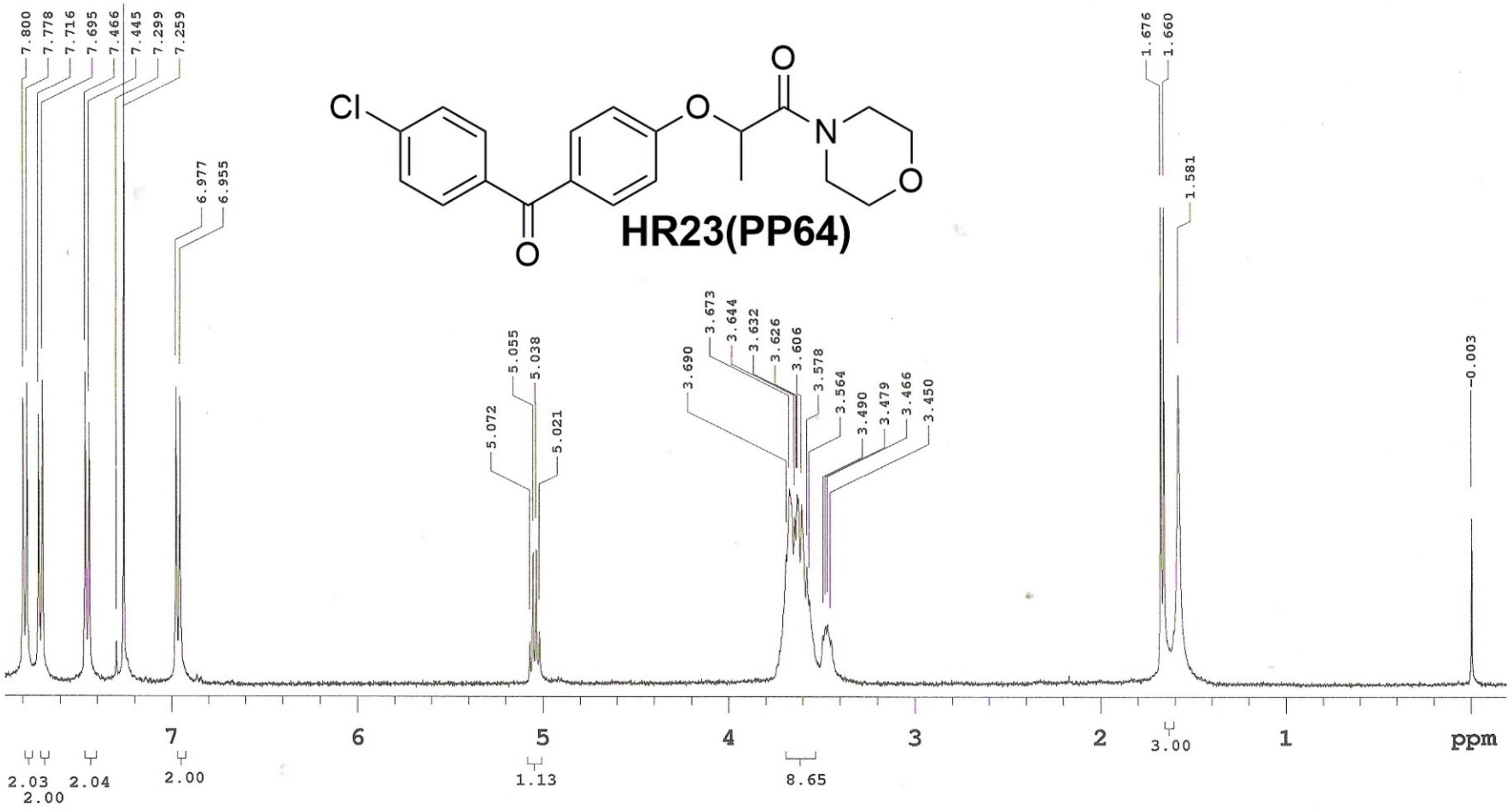

^{1}H-NMR ($CDCl_3$) Varian Mercury 400 Plus

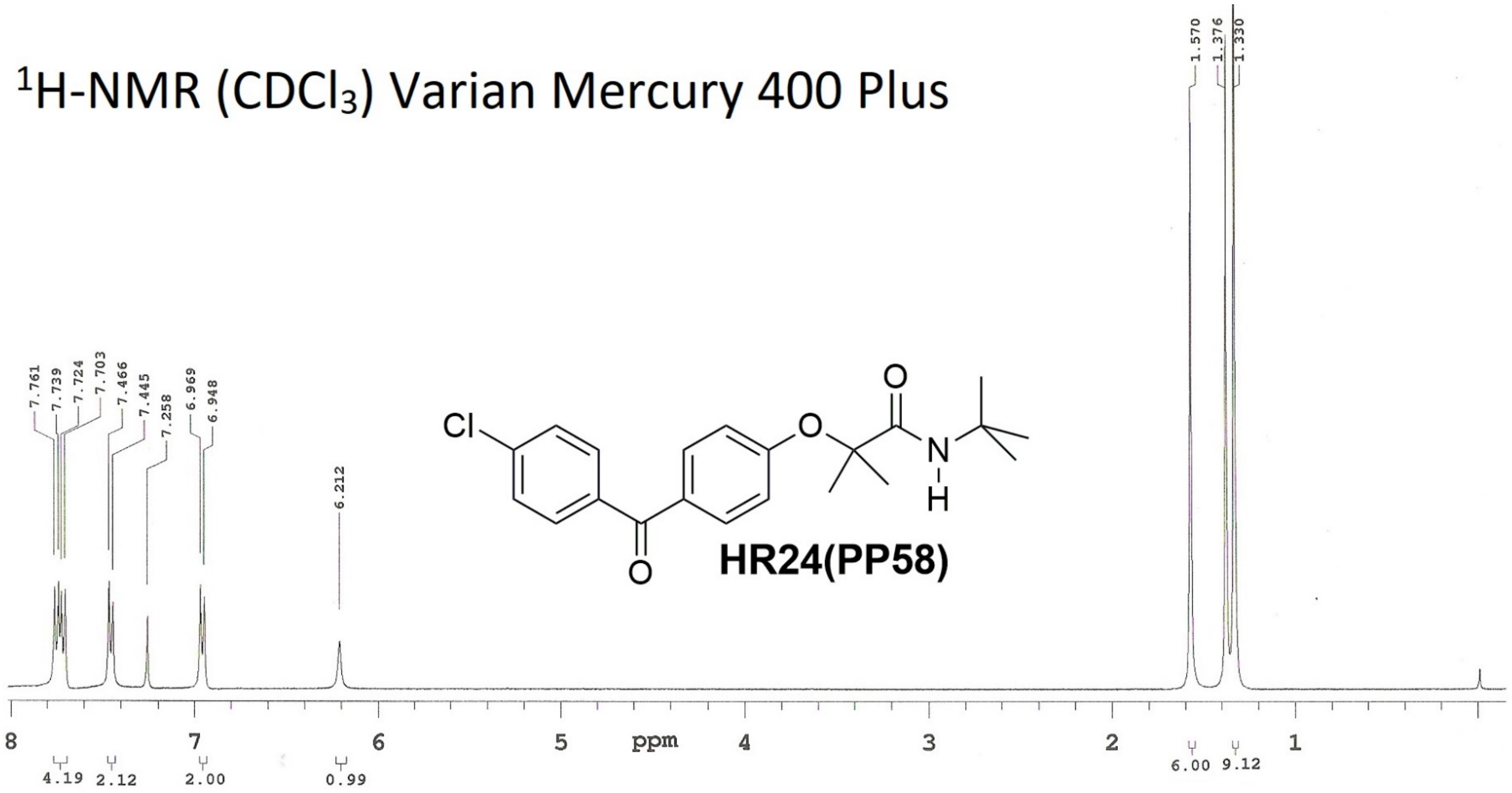

^{1}H-NMR ($CDCl_3$) Varian Mercury 400 Plus

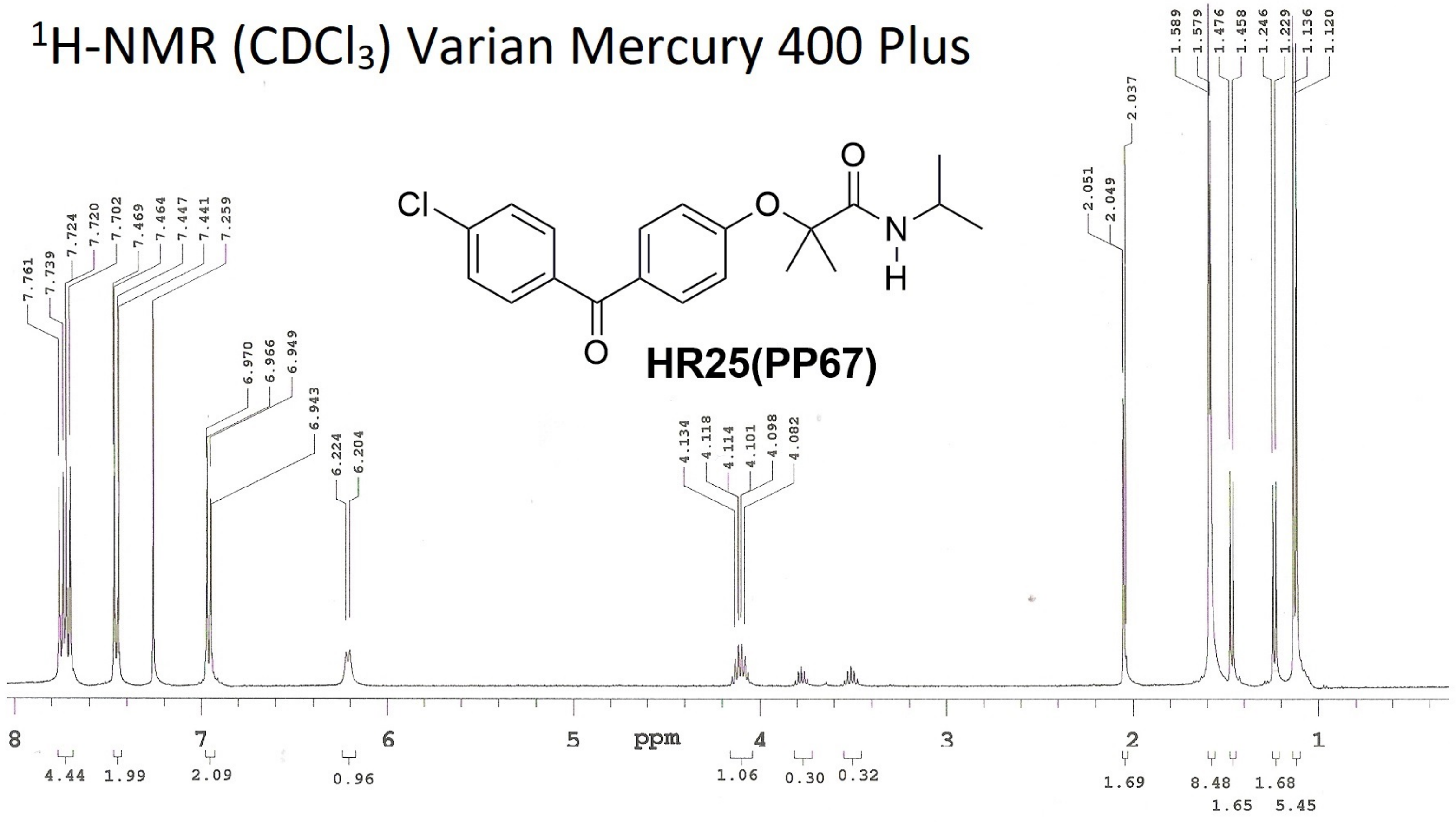

HR25(PP67)

^{1}H-NMR ($CDCl_3$) Varian Mercury 400 Plus

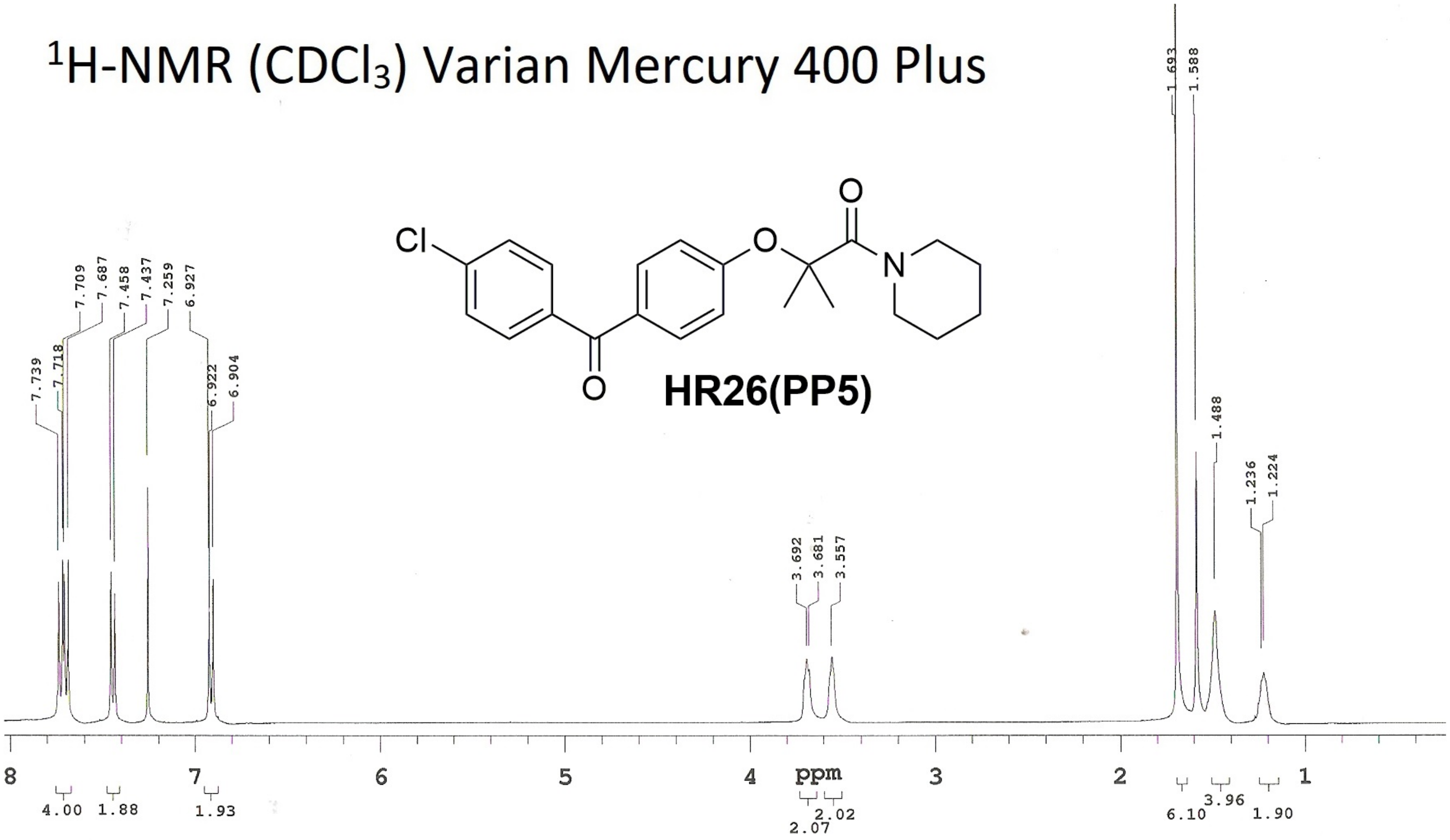

^{1}H-NMR ($CDCl_3$) Varian Mercury 400 Plus

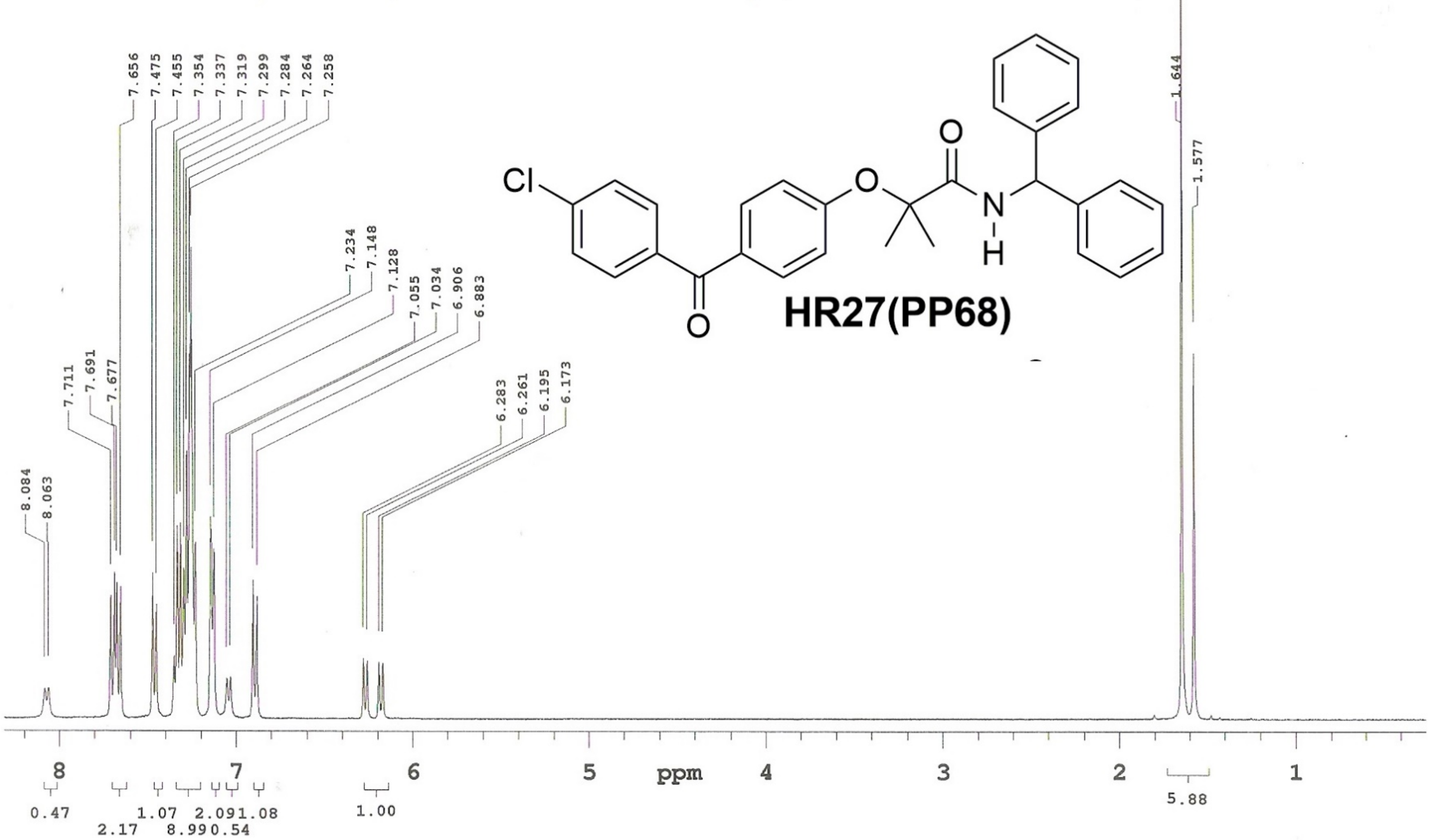

^{1}H-NMR ($CDCl_3$) Varian Mercury 400 Plus

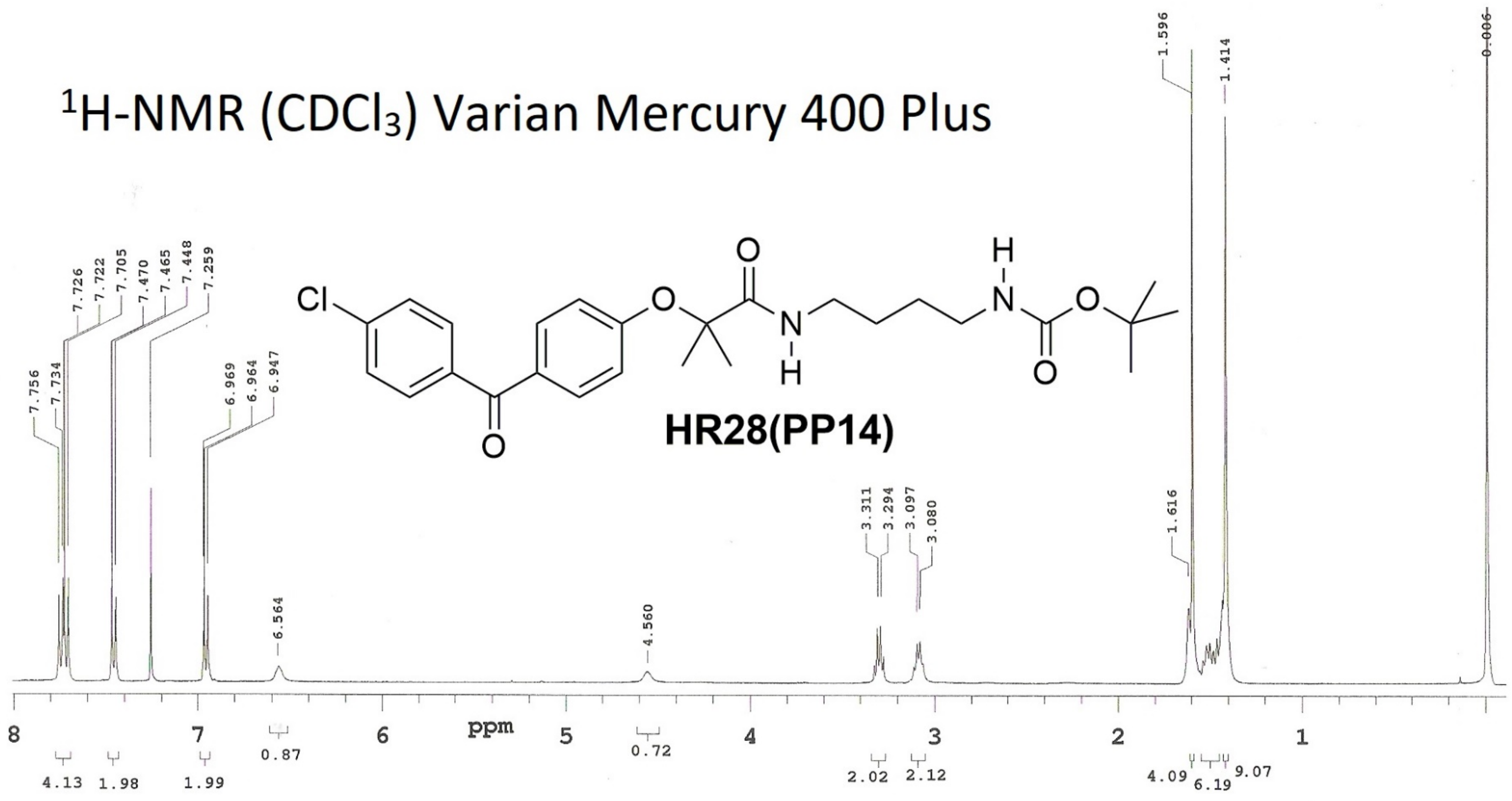

HR28(PP14)

^{1}H-NMR ($CDCl_3$) Varian Mercury 400 Plus

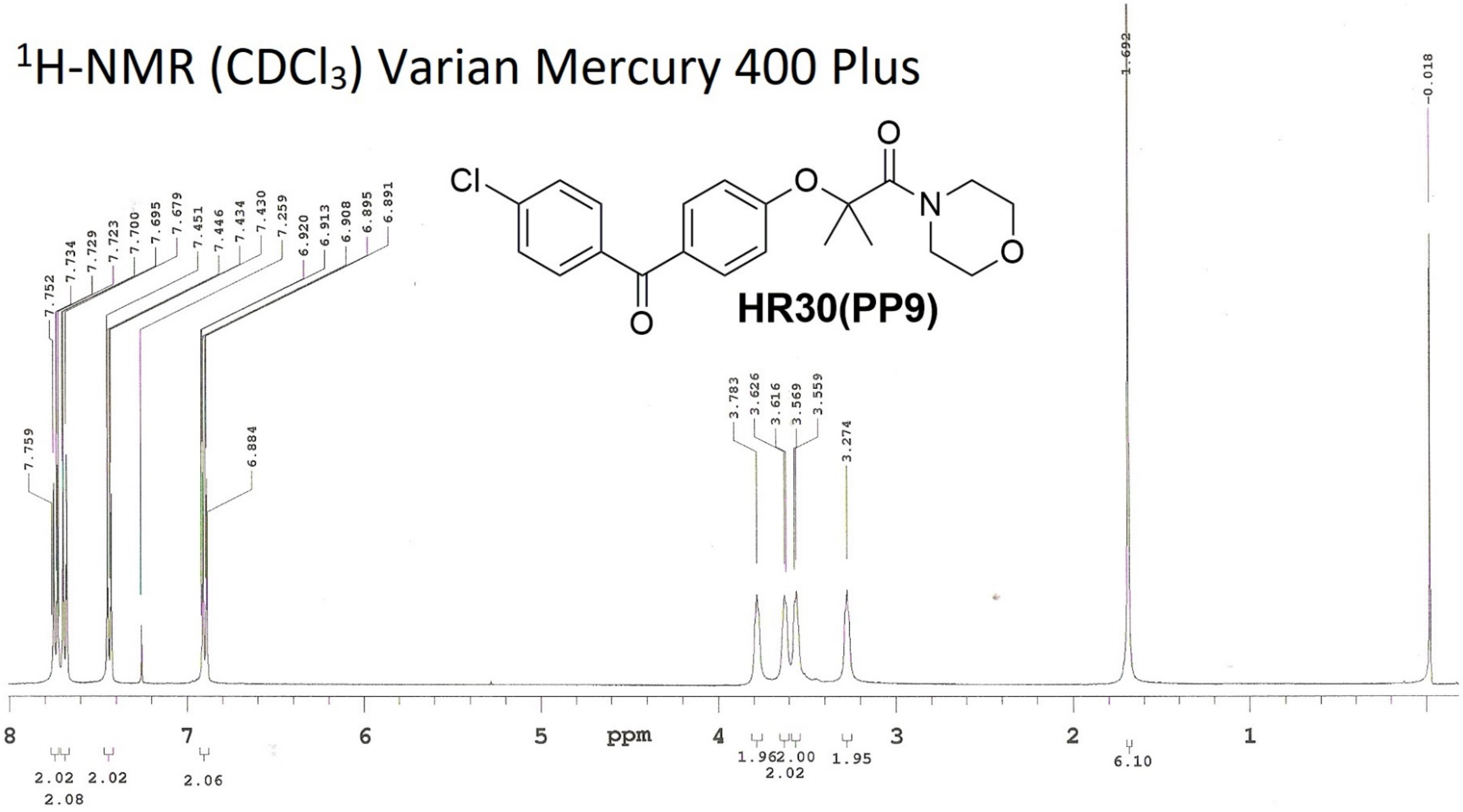

^{1}H-NMR ($CDCl_3$) Varian Mercury 400 Plus

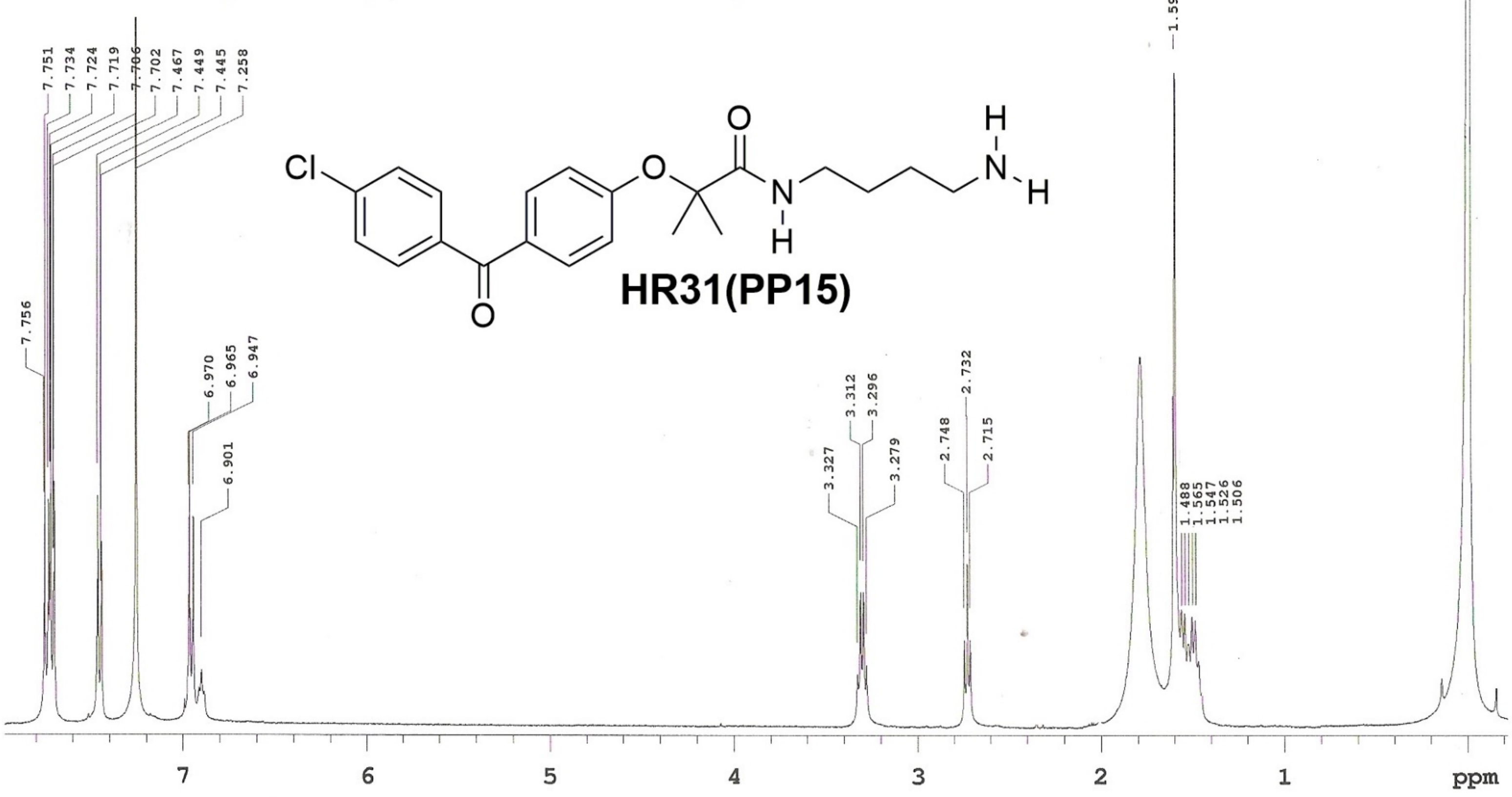

^{1}H-NMR ($CDCl_3$) Varian Mercury 400 Plus

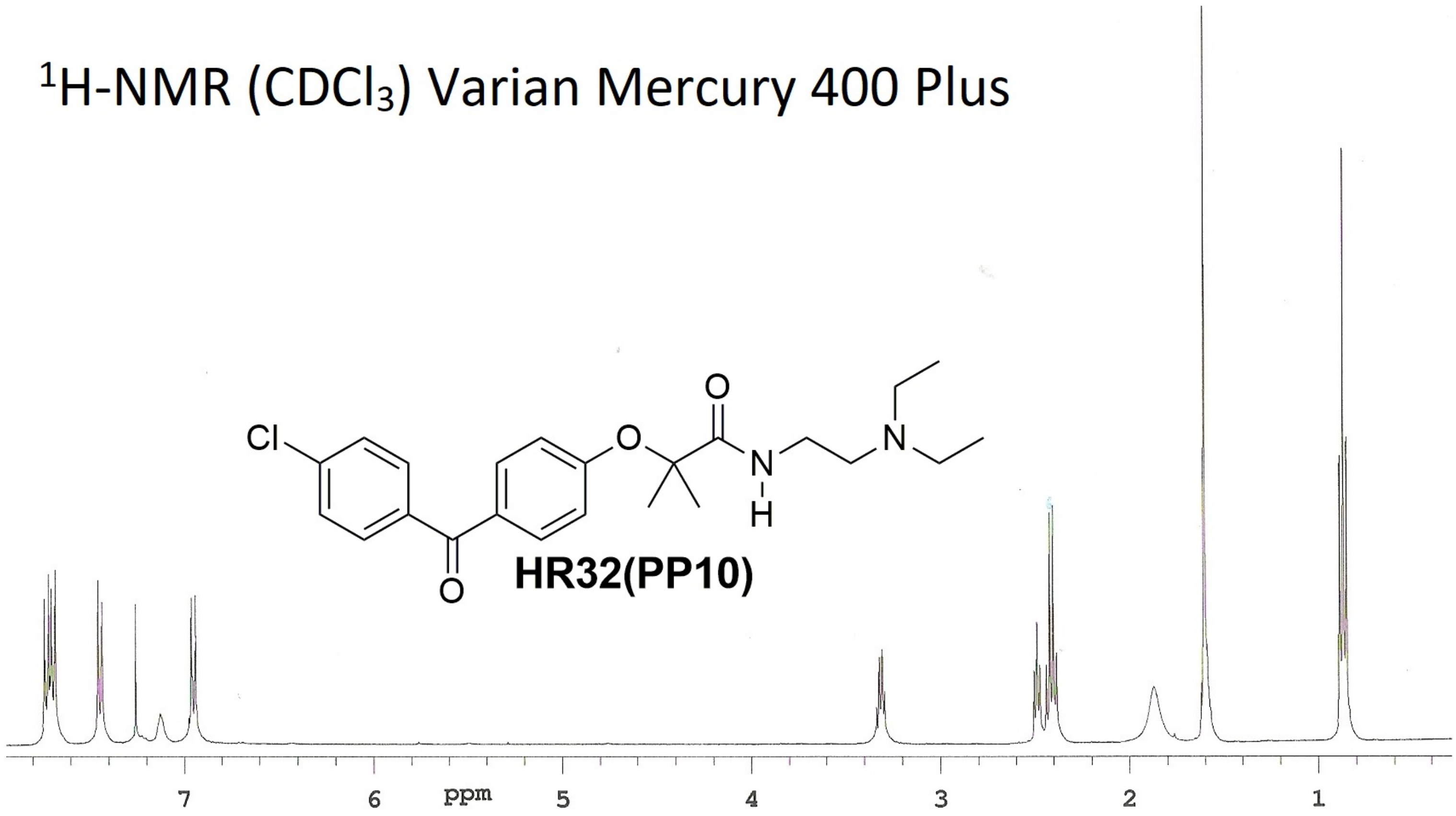

HR32(PP10)

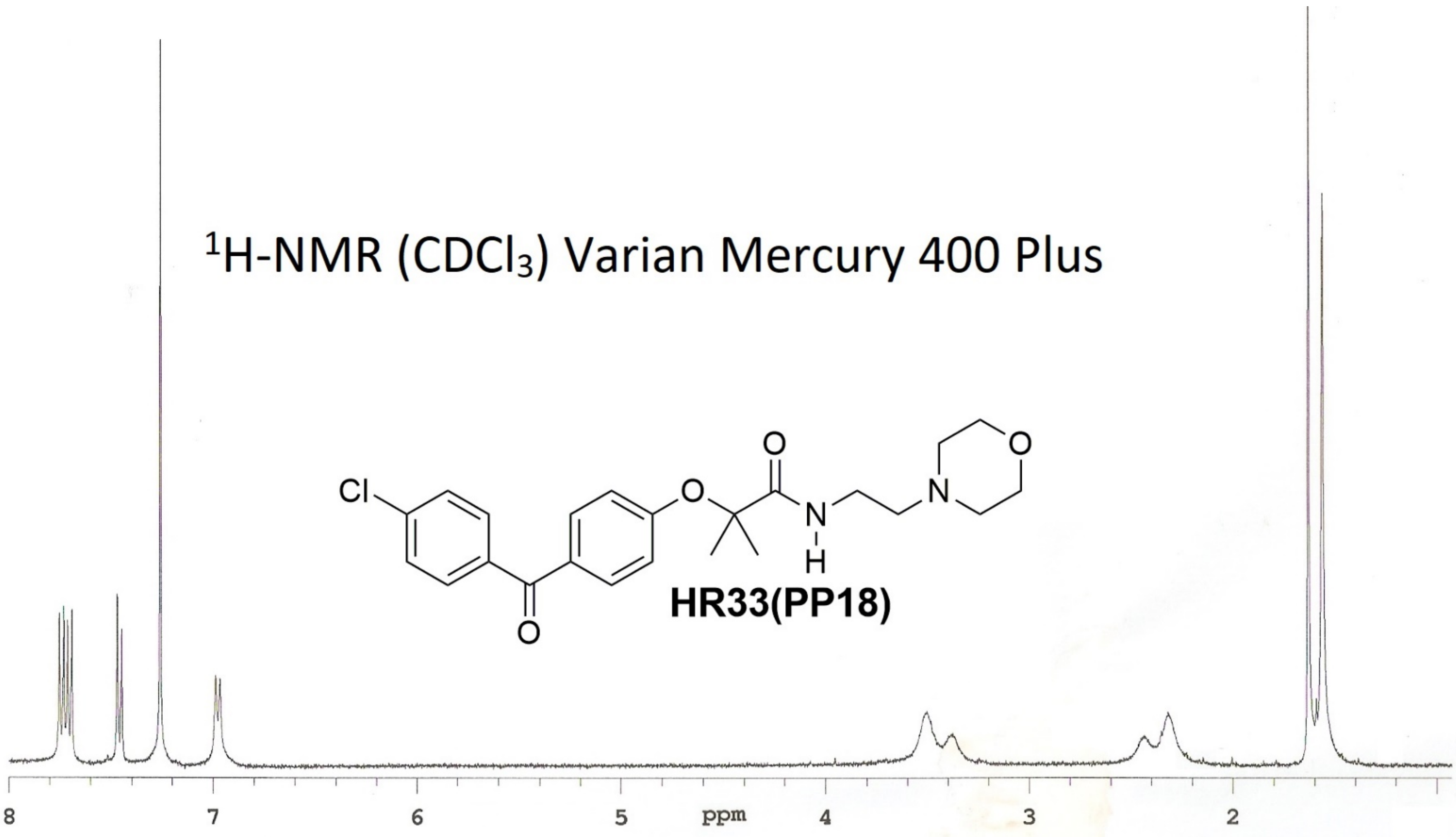

^{1}H-NMR ($CDCl_3$) Varian Mercury 400 Plus
Cl
O
O
O
N
H
N
O
HR33(PP18)
8
7
6
5
ppm
4
3
2

^{1}H-NMR (DMSO-d_6) Varian Mercury 400 Plus

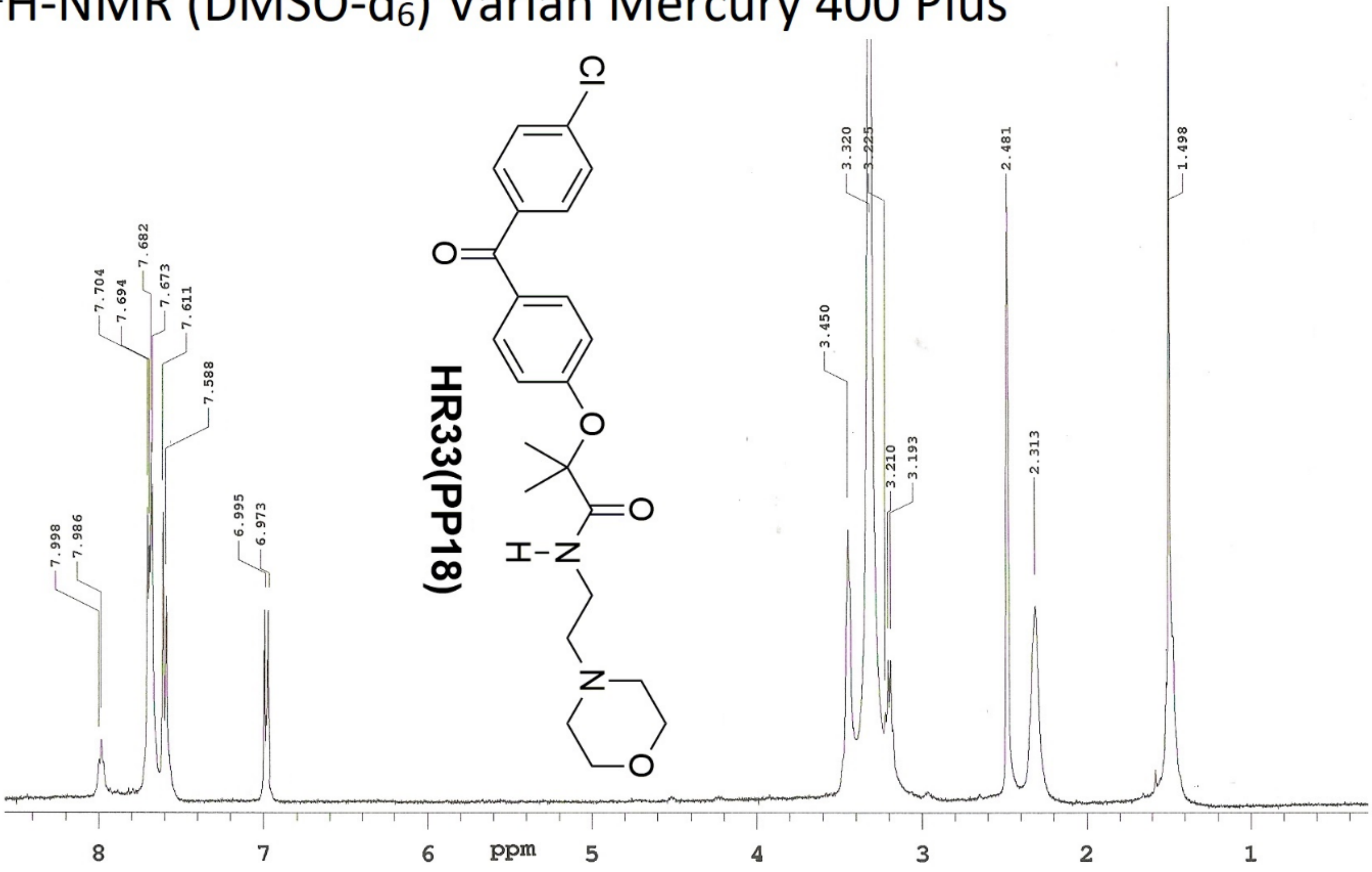

HR33(PP18)

^{13}C-NMR ($CDCl_3$) Varian Mercury 400 Plus

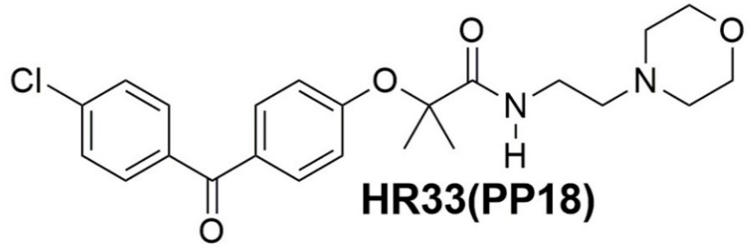

HR33(PP18)

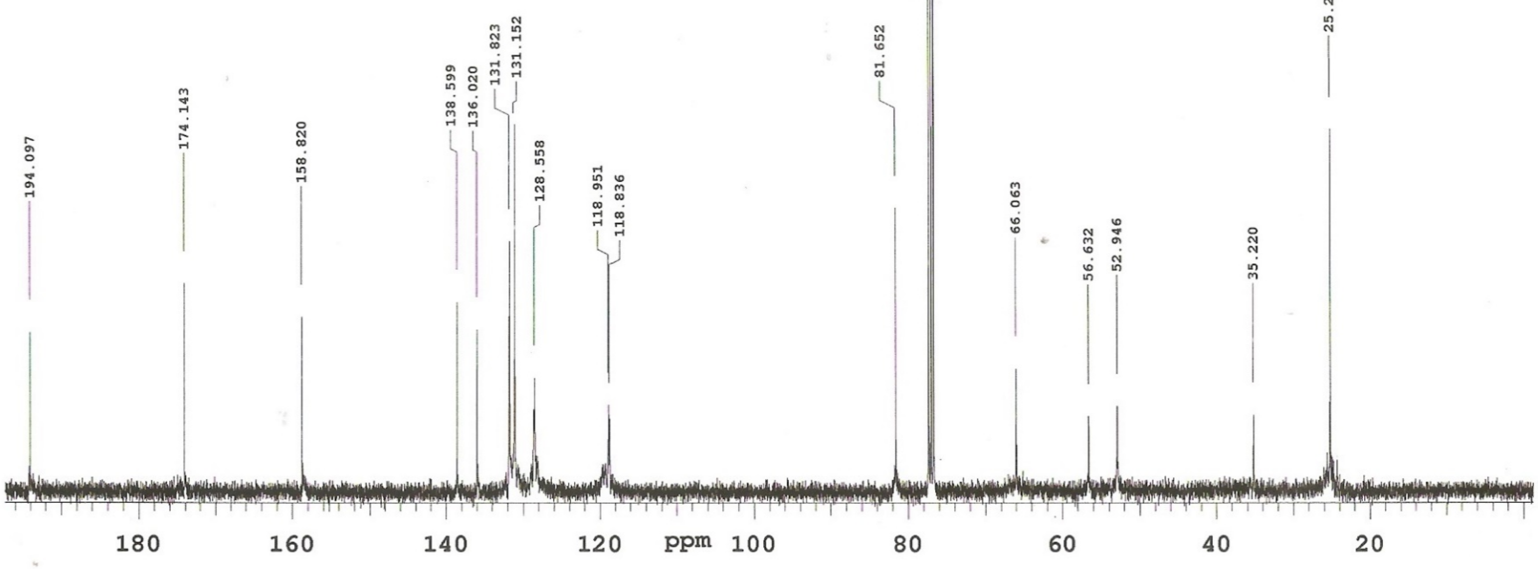

^{1}H-NMR($CDCl_3$) Varian Mercury plus 400 MHz

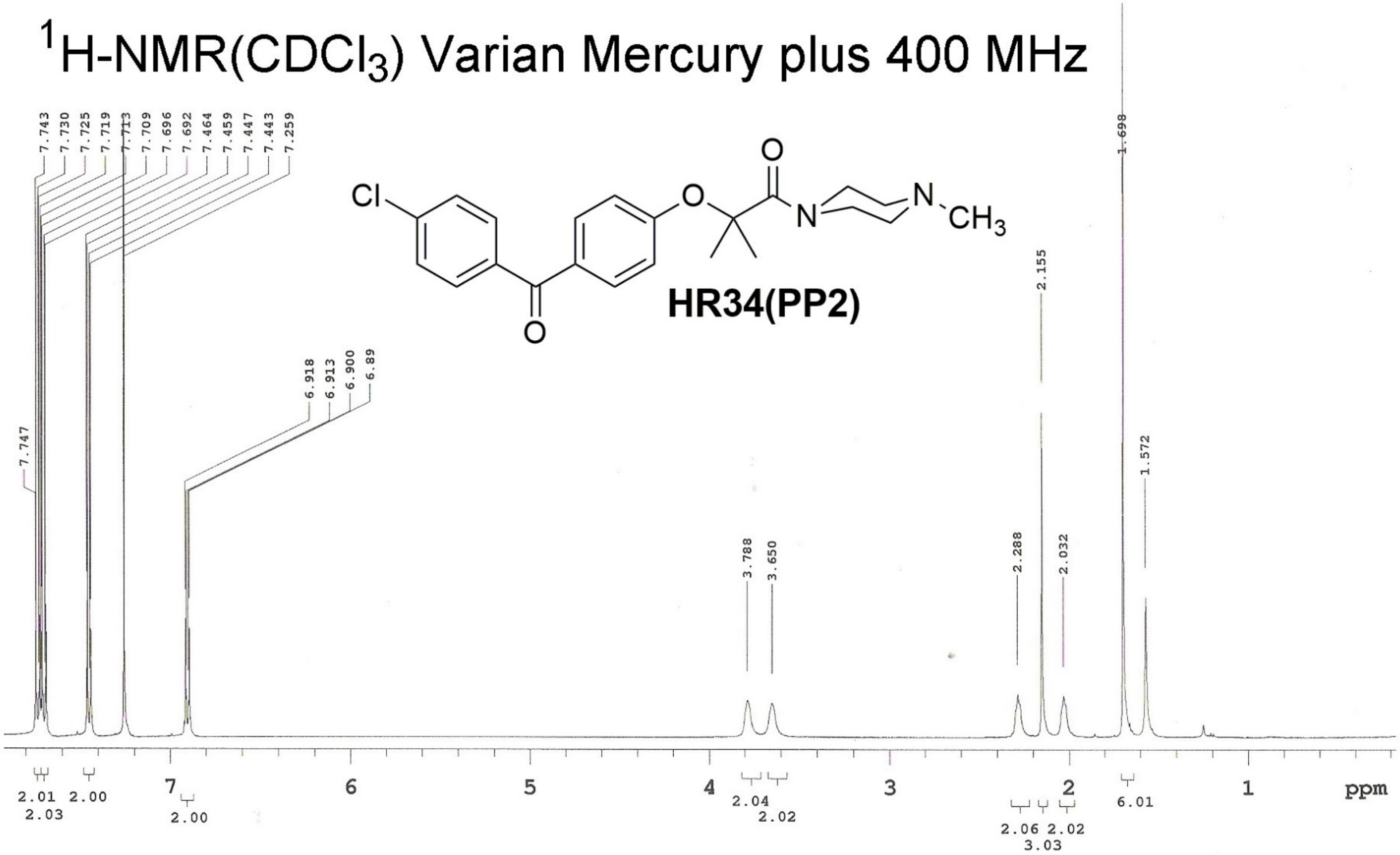

^{1}H-NMR($DMSO-d_6$) Varian Mercury plus 400 MHz

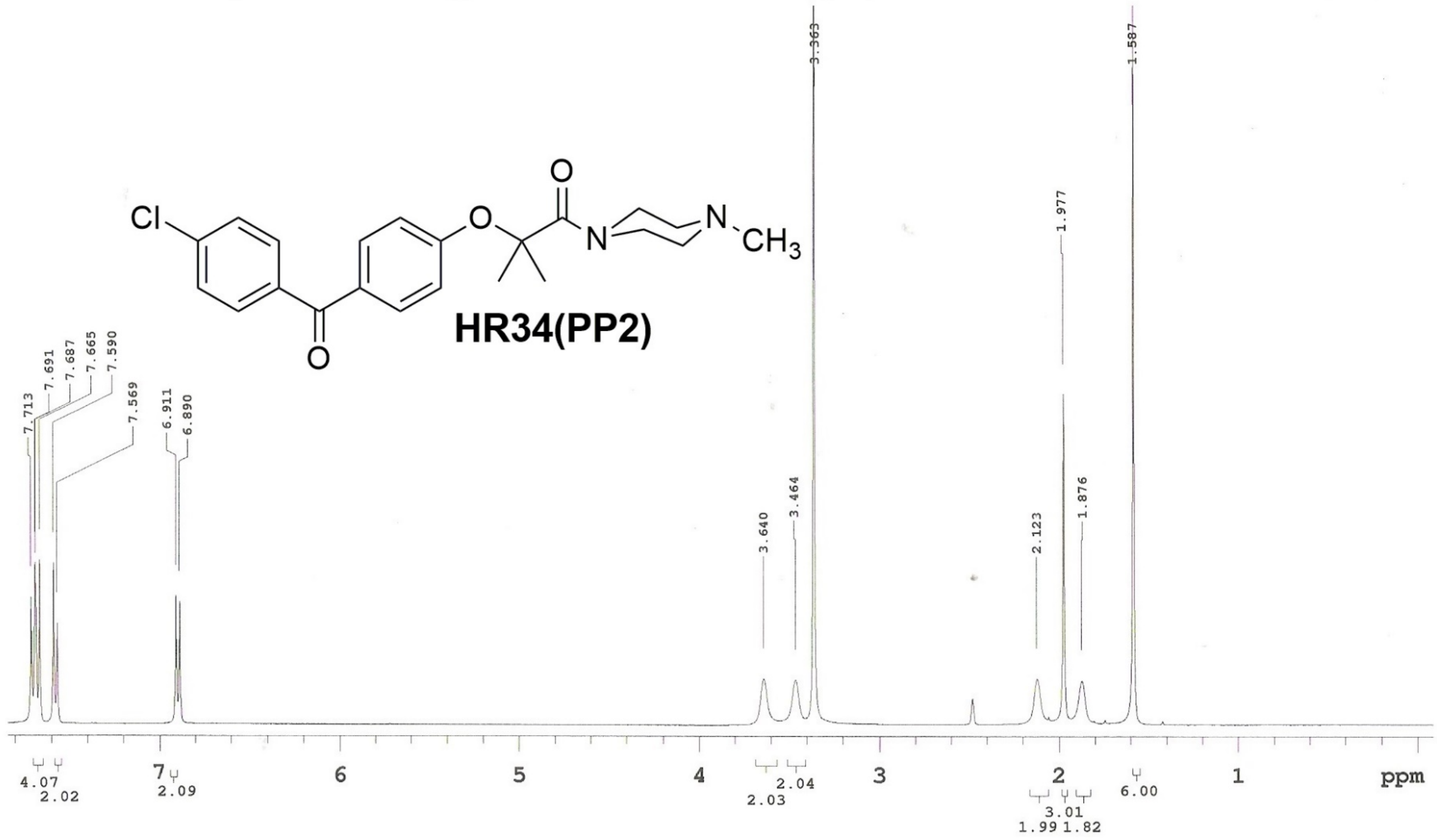

HR34(PP2)

^{13}C-NMR(DMSO-d_6) Varian Mercury plus 400 MHz

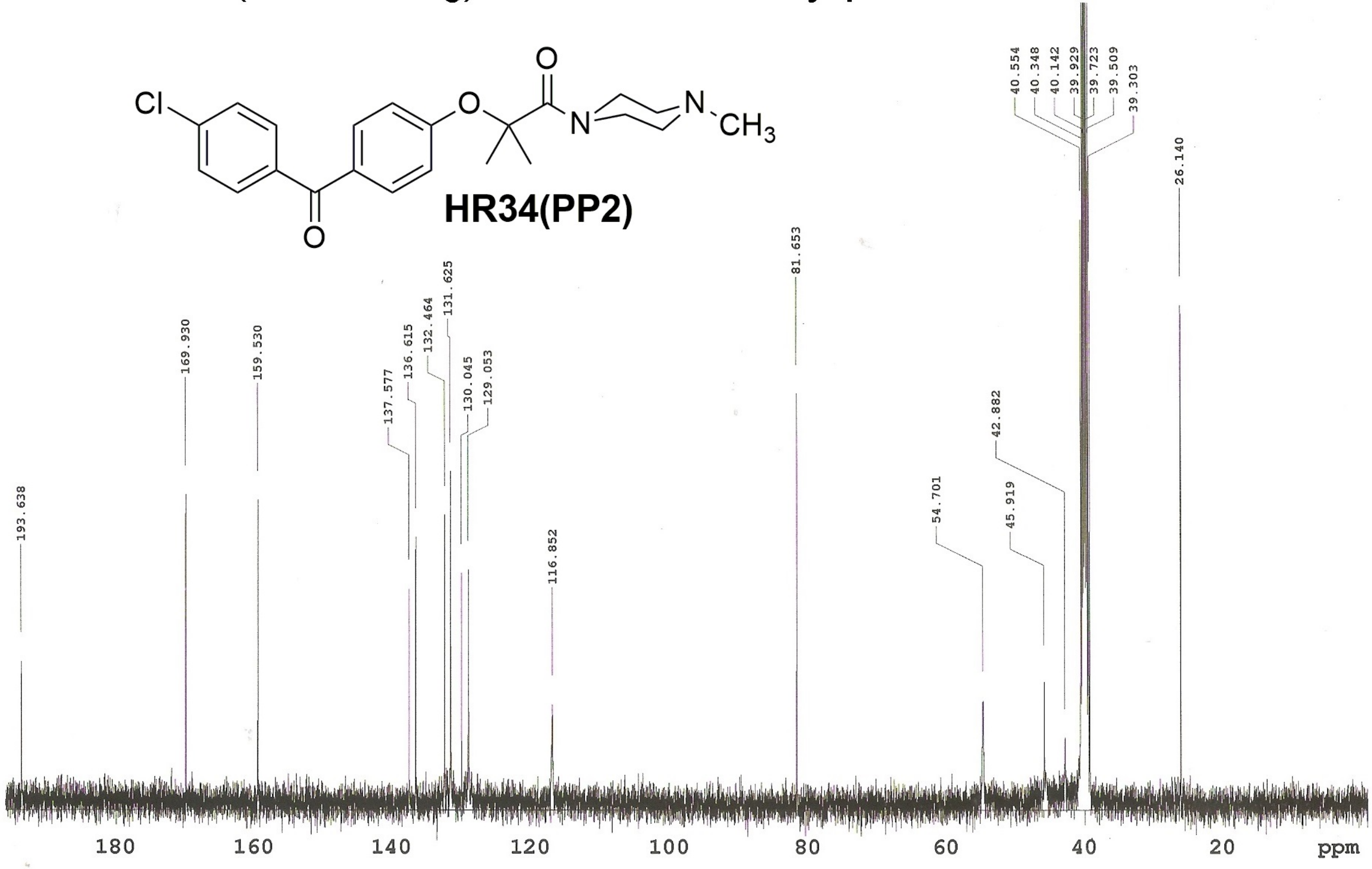

HR34(PP2)

^{1}H-NMR($CDCl_3$) Varian Mercury plus 400 MHz

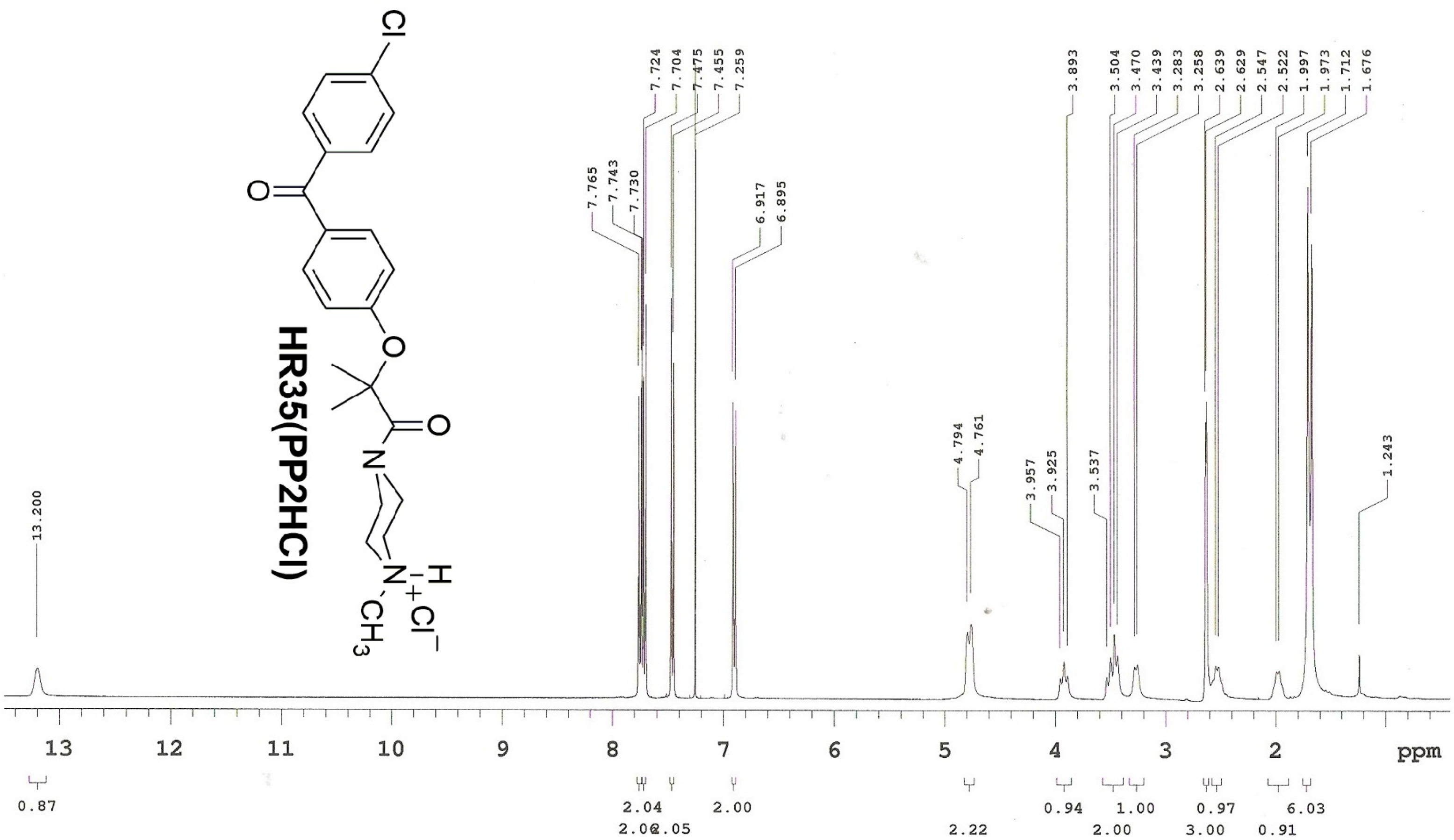

^{1}H-NMR($CDCl_3$) Varian Mercury plus 400 MHz

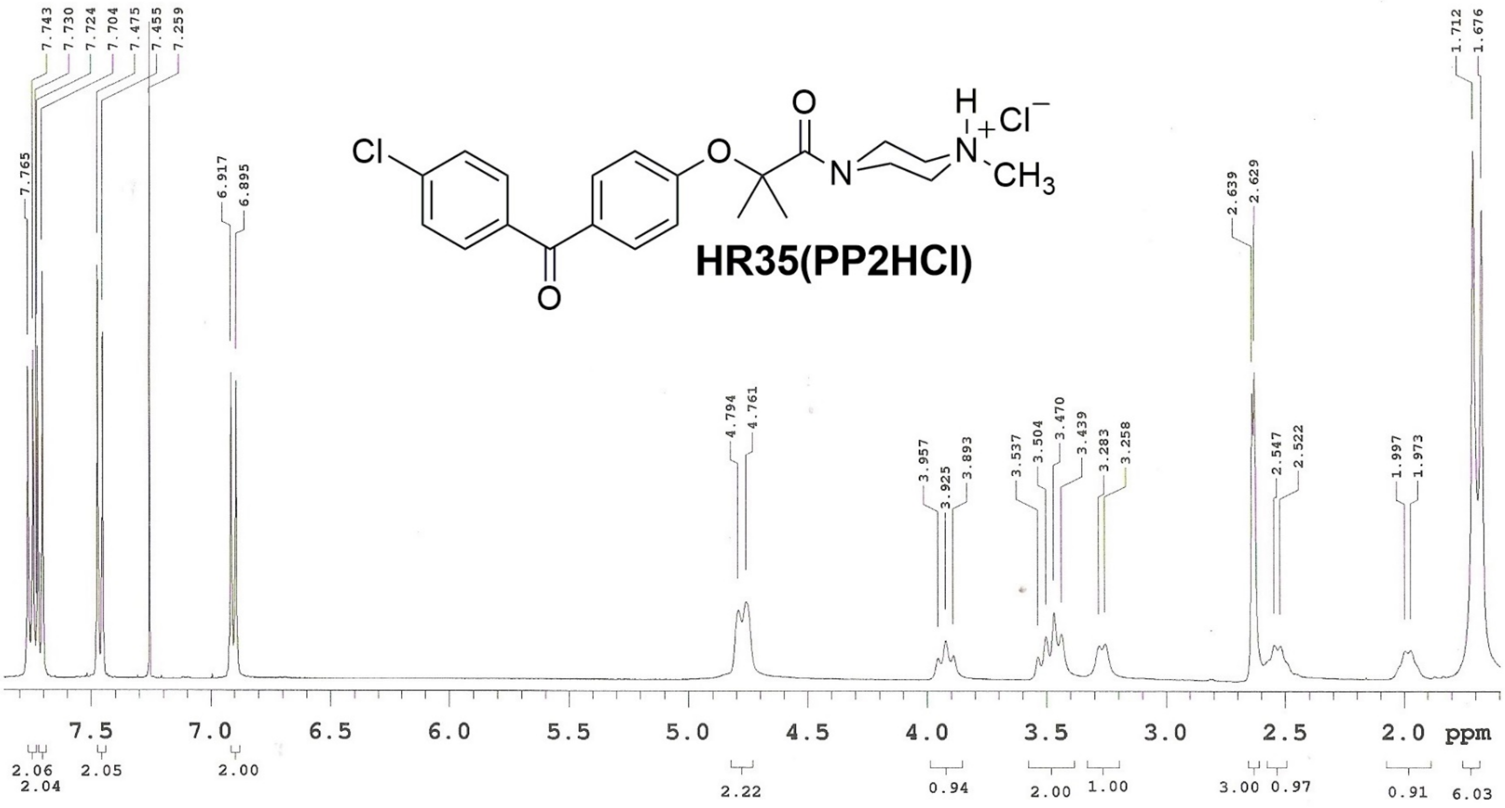

^{1}H-NMR(DMSO-d_6) Varian Mercury plus 400 MHz

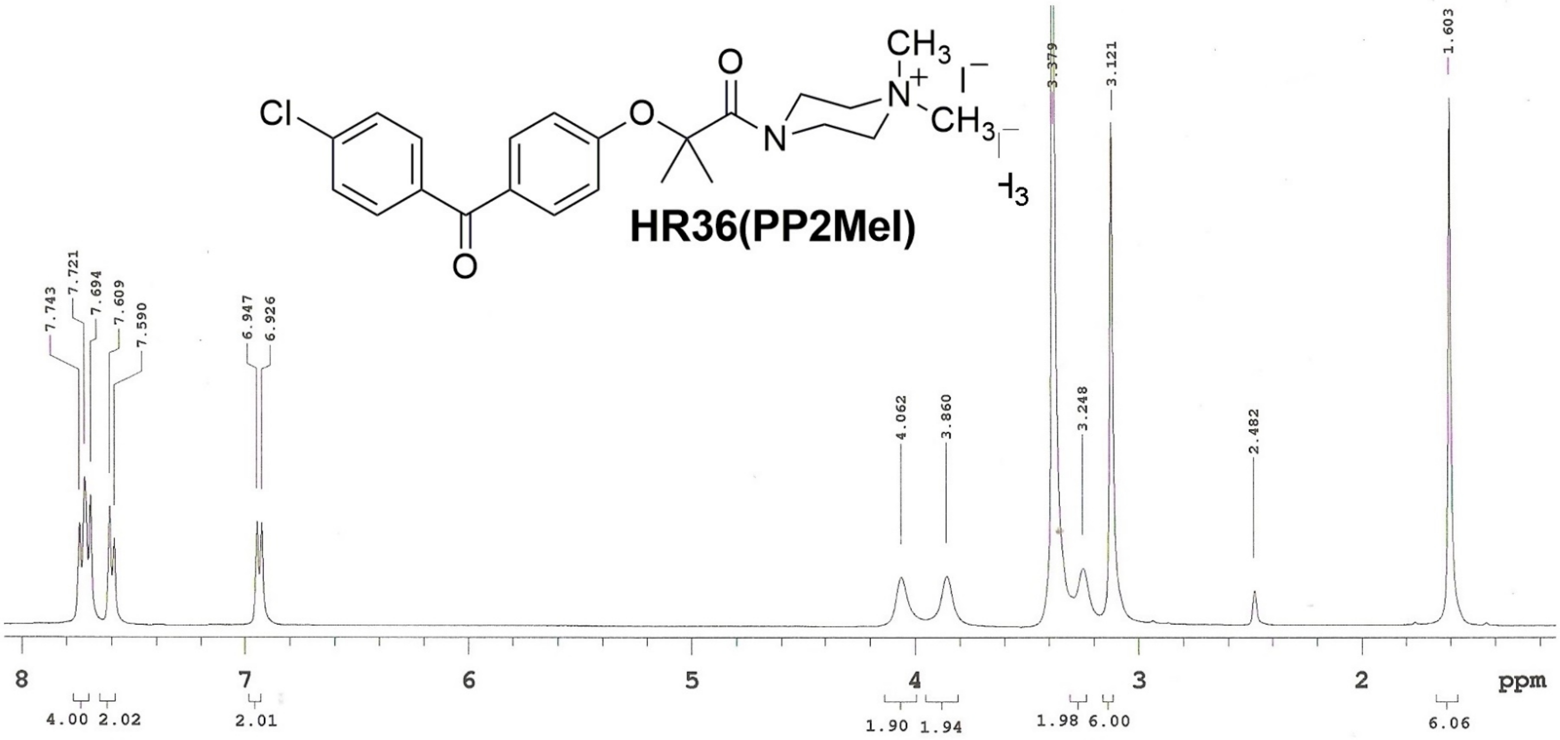

HR36(PP2MeI)

^{13}C-NMR(DMSO-d_6) Varian Mercury plus 400 MHz

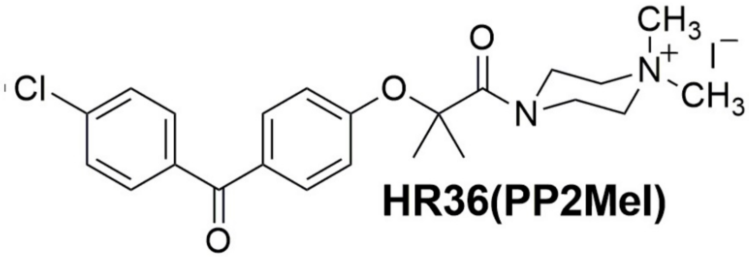

HR36(PP2MeI)

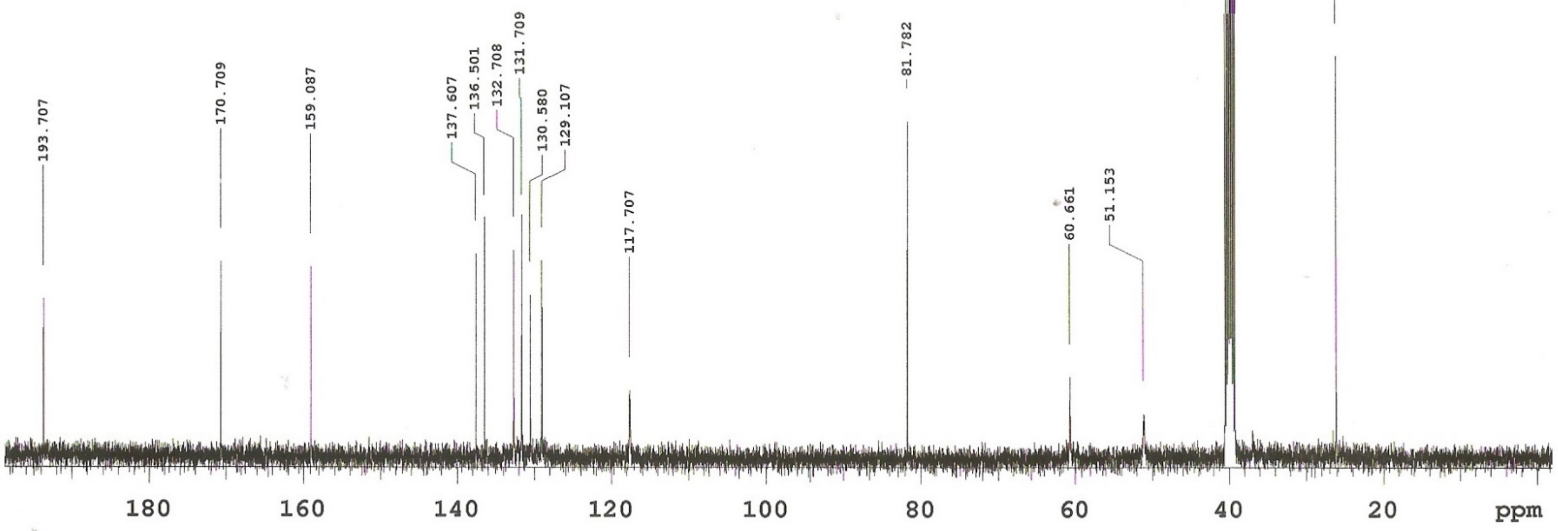

^{1}H-NMR($CDCl_3$) Varian Mercury plus 400 MHz

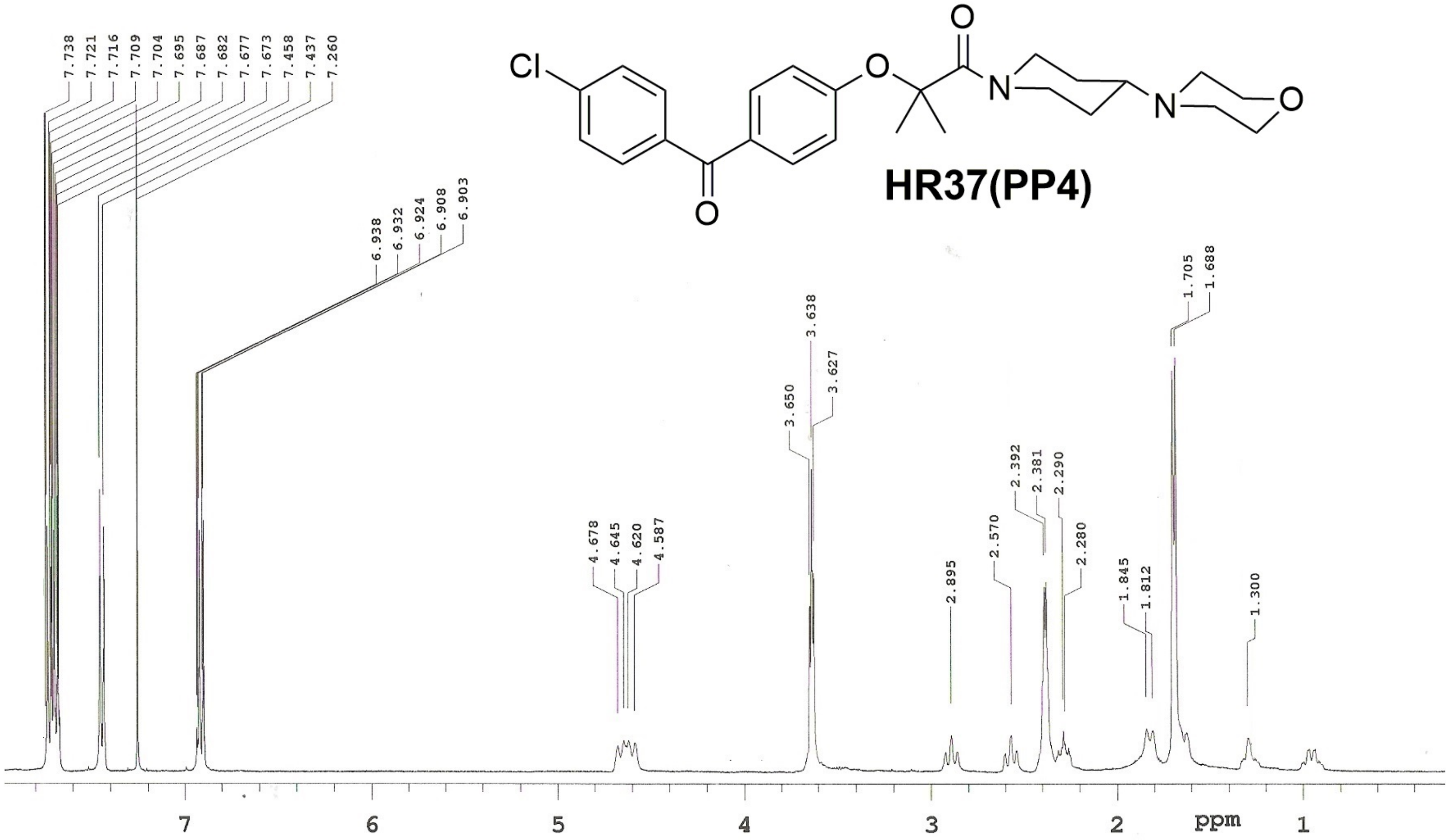

HR37(PP4)

^{1}H-NMR($CDCl_3$) Varian Mercury plus 400 MHz

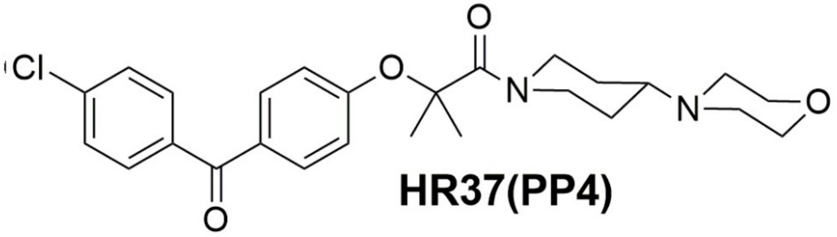

HR37(PP4)

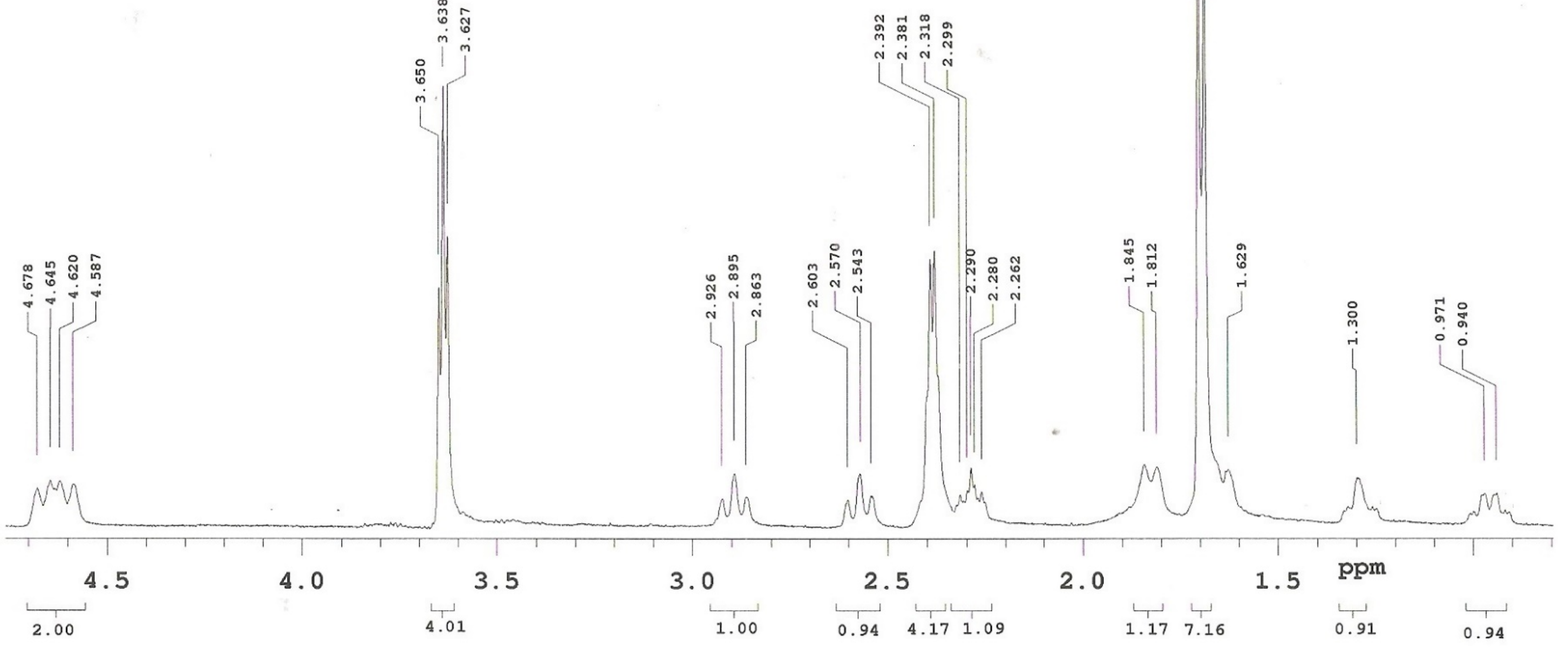

^{1}H-NMR($CDCl_3$) Varian Mercury plus 400 MHz

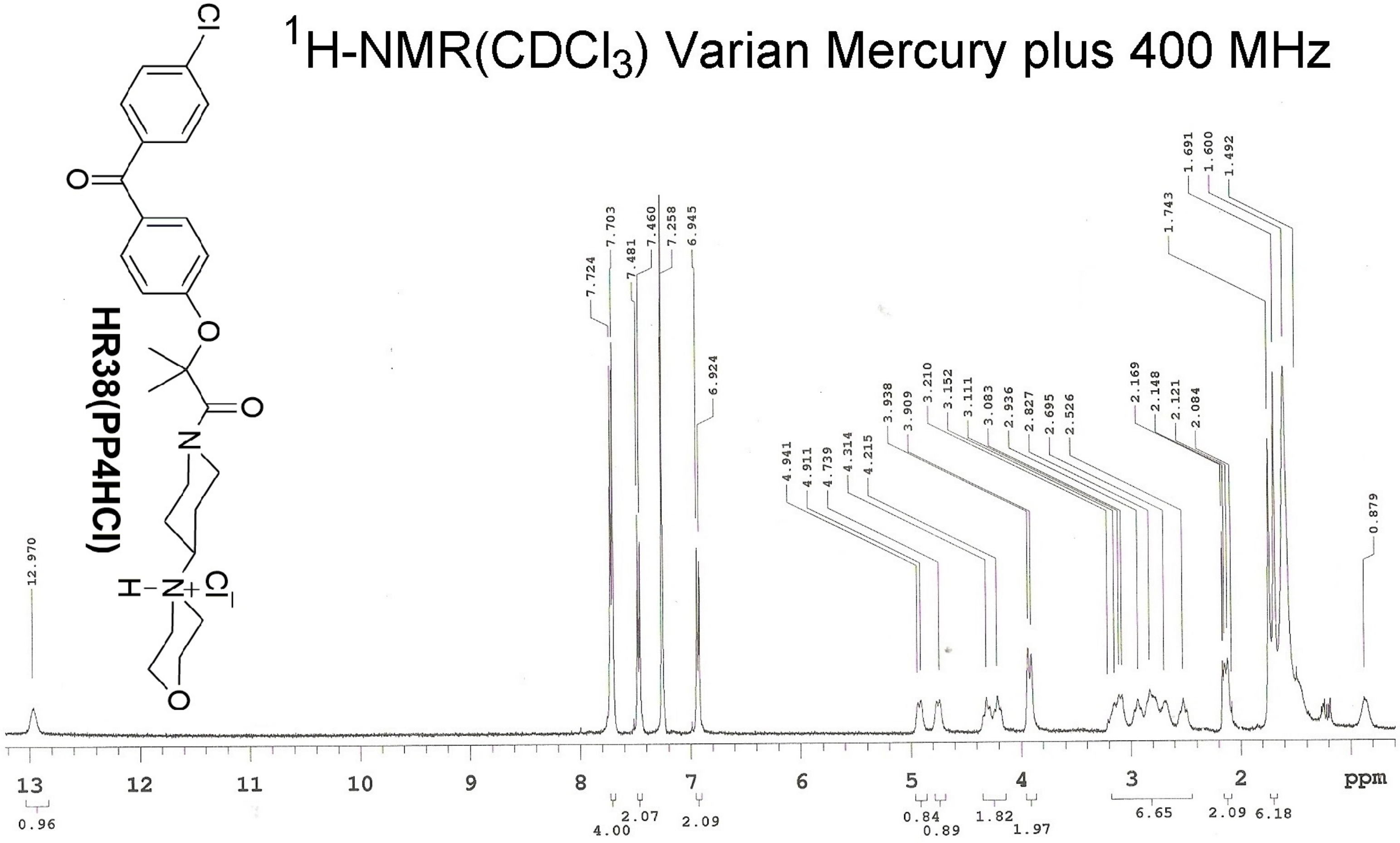

HR38(PP4HCl)

^{1}H-NMR ($CDCl_3$) Varian Mercury 400 Plus

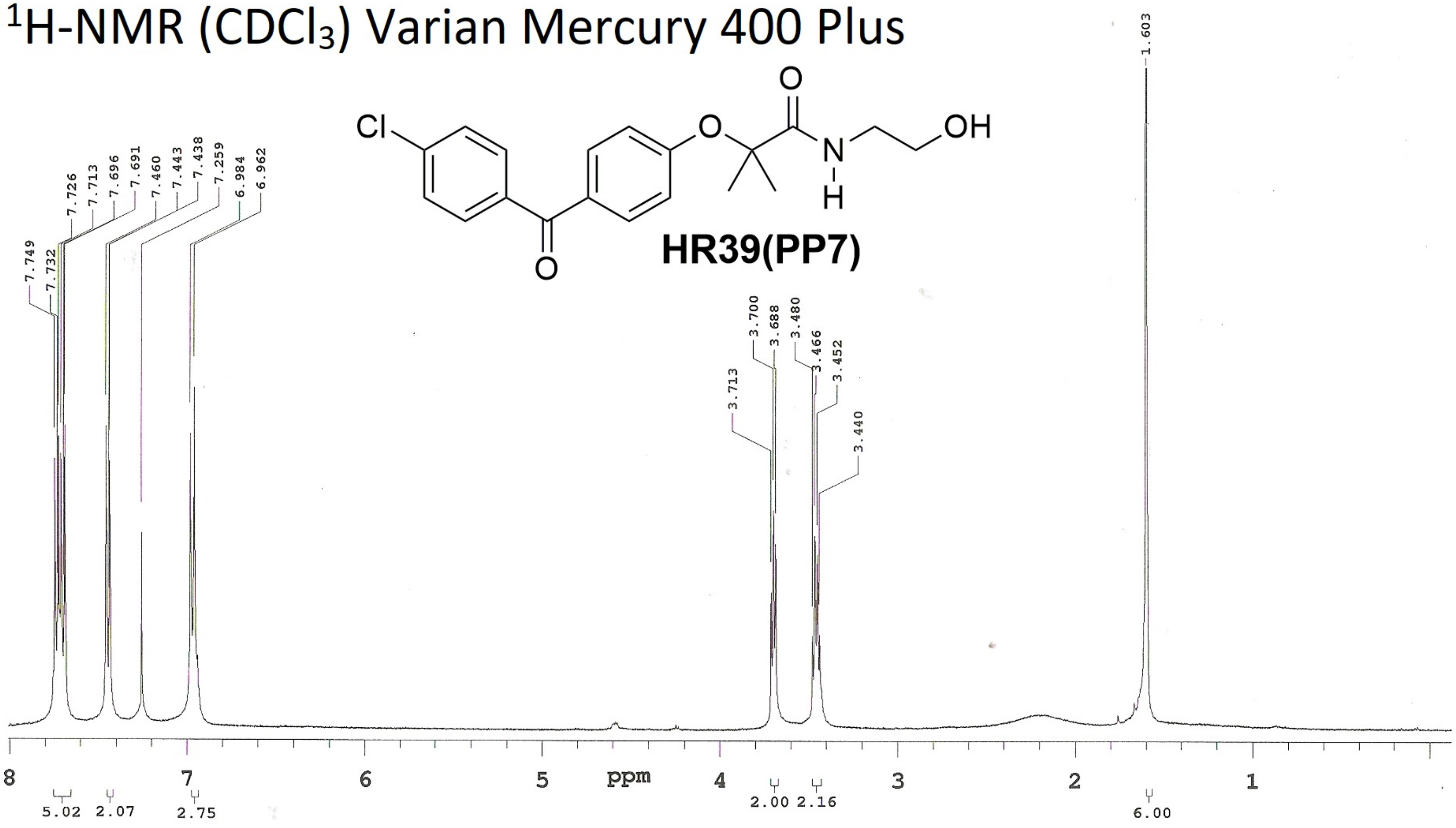

^{1}H-NMR(DMSO-d_6) Varian Mercury plus 400 MHz

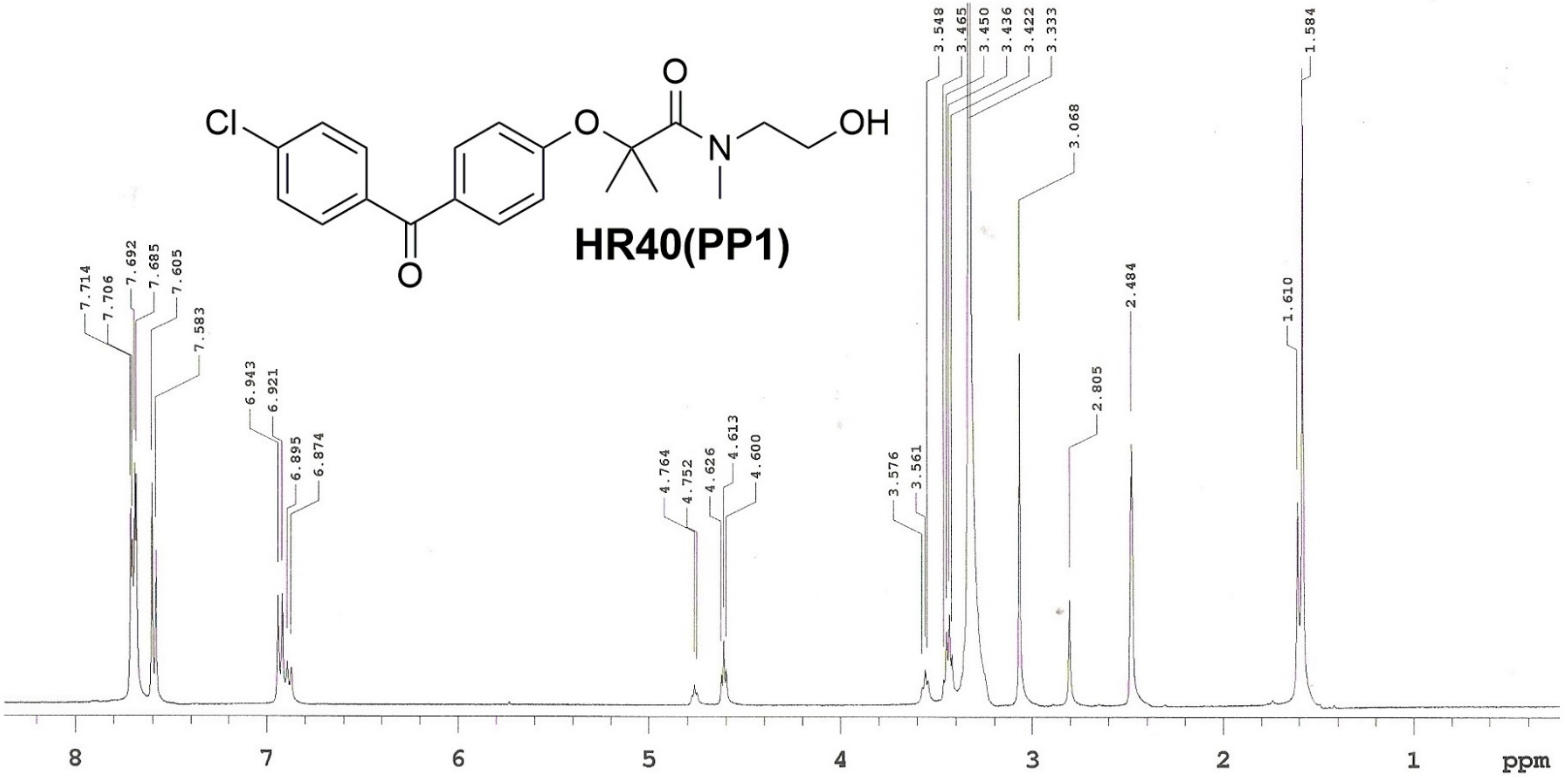

HR40(PP1)

^{1}H-NMR($CDCl_3$) Varian Mercury plus 400 MHz

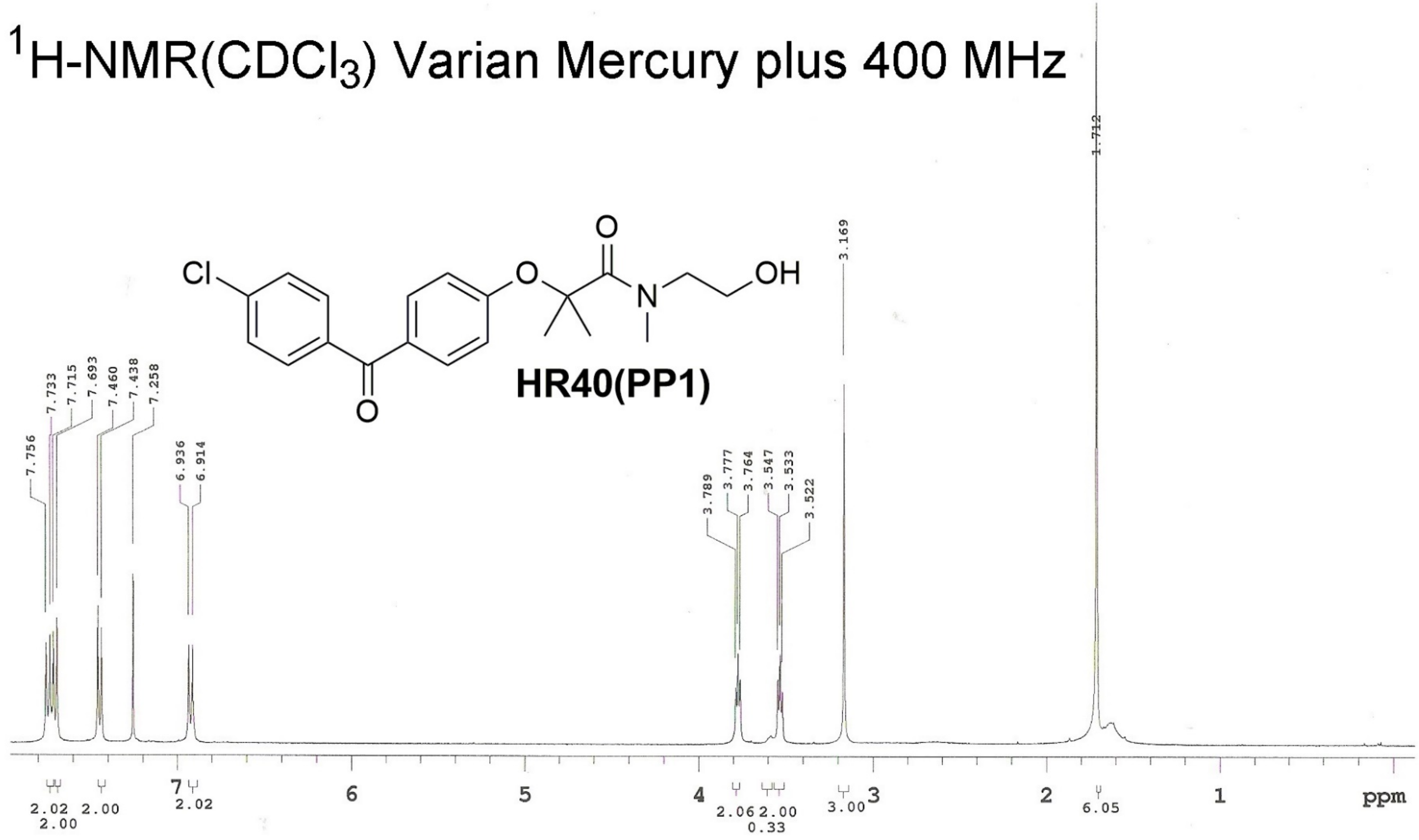

^{13}C-NMR($CDCl_3$) Varian Mercury plus 400 MHz

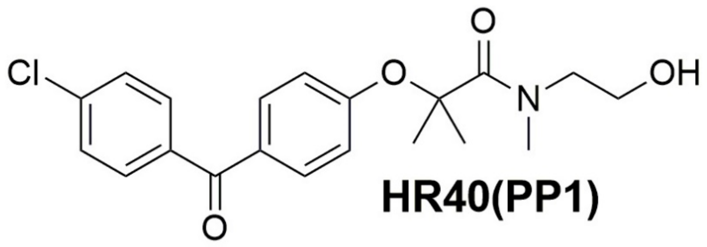

HR40(PP1)

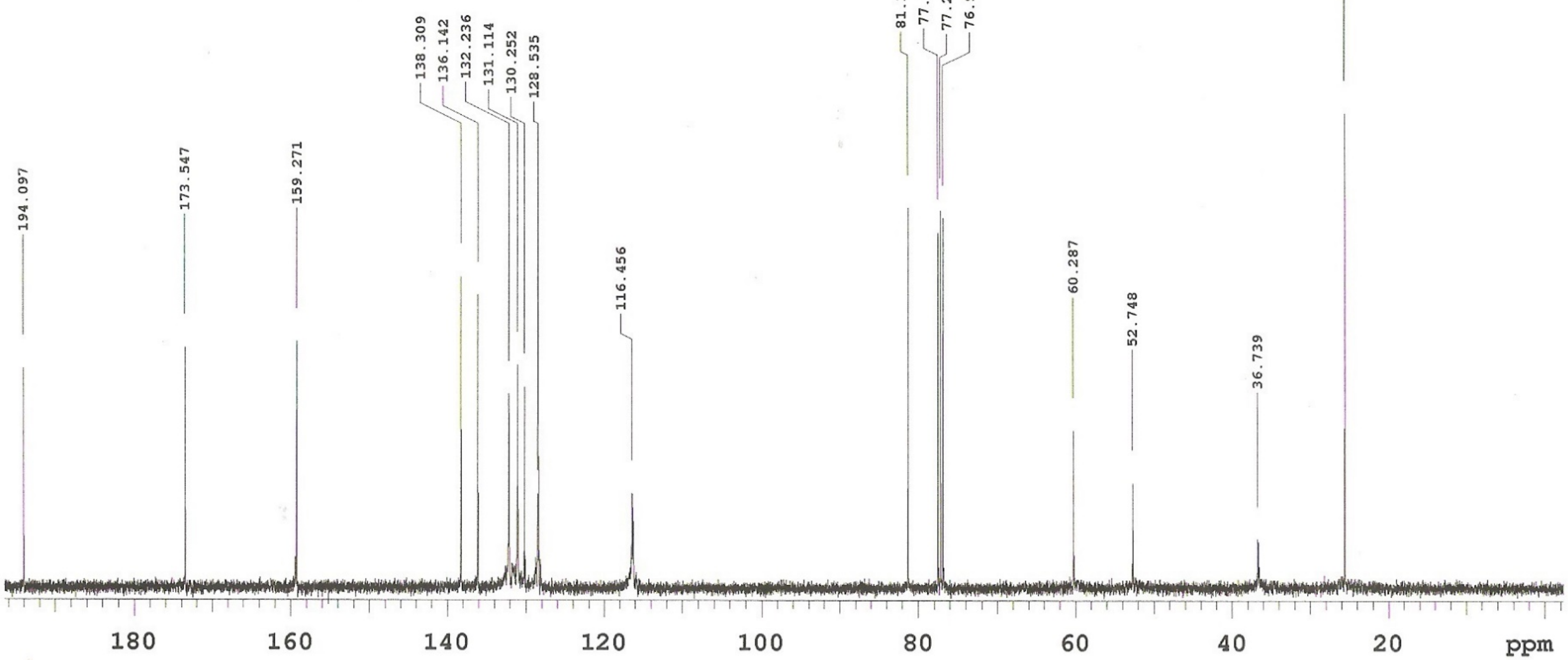

^{1}H-NMR ($CDCl_3$) Varian Mercury 400 Plus

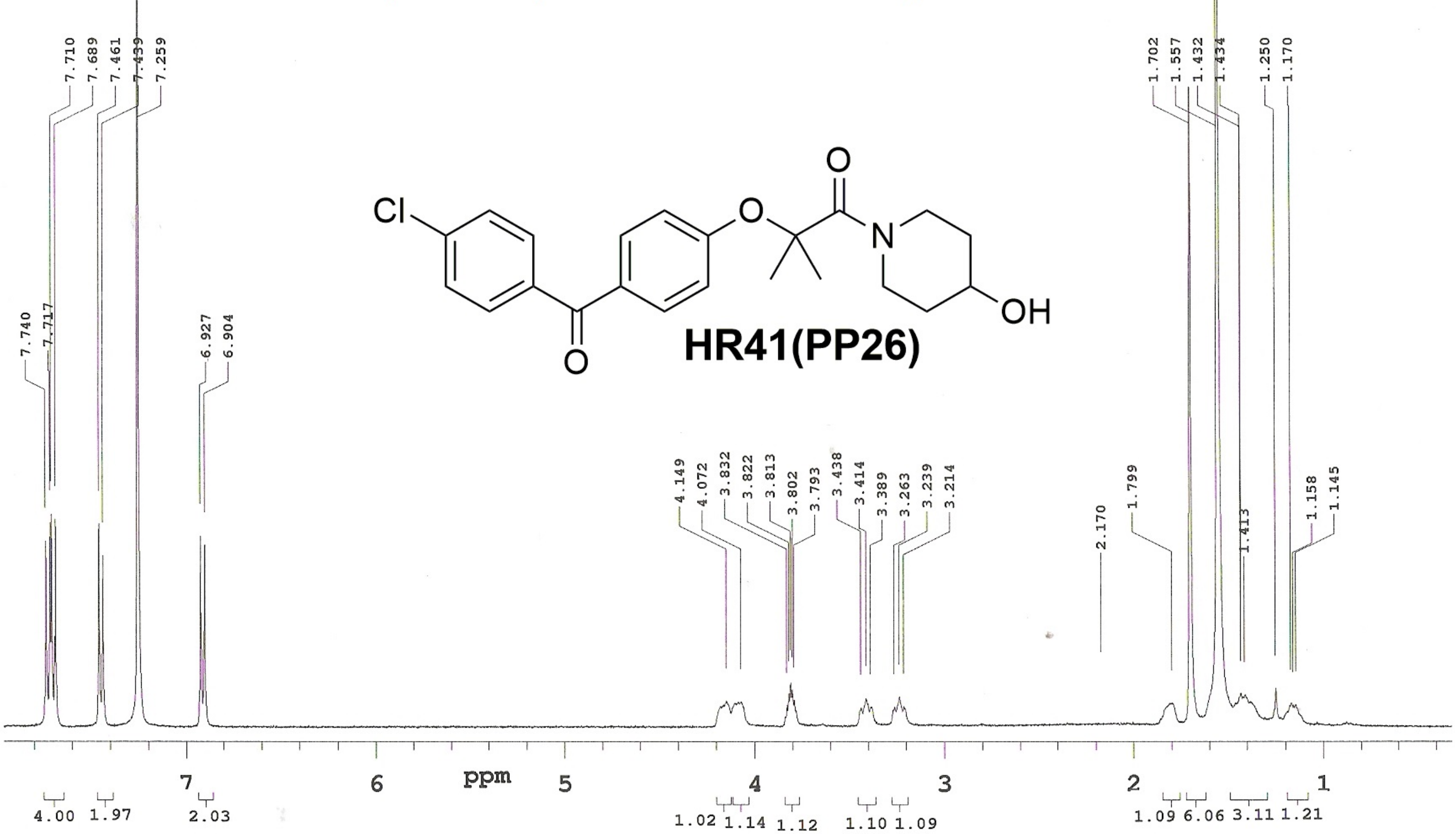

HR41(PP26)

^{1}H-NMR ($CDCl_3$) Varian Mercury 400 Plus

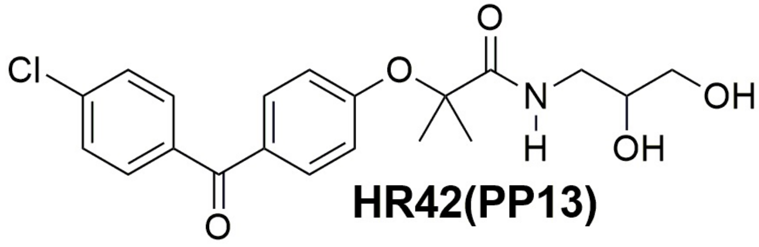

HR42(PP13)

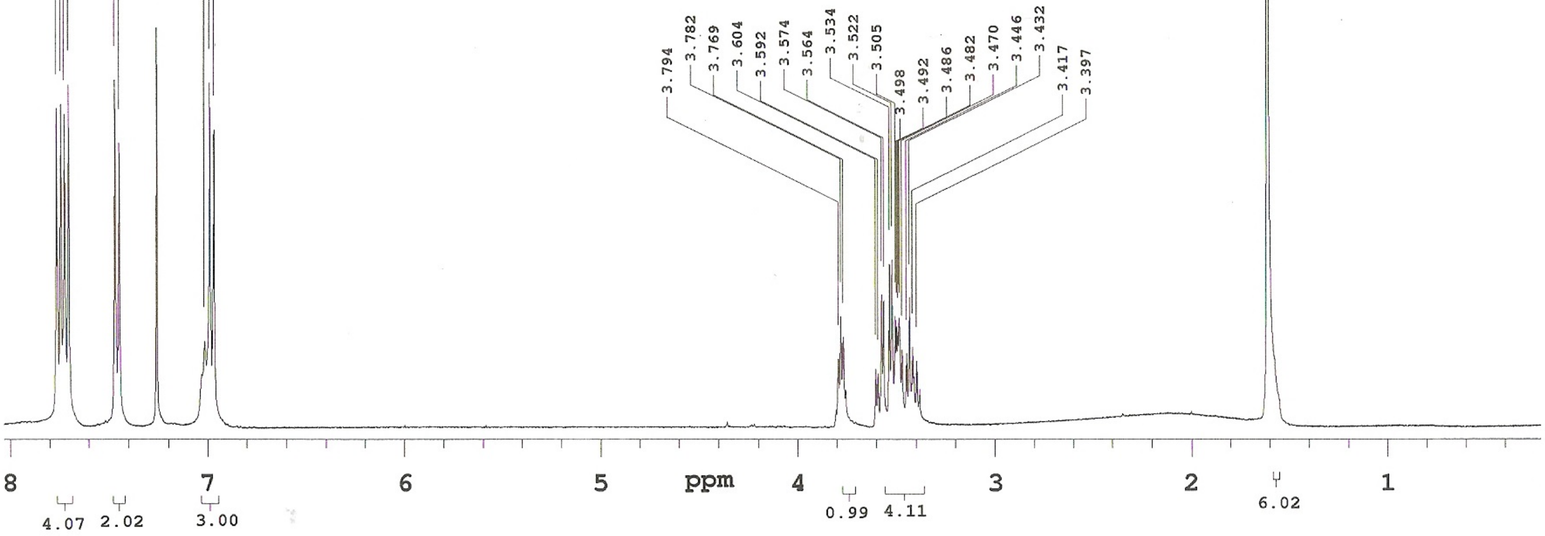

^{1}H-NMR ($CDCl_3$) Varian Mercury 400 Plus

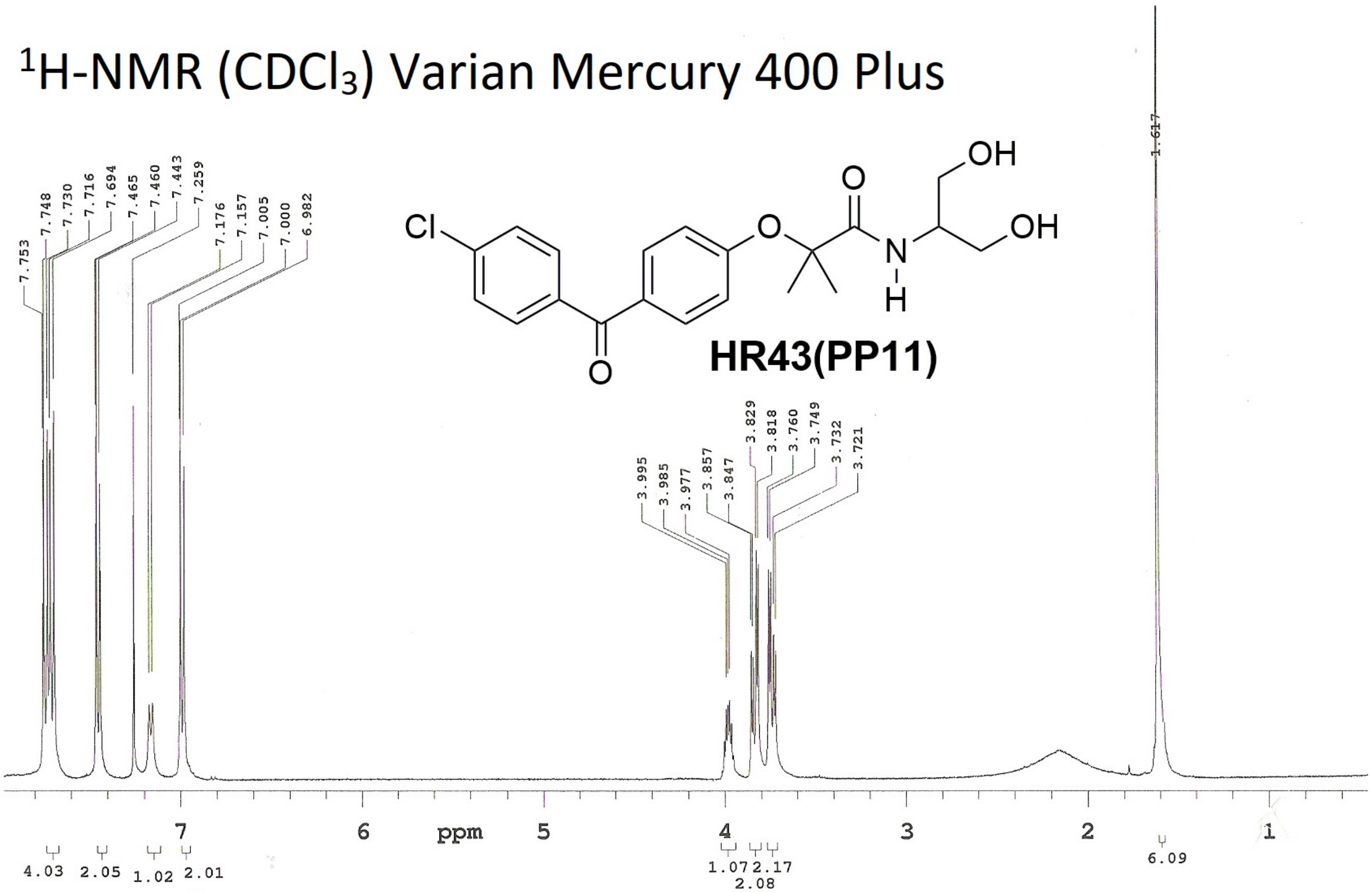

^{1}H-NMR ($CDCl_3$) Varian Mercury 400 Plus

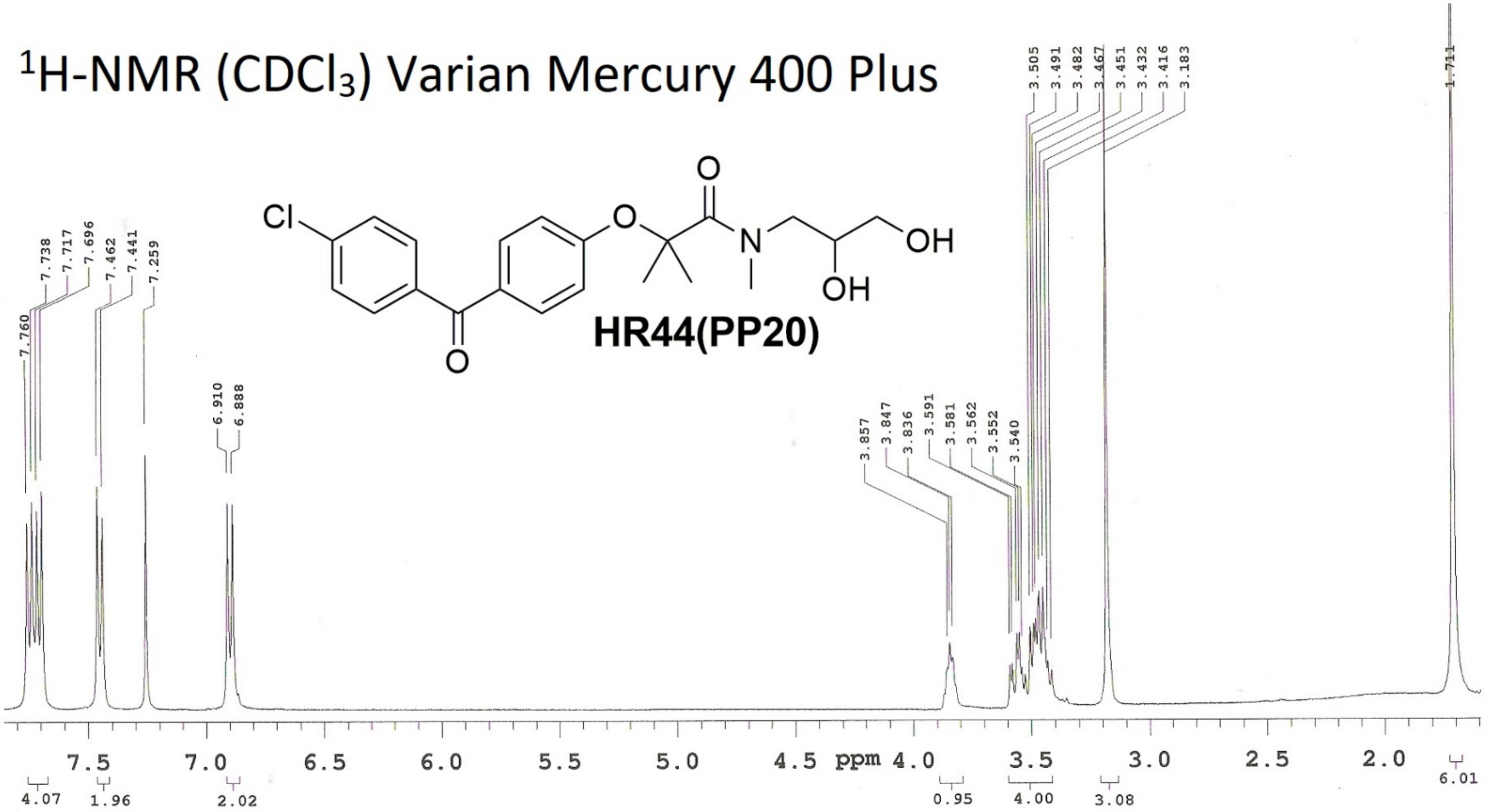

HR44(PP20)

^{1}H-NMR (DMSO-d_6) Varian Mercury 400 Plus

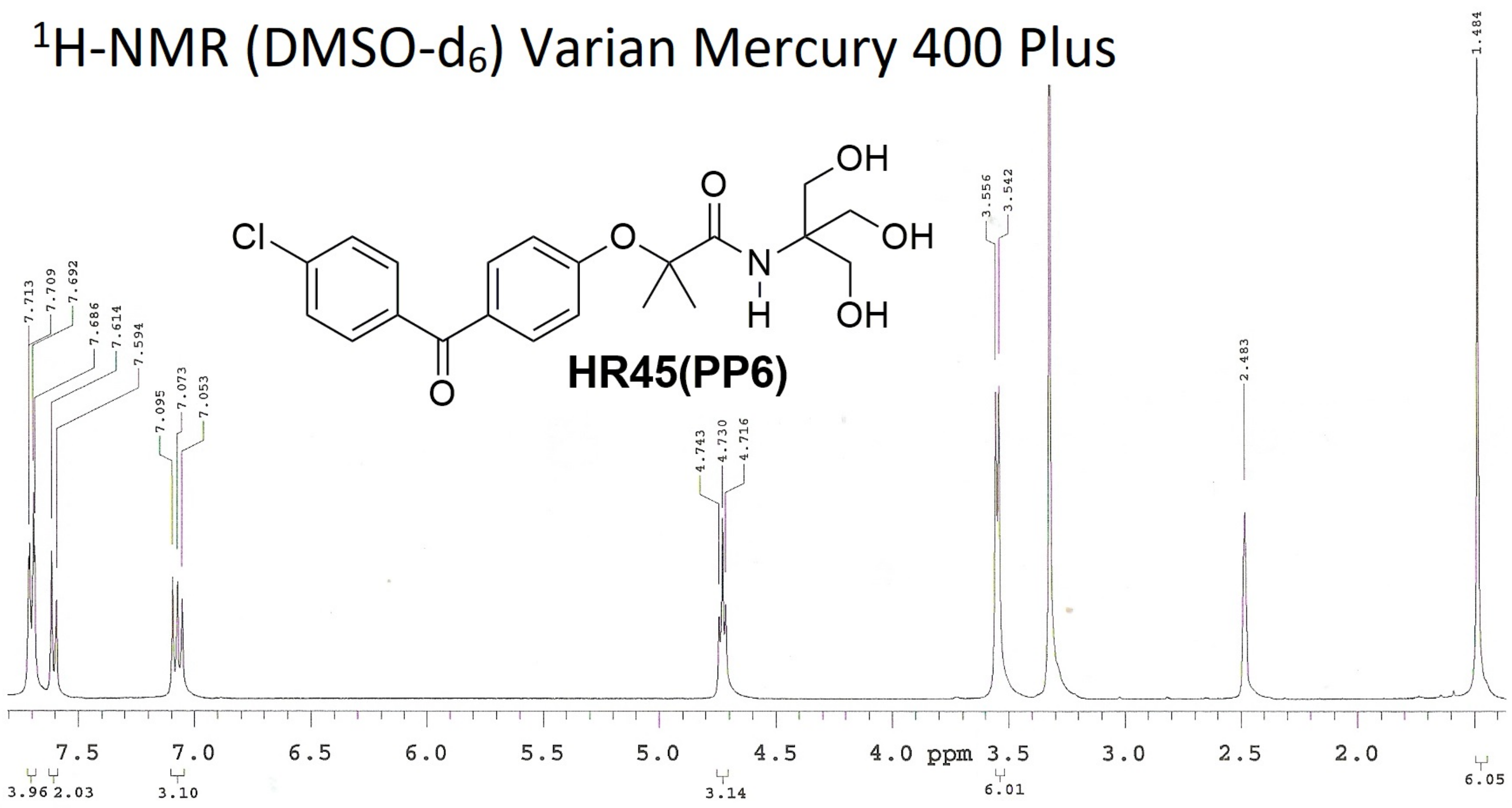

^{1}H-NMR ($CDCl_3$) Varian Mercury 400 Plus

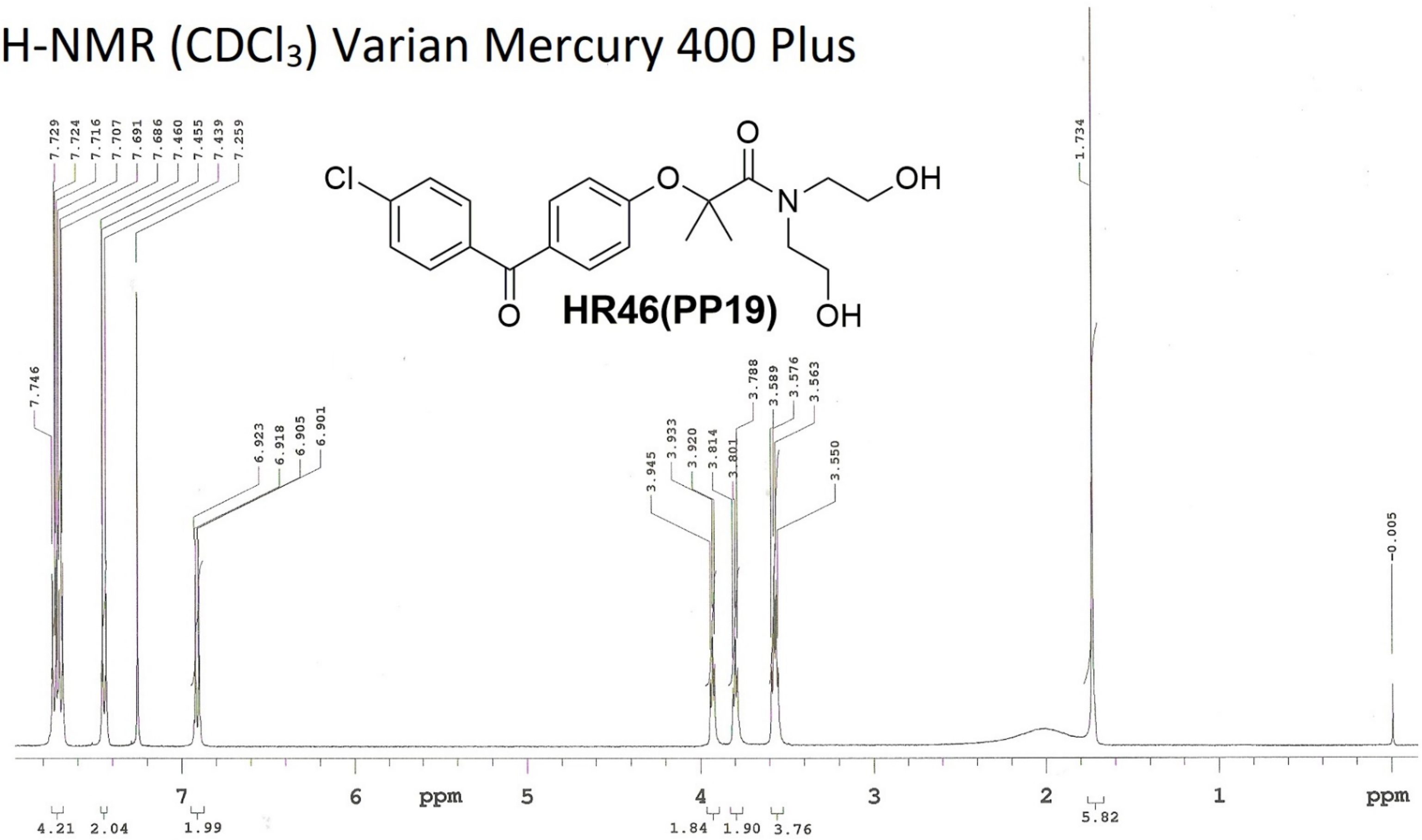

^{1}H-NMR($CDCl_3$) Varian Mercury plus 400 MHz

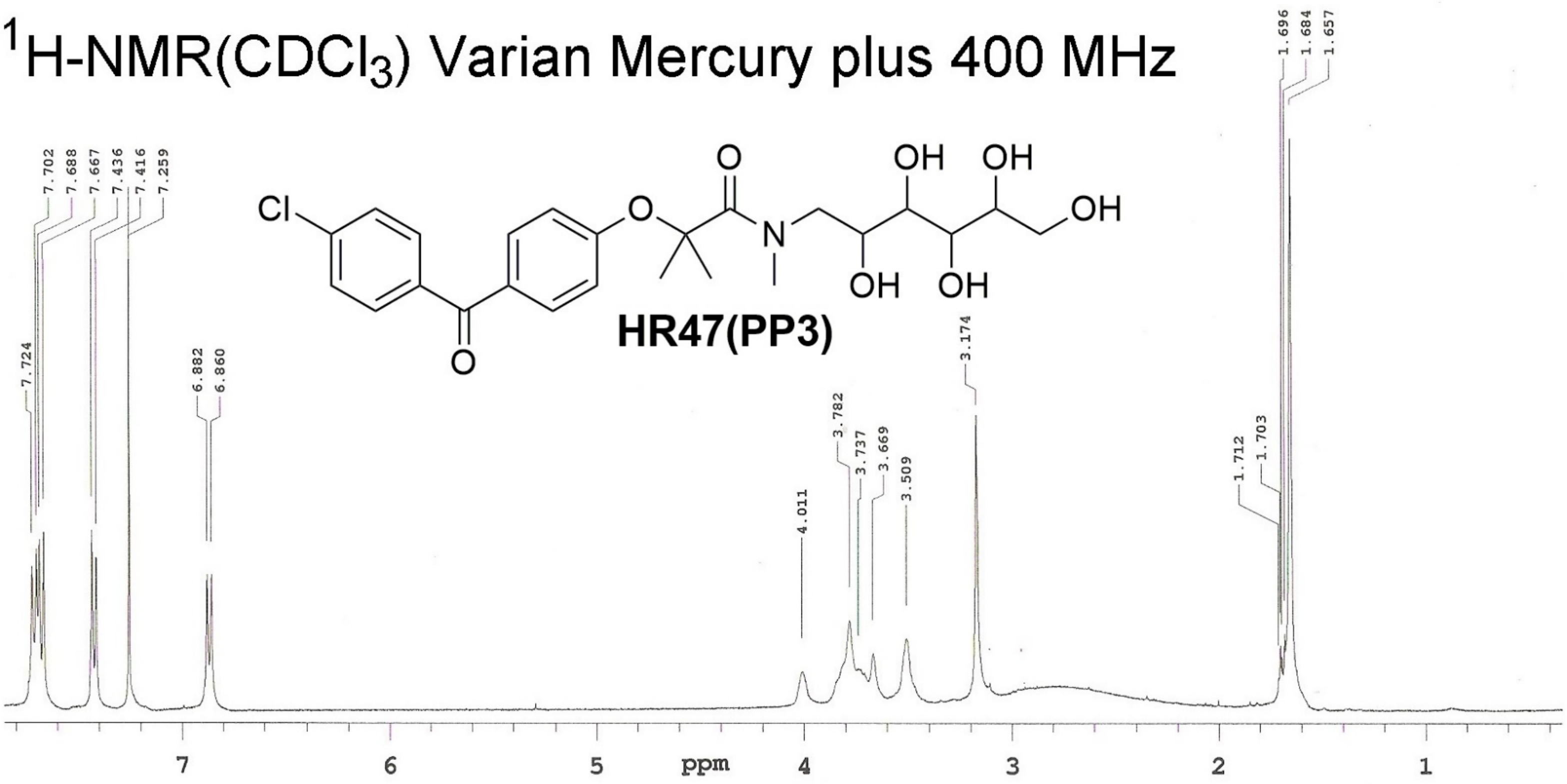

Electrospray Mass Spectra of Few Selected **HR** compounds

TOF MS ES$^+$ (5 µL loop injection, 100% MeCN, 0.2 ml/min)				
Sample	Monoisotopic Mass (M) (Da)	Observed Mass		Formula
**HR34(PP2)**	400	401	[M + H$^+$]	$C_{22}H_{25}Cl_2N_2O_3$
**HR35(PP2HCl)**	436	401	[M – Cl$^-$]	$C_{22}H_{26}Cl_2N_2O_3$
**HR36(PP2MeI)**	542	415	[M – I$^-$]	$C_{23}H_{28}ClIN_2O_3$
**HR37(PP4)**	470	471	[M + H$^+$]	$C_{26}H_{31}ClN_2O_4$
**HR38(PP4HCl)**	506	471	[M – Cl$^-$]	$C_{26}H_{32}Cl_2N_2O_4$
**HR40(PP1)**	375	376	[M + H$^+$]	$C_{20}H_{22}ClNO_4$
**HR47(PP3)**	495	496	[M + H$^+$]	$C_{24}H_{30}ClNO_8$

The MS was a Waters LCT Premier XE (that's a Tof MS) with an ESI source, scanning 100-2000 m/z with direct injections of 5 µl sample, using a 0.2 ml/min flow of acetonitrile

HR34(PP2)

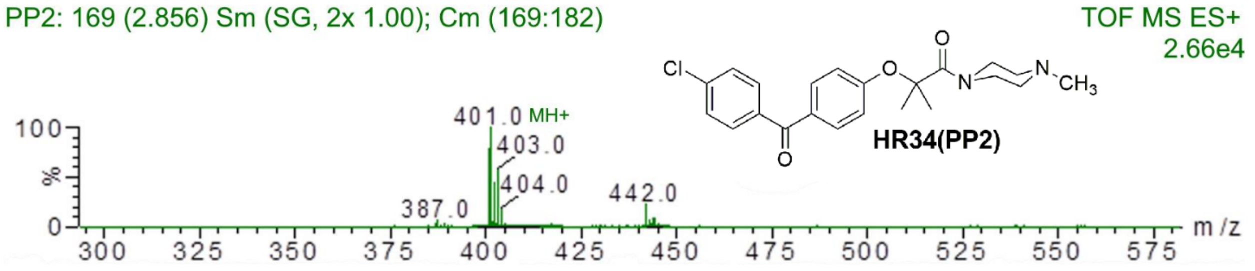

HR35(PP2HCl)

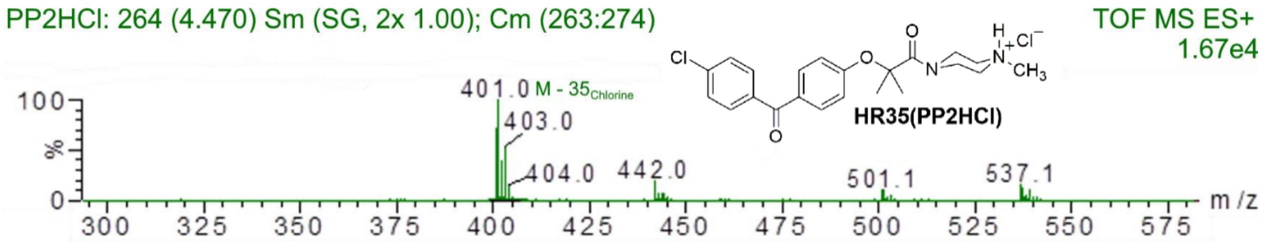

HR36(PP2MeI)

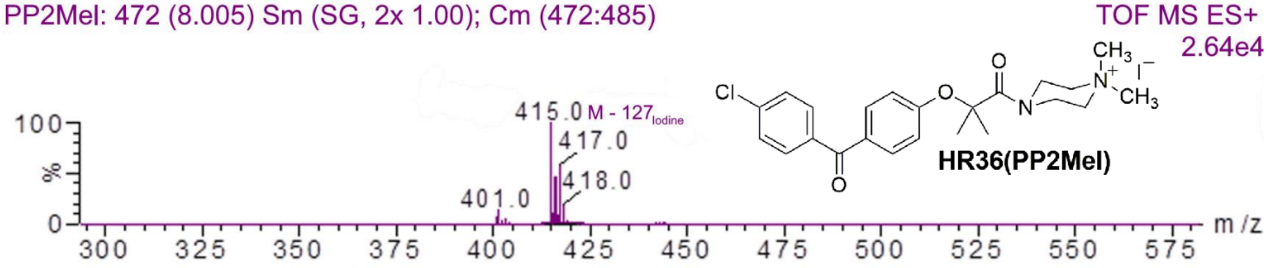

HR37(PP4)

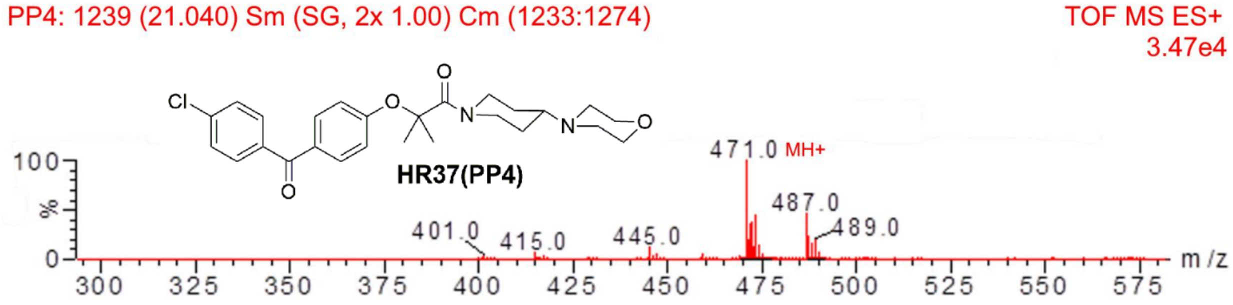

HR38(PP4HCl)

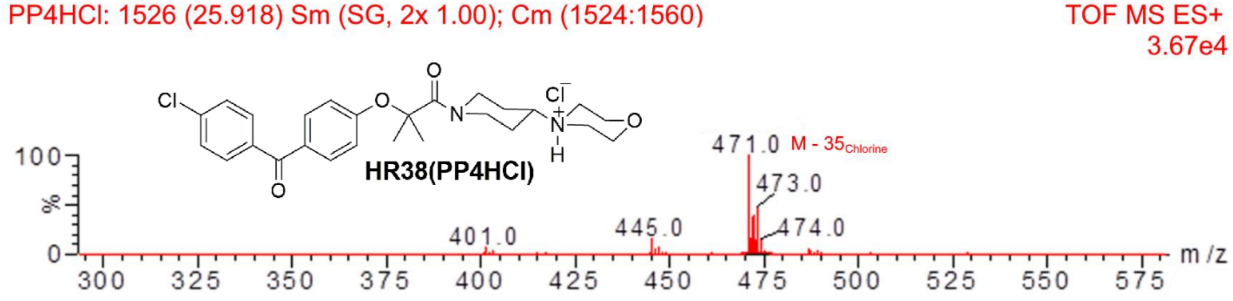

HR40(PP1)

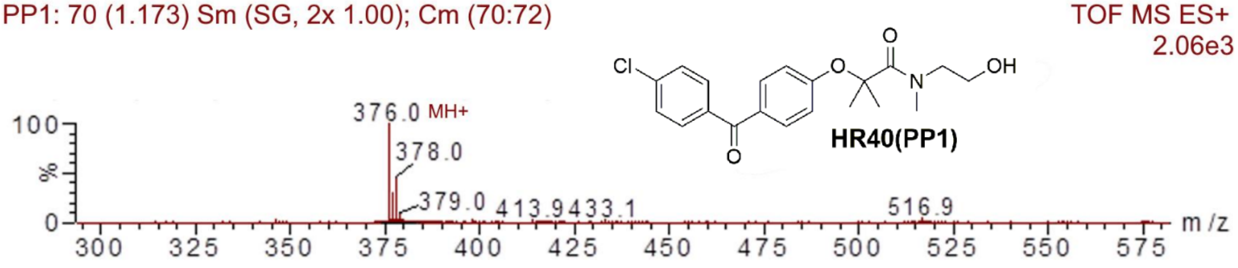

HR47(PP3)

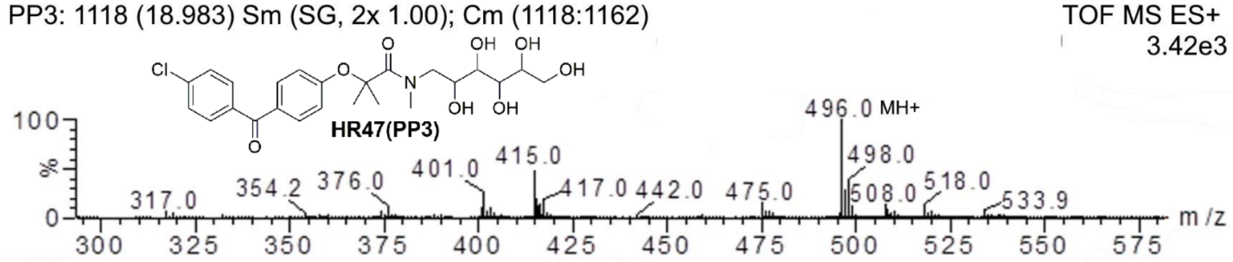
PP3: 1118 (18.983) Sm (SG, 2x 1.00); Cm (1118:1162)
TOF MS ES+
3.42e3
Cl
O
OH
OH
OH
OH
OH
N
HR47(PP3)
496.0 MH+
498.0
415.0
401.0
376.0
354.2
317.0
417.0
442.0
475.0
508.0
518.0
533.9
100
%
0
300
325
350
375
400
425
450
475
500
525
550
575
m/z
